# Marine-Derived Natural Compounds in Respiratory Diseases: A Systematic Review of Preclinical Mechanisms, Clinical Evidence, and Translational Gaps

**DOI:** 10.3390/md24100358

**Published:** 2026-10-13

**Authors:** Valentina Lazzara, Andrea Perri, Alessandra Pandolfo, Paola Pinto, Giustino Carlino, Stefania La Grutta, Elisabetta Pace, Giusy Daniela Albano, Velia Malizia

**Affiliations:** 1 Department of Economics, Business and Statistics (dSEAS), University of Palermo (UNIPA), 90128 Palermo, Italy; alessandra.pandolfo@unipa.it; 2 Institute of Translational Pharmacology (IFT), National Research Council (CNR), Via Ugo La Malfa 153, 90146 Palermo, Italy; andrea.perri@unipa.it (A.P.); paola.pinto01@universitadipavia.it (P.P.); giustino.carlino@unipa.it (G.C.); stefania.lagrutta@ift.cnr.it (S.L.G.); elisabetta.pace@ift.cnr.it (E.P.); giusydaniela.albano@ift.cnr.it (G.D.A.); velia.malizia@ift.cnr.it (V.M.); 3 Department of Earth and Marine Sciences (DiSTeM), University of Palermo (UNIPA), 90123 Palermo, Italy; 4 PhD National Program in One Health Approaches to Infectious Diseases and Life Science Research, Department of Public Health, Experimental and Forensic Medicine, University of Pavia, 27100 Pavia, Italy; 5 Department of Culture and Society, University of Palermo (UNIPA), 90128 Palermo, Italy

**Keywords:** marine natural products, asthma, COPD, pulmonary fibrosis, systematic review, meta-analysis

## Abstract

Marine-derived natural compounds have been investigated across respiratory diseases, but evidence remains fragmented across heterogeneous interventions, models, and outcomes. We mapped the evidence, examined biological convergence, and quantitatively synthesized comparable preclinical data. PubMed/MEDLINE, Scopus, and Web of Science were searched for studies published from 2000 to 2025; random-effects meta-analyses (Hedges’ g) were conducted for model–outcome combinations reported by at least three independent studies, with risk of bias and certainty assessed using design-specific tools and an adapted GRADE approach. Sixty-five studies were included (44 in vivo, 15 in vitro, 5 randomized trials, 1 non-randomized clinical study), with recurrent effects on inflammatory signalling, oxidative stress, immune regulation, tissue injury, and remodelling. Meta-analysis of 17 preclinical studies (60 comparisons, 11 outcomes) favoured marine-derived interventions for eosinophils, IgE-related outcomes, and type 2 cytokines in allergic airway models and for inflammatory/lung-injury markers in acute lung injury models, although heterogeneity was frequent and certainty was very low for all outcomes; clinical evidence was limited and heterogeneous. Marine-derived natural compounds show recurrent biological and directional convergence in selected preclinical respiratory models, but the evidence does not establish a therapeutic class effect or support direct clinical inference; translation requires standardized compound characterization, independent replication, and rigorous candidate prioritization.

## 1. Introduction

Respiratory diseases encompass a heterogeneous group of conditions and remain a major cause of morbidity and mortality worldwide. Chronic respiratory diseases (CRDs), including chronic obstructive pulmonary disease (COPD), asthma, and interstitial lung diseases, accounted for approximately 55.2 million incident cases and 4.4 million deaths globally in 2021, with COPD responsible for the largest share of deaths, despite sustained declines in age-standardized rates over recent decades [1]. COPD remains a leading cause of death worldwide, while asthma is the most prevalent CRD and continues to show marked inequalities in disease burden and access to effective care [2,3,4]. Lower respiratory infections remain an important cause of acute morbidity and mortality, particularly among children and older adults [5], and environmental air pollution contributes substantially to respiratory disease burden [6].

Despite their clinical heterogeneity, many respiratory diseases involve interconnected pathological processes, including persistent or dysregulated inflammation, oxidative stress, immune dysfunction, epithelial and endothelial injury, and tissue remodeling [7,8,9]. Existing therapies have substantially improved outcomes in several respiratory conditions, but important unmet needs remain. In COPD, current and emerging treatment strategies increasingly target specific treatable traits, yet preventing disease progression remains challenging [7,10]. Similarly, antifibrotic therapies slow functional decline in progressive fibrosing interstitial lung diseases but do not reverse established fibrosis [11]. Long-term treatment of CRDs may also be affected by economic burden, adverse effects and suboptimal adherence [12,13]. These persistent gaps provide a rationale for investigating bioactive molecules capable of modulating multiple interacting components of respiratory pathobiology.

Marine ecosystems represent a chemically diverse source of natural products generated under distinctive ecological and environmental pressures [14]. Macroalgae, microalgae, marine invertebrates, and marine microorganisms produce structurally diverse metabolites, including sulfated polysaccharides, carotenoids, polyphenols and phlorotannins, lipids and fatty-acid derivatives, peptides, pigments, alkaloids, and other specialized molecules [14,15,16]. Experimental research has attributed anti-inflammatory, antioxidant, immunomodulatory, and other biologically active properties to compounds from these sources [15,16]. Such activities are potentially relevant to respiratory tissues, which are continuously exposed to allergens, pathogens, pollutants, and other environmental stressors that can induce inflammatory and oxidative injury [8,9]. However, the term “marine-derived natural compounds” encompasses pharmacologically heterogeneous interventions, ranging from chemically defined molecules to enriched fractions, lipid extracts, polysaccharide preparations, crude extracts, and whole-organism or multicomponent preparations. Evidence of biological activity across this spectrum cannot therefore be assumed to indicate pharmacological equivalence or a common therapeutic class.

The central problem is not the scarcity of positive experimental findings but the fragmentation and interpretability of the evidence. Studies are distributed across different respiratory disease contexts, compound classes, experimental systems, exposure protocols, doses, biological compartments, and outcomes. Much of the literature consists of small preclinical studies using short-term models and surrogate inflammatory or oxidative-stress markers. In addition, incomplete reporting of randomization, blinding, attrition, intervention characterization, and other safeguards against bias may limit confidence in reported effects. For complex natural products, variation in source organism, geographical origin, harvesting conditions, extraction procedures, molecular composition, and batch standardization creates an additional challenge for reproducibility and translation, consistent with broader challenges in marine natural-product discovery and development [14,16]. Consequently, it remains unclear whether the literature represents a collection of isolated positive experiments or whether recurrent biological effects converge across independent compounds and experimental models. This uncertainty is particularly relevant to translation. Mechanistic convergence across diverse studies may identify biologically recurrent pathways and inform candidate prioritization, but it does not establish clinical efficacy. Similarly, large effects in standardized animal models may reflect pharmacological activity while remaining vulnerable to model dependence, methodological limitations, small-study effects, and indirectness with respect to human disease. The limited and heterogeneous clinical literature further complicates assessment of whether preclinical findings have progressed along a coherent translational pathway. An integrated evaluation therefore requires not only mapping the breadth of the evidence but also distinguishing levels of evidence, assessing study-level risk of bias, determining when quantitative synthesis is justified, and evaluating the certainty and limits of the resulting estimates.

Previous literature has largely described the chemical diversity and pharmacological potential of marine natural products or examined individual compounds and disease applications [14,15,16]. A cross-disease synthesis focused specifically on respiratory research is therefore needed to determine what the field has studied whether reported effects converge at the level of biological processes, which findings are sufficiently comparable for quantitative evaluation, and what the available evidence can and cannot support regarding therapeutic translation. Such an approach is especially important in a chemically heterogeneous field, in which repeated modulation of common inflammatory, or antioxidant pathways may indicate recurrent biological activity without demonstrating interchangeability among interventions.

We therefore systematically reviewed preclinical and clinical studies of marine-derived natural compounds across major respiratory disease contexts. The objectives were to: (1) map the distribution of evidence across study designs, marine sources, compound classes, and respiratory applications; (2) characterize recurrent biological and mechanistic patterns across experimental systems; (3) quantitatively synthesize selected preclinical model–outcome combinations when studies were sufficiently comparable; (4) assess study-level risk of bias using design-specific tools; and (5) evaluate the certainty of the meta-analyzed preclinical evidence and define the current limits of therapeutic and translational inference.

## 2. Results

### 2.1. Study Selection

**Figure 1:**
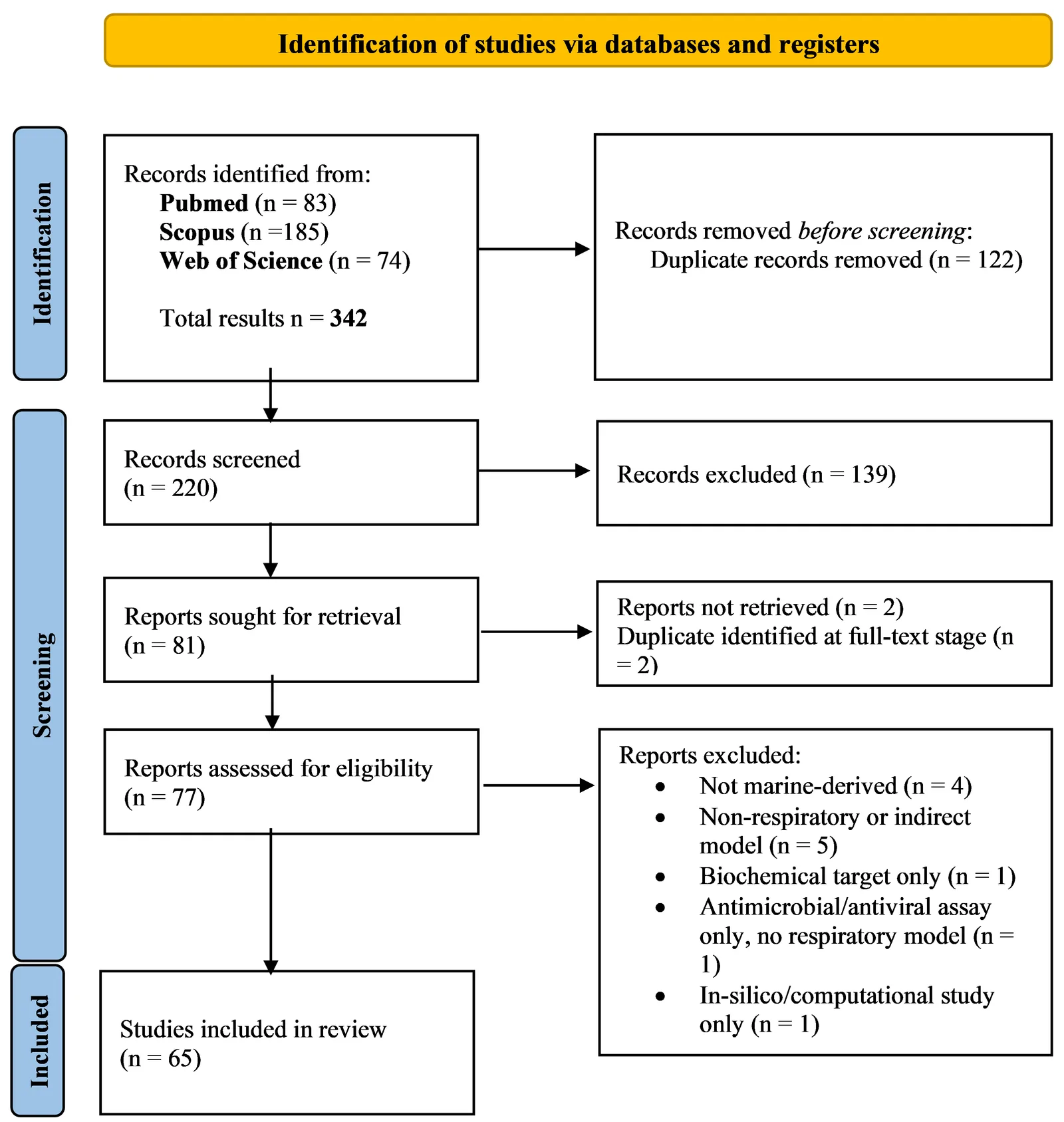
PRISMA 2020 flow diagram of the study selection process. Adapted from [17].

The literature search identified a total of 342 records across PubMed/MEDLINE, Scopus, and Web of Science. After removal of 122 duplicate records, 220 records were screened by title and abstract, of which 139 were excluded. Full-text articles were sought for 81 records; two full texts could not be retrieved, and two additional duplicates were identified during full-text assessment. Therefore, 77 full-text articles were evaluated for eligibility. Twelve articles were excluded, leaving 65 unique studies for qualitative synthesis. The study selection process is summarized in the PRISMA flow diagram (Figure 1).

### 2.2. Study Characteristics and Evidence Map

The 65 included studies were published between 2000 and 2025 and covered a broad range of marine-derived compounds, preparations, experimental systems, and respiratory disease contexts. Using the prespecified single-design classification hierarchy, the evidence base comprised 44 studies with an in vivo component, 15 pure in vitro studies, 5 randomized clinical trials, and 1 non-randomized clinical study. Studies combining in vitro and in vivo experiments were classified as in vivo for evidence mapping and risk-of-bias partitioning, while their mechanistic in vitro findings were retained in the narrative synthesis.

**Figure 2:**
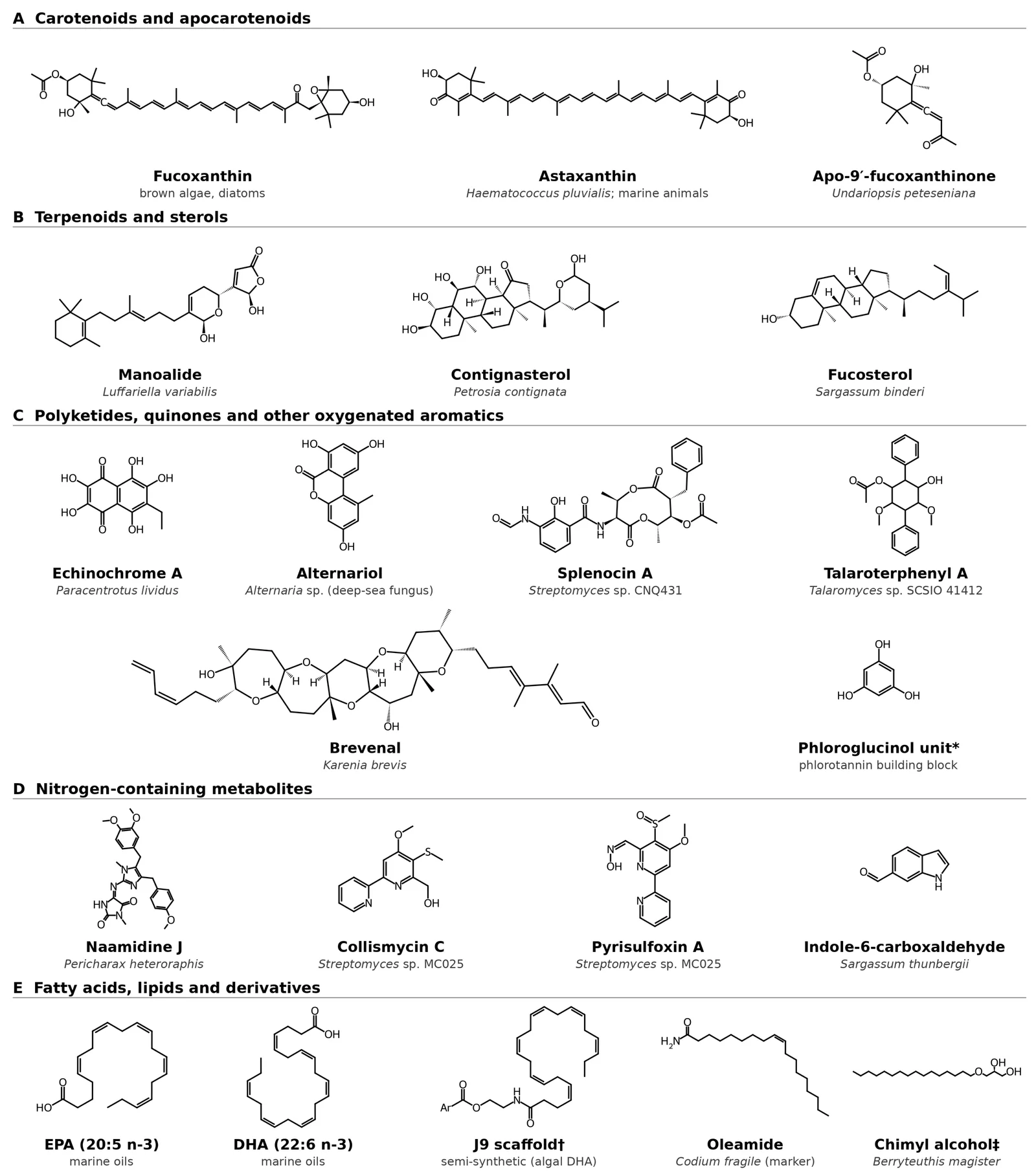
Chemical structures of representative low-molecular-weight marine-derived compounds evaluated in the included studies, grouped by structural class: (**A**) carotenoids and apocarotenoids; (**B**) terpenoids and sterols; (**C**) polyketides, quinones, and other oxygenated aromatics; (**D**) nitrogen-containing metabolites; and (**E**) fatty acids, lipids, and derivatives. The marine source is reported below each structure, and the corresponding references are as follows: fucoxanthin [18,19,20]; astaxanthin [21,22]; apo-9′-fucoxanthinone [23]; manoalide [24]; contignasterol [25]; fucosterol [26]; echinochrome A [27,28]; alternariol [29]; splenocin A [30]; talaroterphenyl A [31]; brevenal [32]; naamidine J [33]; collismycin C [34]; pyrisulfoxin A [34]; indole-6-carboxaldehyde [35]; EPA [36,37,38]; DHA [36,37,38,39]; oleamide [40]. Structures were drawn from PubChem records or from the original structural reports. * Phloroglucinol is shown as the monomeric building block of the phlorotannins trifuhalol A and eckmaxol [41,42]; † J9 is shown as the general scaffold of the semi-synthetic substituted-benzoate esters of DHA ethanolamine (Ar, substituted phenyl group) [43]; ‡ chimyl alcohol (1-O-hexadecylglycerol) is the main constituent of the squid-derived 1-O-alkylglycerol preparation, together with batyl alcohol [44]. Macromolecules (sulfated polysaccharides, laminarin, C-phycocyanin, and phycocyanin-derived peptides), saponin-rich extracts, and crude or multicomponent preparations are not depicted.

The evidence base was therefore predominantly preclinical. Clinical evidence was limited and concentrated mainly in asthma and allergic airway disease, with heterogeneous interventions and endpoints. The most frequently investigated disease contexts were asthma and allergic airway diseases (28 studies), followed by acute lung injury/ARDS (14), pulmonary fibrosis and lung remodeling (8), respiratory infections (5), and pollution- or exposure-related airway injury (10). Marine sources included macroalgae, microalgae and cyanobacteria, marine invertebrates, marine-derived microorganisms, and marine lipid or polysaccharide preparations. The principal compound classes were sulfated polysaccharides, carotenoids, polyphenols and phlorotannins, fatty acids and lipid extracts, peptides, pigments, and other structurally diverse marine metabolites. The structural diversity of the low-molecular-weight compounds evaluated in the included studies, spanning carotenoids and apocarotenoids, terpenoids and sterols, polyketides and quinones, nitrogen-containing heterocycles, and fatty-acid derivatives is illustrated in Figure 2. Several of these molecules possess uncommon scaffolds, such as the polyether ladder of brevenal, the 2,2′-bipyridine core of collismycin C and pyrisulfoxin A, the 2-aminoimidazole–hydantoin framework of naamidine J, and the allenic, epoxide-bearing skeleton of fucoxanthin, and may therefore represent starting points for semi-synthetic optimization and structure–activity studies. Key characteristics of the included studies are summarized in Supplementary Table S1A–E.

### 2.3. Marine-Derived Compounds in Asthma and Allergic Airway Diseases

Asthma and allergic airway diseases represented the largest disease domain in the evidence base, comprising 28 studies. The evidence encompassed mechanistic cellular systems, predominantly OVA-based animal models, and a limited number of clinical studies. Despite substantial heterogeneity in marine sources and preparations, the preclinical findings clustered around four recurrent biological processes: suppression of type 2 inflammation, attenuation of oxidative stress, modulation of regulatory immune responses, and inhibition of pathways involved in airway remodeling.

In cellular and ex vivo systems, structurally diverse marine-derived compounds affected several components of allergic airway inflammation. Oligo-fucoidan inhibited PDGF-induced airway smooth-muscle-cell proliferation through suppression of ERK1/2, Akt, and NF-κB signaling [45], whereas the sponge-derived metabolite manoalide reduced mast-cell inflammatory mediator release through inhibition of caspase-1, NF-κB, and MAPK activation [24]. Marine microbial metabolites also modulated adaptive immune responses: splenocins suppressed IL-5 and IL-13 production at low concentrations [30], while alternariol affected early T-cell activation, cytokine production, and inflammatory-cell migration [29]. Collectively, these studies suggested activity at multiple cellular levels relevant to allergic airway disease, although they used heterogeneous experimental systems and did not establish a common molecular target.

Most in vivo evidence was generated in OVA-induced allergic airway models. Across sulfated polysaccharides, algal polyphenol-rich extracts, phlorotannins, carotenoids, marine pigments, and lipid-rich preparations, a recurrent pattern was observed: reductions in airway or bronchoalveolar eosinophilia, type 2 cytokines, IgE-related outcomes, mucus production, and, when measured, airway hyperresponsiveness. Fucoidan and several macroalgal extracts primarily suppressed IL-4, IL-5, IL-13, eosinophilic inflammation, and IgE responses [40,41,46,47,48,49,50]. Marine lipid preparation (Lyprinol, a green-lipped mussel extract) similarly reduced airway inflammation and improved lung function [51]. Echinochrome and fucoxanthin combined anti-inflammatory effects with restoration of antioxidant defences and reductions in oxidative-stress markers [18,27,28]. Other preparations appeared to act through broader immunoregulatory mechanisms. Sea-cucumber-derived extracts and lipid fractions increased IL-10, TGF-β, and Foxp3-positive regulatory T-cell responses while reducing Th2- and, in some models, Th17-associated inflammation [52,53]. In OVA models complicated by particulate-matter exposure, *Sargassum horneri* preparations attenuated mixed inflammatory responses through MAPK inhibition, Nrf2/HO-1 activation, and modulation of Th2/Th17-related signaling [54,55].

Additional experimental models extended these findings beyond conventional lower-airway OVA asthma. Contignasterol reduced allergen-induced plasma exudation in sensitized guinea-pig airways [25], suggesting an effect on allergen-associated microvascular leakage. *Undaria pinnatifida* extract attenuated an OVA-induced allergic rhinitis model, rebalancing Th1/Th2 and Th17/Treg responses and suppressing NF-κB/MAPK signaling [56]. In a combined allergic rhinitis–asthma syndrome (CARAS) model, the same extract additionally preserved epithelial-barrier integrity, downregulated IL-33, and enhanced Nrf2/HO-1-related antioxidant responses [57]. These studies broadened the biological contexts represented within the domain but remained preclinical.

Overall, convergence across the animal studies was greater at the level of biological response than at the level of compound identity. Suppression of eosinophilic and type 2 inflammation was the most recurrent signal, accompanied in subsets of studies by antioxidant activity, Treg-associated immune modulation, protection of epithelial integrity, or inhibition of NF-κB, MAPK, and remodeling-related pathways. However, the evidence was heavily concentrated in short-term allergen-challenge models, particularly OVA-induced disease, and several tested interventions were crude extracts or incompletely standardized mixtures. The quantitative synthesis of selected OVA model–outcome combinations is reported separately in Section 2.9.

Clinical evidence was limited to six studies and was heterogeneous in design, intervention, population, and outcome (Table 1). Three randomized studies evaluated marine lipid preparations in asthma. Lyprinol improved selected symptom, peak-flow, oxidative-stress, and exercise- or hyperpnea-induced bronchoconstriction outcomes in two small trials [36,37]. In the parallel-group trial (46 adults with steroid-naïve atopic asthma), 8 weeks of Lyprinol reduced daytime wheeze and exhaled H_2_O_2_ and increased morning PEF relative to an olive-oil placebo, without changes in FEV1 [36]. In the crossover trial (20 adults with hyperpnea-induced bronchoconstriction), 3 weeks of the *Perna canaliculus* lipid fraction PCSO-524 attenuated the maximum post-challenge fall in FEV1 (−8.4% vs. −22.5% with placebo) and reduced exhaled cysteinyl leukotrienes and 8-isoprostane, symptom scores and rescue-medication use [37]. In contrast, a multicenter placebo-controlled trial of salmon oil in type 2 eosinophilic asthma found no significant benefit for its primary composite exacerbation outcome or for FEV1 and FeNO [38]. In this exploratory trial (70 participants randomized, 66 analyzed; 6 g/day for 20 weeks), the median time to the first composite event was 37 versus 15 days (*p* = 0.347), 73% versus 82% of participants experienced at least one event, and ACQ-5 scores did not differ between groups [38]. A small uncontrolled study of squid-derived alkylglycerols reported improvements in asthma control, selected lung-function measures, inflammatory cytokines, and lipid mediators (0.4 g/day for 3 months, added to budesonide/formoterol in 19 obese patients with mild asthma; clinically relevant ACQ improvement in 17 of 19 patients) [44], but its open-label before–after design and serious risk of bias preclude causal interpretation. Two randomized studies of *Spirulina in* allergic rhinitis reported improvement in nasal symptoms or reduction in IL-4 production [58,59]. In the larger trial, 150 adults were randomized to 2 g/day of Spirulina or placebo for 6 months, with 129 completing the study and unequal completion between arms, and outcomes were largely self-reported symptom scores [58]; in the smaller crossover study (36 adults, 12 weeks), a 32% reduction in IL-4 production by stimulated peripheral blood mononuclear cells was observed only at 2 g/day, and no clinical endpoints were assessed [59]. Where reported, the interventions were well tolerated, with no significant side effects in the parallel-group Lyprinol trial [36] and no between-group differences in adverse events or withdrawals due to side effects in the salmon-oil trial [38]; however, none of the studies was designed or powered to assess safety. Across the six studies, sample sizes ranged from 19 to 150 participants and intervention durations from 3 weeks to 6 months; only two trials were prospectively registered [37,38], only one was judged at low risk of bias [37], and the trial with an exacerbation-based primary endpoint was neutral [38]. Overall, the clinical findings did not reproduce a single consistent efficacy signal and could not be quantitatively pooled.

ACQ, Asthma Control Questionnaire; b.i.d., twice daily; DHA, docosahexaenoic acid; EBC, exhaled breath condensate; EPA, eicosapentaenoic acid; EVH, eucapnic voluntary hyperpnea; FeNO, fractional exhaled nitric oxide; FEV1, forced expiratory volume in 1 s; MCT, medium-chain triglycerides; PBMCs, peripheral blood mononuclear cells; PEF, peak expiratory flow; PHA, phytohaemagglutinin; PUFA, polyunsaturated fatty acids; RCT, randomized controlled trial; RoB 2, revised Cochrane risk-of-bias tool for randomized trials; ROBINS-I, Risk Of Bias In Non-randomized Studies of Interventions; VC, vital capacity. Domain-level risk-of-bias judgements are reported in Supplementary Tables S4 and S5.

### 2.4. Marine-Derived Compounds in Acute Lung Injury and ARDS

The acute lung injury and ARDS domain comprised 14 studies and was almost entirely preclinical. Most studies used LPS-induced inflammatory models, whereas a smaller number investigated sepsis-associated, toxic, hyperoxic, or other forms of acute lung injury. Compared with asthma literature, the biological response was more consistently centered on macrophage activation, pro-inflammatory cytokine production, oxidative injury, pulmonary edema, and inflammasome signaling.

Cellular studies identified several distinct upstream mechanisms. Naamidine J, a sponge-derived imidazole alkaloid, reduced macrophage inflammatory activation through the CSE1L–SP1 axis [33], whereas J9, a semi-synthetic derivative of docosahexaenoic acid ethanolamine (DHA-EA, N-docosahexaenoylethanolamine) obtained by esterifying a substituted benzoyl group to the terminal hydroxyl of DHA-EA prepared from DHA-rich micro-algal oil, suppressed NF-κB-dependent cytokine production through a Nur77-dependent mechanism [43]. Because J9 is a semi-synthetic derivative and not a native marine metabolite, its activity should not be attributed to a naturally occurring compound; however, it was retained because its DHA scaffold was obtained directly from a marine algal source. Although these compounds acted through different proximal targets, their effects were consistent with the broader in vivo evidence showing convergence on attenuation of innate inflammatory signaling. In LPS-induced animal models, sulfated polysaccharides, β-glucans, carotenoids, algal pigments, and marine extracts generally reduced inflammatory-cell recruitment, pulmonary edema, lung injury, and pro-inflammatory cytokine production. Fucoidan attenuated dendritic-cell activation and inflammatory responses [60], while laminarin modulated macrophage polarization through HIF-1α-related signaling [61]. Aerosolized algal polysaccharides combined suppression of TLR4/NF-κB signaling with activation of the Nrf2/HO-1 antioxidant response [62]. C-phycocyanin, fucoxanthin, *Endarachne binghamiae* extract, and sea-cucumber-derived preparations also reduced inflammatory mediators and histological injury, with recurrent involvement of NF-κB, MAPK, PI3K/AKT, TLR4/MyD88, and NLRP3-related pathways [19,63,64,65,66]. For combination products containing both marine and non-marine constituents, however, attribution of the observed effects to the marine component alone was not possible.

A smaller group of studies used alternative acute-injury models. A marine probiotic attenuated inflammatory and oxidative injury in sepsis-associated lung injury [67], a brown-algal extract reduced oxidative stress and apoptosis in copper-induced toxic injury [68], and fucoidan showed protective effects in hyperoxia-induced neonatal lung injury [69]. These models broadened the biological contexts represented but were not directly comparable with the predominant LPS-induced ALI models.

Across the domain, the most consistent preclinical pattern was attenuation of innate immune activation accompanied by reductions in cytokine release, oxidative stress, edema, and tissue injury. NF-κB suppression was the most recurrent signaling feature, frequently accompanied by MAPK inhibition, Nrf2/HO-1 activation, altered macrophage polarization, or NLRP3 inflammasome suppression. Quantitative synthesis was possible for selected LPS model–outcome combinations and is reported in Section 2.9. No clinical intervention studies in patients with ALI or ARDS were identified.

### 2.5. Marine-Derived Compounds in Pulmonary Fibrosis and Lung Remodeling

The pulmonary fibrosis and lung-remodeling domain comprised eight studies and was entirely preclinical. Studies included fibroblast-based systems and animal models of bleomycin- or radiation-induced lung injury. Despite the limited evidence base, the findings converged on three related processes: suppression of fibroblast activation, attenuation of extracellular-matrix deposition, and reduction of oxidative and inflammatory signaling.

In cellular models, talaroterphenyl A inhibited PDE4 activity and reduced expression of fibronectin, collagen I, and α-smooth-muscle actin in TGF-β1-stimulated lung fibroblasts [31]. Other marine-derived compounds acted primarily through antioxidant and cytoprotective pathways. Indole-6-carboxaldehyde and laminarin reduced reactive oxygen species and cellular injury while activating Nrf2/HO-1-related antioxidant responses [35,70], and a phycocyanin-derived peptide similarly modulated the Keap1–Nrf2–HO-1 axis in fibrosis- and remodeling-relevant cellular systems [71].

The in vivo studies extended these findings to experimental tissue remodeling. A polyphenol-rich *Corallina officinalis* extract improved antioxidant defenses and reduced bleomycin-induced fibrotic injury [72]. Fucoxanthin reduced fibroblast activation and extracellular-matrix markers through modulation of TGF-β/Smad and MAPK signaling [20], while *Caulerpa okamurae* extract affected both fibrotic signaling and NLRP3 inflammasome activation [73]. Astaxanthin reduced inflammatory and apoptotic responses in radiation-induced lung injury [21].

Overall, the domain showed a recurrent anti-fibrotic and cytoprotective signal centered on TGF-β/Smad-related fibroblast activation, extracellular-matrix deposition, and Nrf2-associated oxidative-stress responses. However, the small number of studies, diversity of preparations, and predominance of short-term experimental models limited assessment of reproducibility and durability. No clinical studies of marine-derived compounds for pulmonary fibrosis or lung remodeling were identified, and the available evidence did not support quantitative synthesis.

### 2.6. Marine-Derived Compounds in Respiratory Infections

The respiratory-infection domain comprised five studies and was heterogeneous in pathogen, intervention, and experimental design, encompassing influenza, respiratory syncytial virus, SARS-CoV-2, and pneumococcal infection models. The evidence did not identify a single recurrent antiviral or antibacterial mechanism.

In vitro evidence of direct antiviral activity was limited. HESA-A inhibited influenza A virus replication in infected epithelial cells, apparently by interfering with viral adsorption and penetration [74]. HESA-A was retained because it was reported as an herbal-marine preparation; however, the marine component was not taxonomically specified, and attribution of the observed activity to a defined marine constituent was therefore not possible.

In animal models, the predominant pattern was modulation of host response rather than consistent direct suppression of pathogen replication. A combination of cordycepin, lactoferrin, and *Sargassum fusiforme* polysaccharides reduced viral load and lung injury in an RSV model and was associated with altered macrophage polarization [75]. Since this was a multicomponent formulation, the contribution of the marine-derived constituent could not be isolated. In H1N1 influenza, fucoidan reduced clinical disease severity and lung consolidation without significantly reducing viral titers, suggesting attenuation of infection-associated immunopathology rather than direct antiviral activity [76]. A solubilized sturgeon-oil nanoemulsion showed activity across experimental SARS-CoV-2, influenza, and RSV models [39], although interpretation was limited by the proprietary multicomponent formulation and high risk of bias. In bacterial infection, ascophyllan HS improved survival and reduced pulmonary bacterial burden in a pneumococcal model, with evidence of host immune activation [77].

Taken together, the infection studies suggested two potentially distinct patterns: direct interference with pathogen replication or entry in selected experimental systems and modulation of host inflammatory or immune responses in vivo. However, the small number of studies, diversity of pathogens and formulations, frequent use of multicomponent preparations, and absence of clinical trials prevented assessment of consistency across infection types or quantitative synthesis.

### 2.7. Marine-Derived Compounds in Pollution- and Exposure-Related Lung Injury

The pollution- and exposure-related lung-injury domain comprised 10 studies, all preclinical. Studies focused on particulate-matter-induced inflammation, cigarette-smoke-related epithelial injury, and COPD-relevant experimental models. Studies combining allergen sensitization with particulate-matter exposure were classified within the asthma and allergic-airway-disease domain and are therefore discussed in Section 2.3.

In particulate-matter models, the dominant biological pattern was protection against oxidative stress, inflammatory mediator production, and epithelial or endothelial barrier injury. Collismycin C and pyrisulfoxin A, isolated from a red alga-associated marine actinomycete, reduced PM2.5-induced endothelial hyperpermeability, bronchoalveolar leukocyte recruitment, and IL-6 and TNF-α production, with evidence of ROS scavenging, p38 MAPK inhibition, and PI3K/Akt1 activation [34]. In epithelial and macrophage systems, fucosterol [26], *Sargassum horneri* extracts [54,55], *Sargassum swartzii* sulfated polysaccharides [78], the phlorotannin eckmaxol from *Ecklonia maxima* [42], and a *Prasiola japonica* fraction [79] attenuated particulate-matter-induced inflammatory and oxidative responses through modulation of Nrf2-related antioxidant defenses, NF-κB signaling, MAPK activation, and TLR-associated pathways.

Cigarette-smoke-related studies were fewer but addressed mechanisms relevant to COPD pathobiology. Apo-9′-fucoxanthinone protected bronchial epithelial cells from cigarette-smoke-extract-induced cytotoxicity by reducing mitochondrial ROS generation, apoptosis, and DNA damage [23]. Brevenal modulated inflammatory cytokine production and macrophage activation in LPS-stimulated respiratory cell systems [32]. In the only chronic in vivo exposure model within this domain, dietary astaxanthin reduced inflammatory-cell recruitment and emphysematous lung damage after 12 weeks of cigarette-smoke exposure, accompanied by activation of the Nrf2–ARE/HO-1 antioxidant pathway [22].

Overall, the pollution- and exposure-related evidence suggested a recurrent cytoprotective profile involving antioxidant activation, NF-κB/MAPK inhibition, TLR-pathway modulation, and protection of epithelial or endothelial integrity. However, the domain remained heterogeneous in exposure type, model duration, tested preparation, and outcome selection. Except for one chronic cigarette-smoke model, most studies used acute or short-term exposure systems, limiting inference regarding chronic disease modification in COPD or long-term pollution-related respiratory disease.

### 2.8. Risk of Bias

Risk of bias was assessed for all 65 unique included studies using the design-specific tools defined in Section 4.8. Overall, methodological limitations were common across the evidence base. Among the 44 studies with an in vivo component assessed with SYRCLE, none was rated as low risk of bias, 35 were rated as unclear risk, and 9 were rated as high risk. Among the 15 pure in vitro studies assessed with OHAT, none was rated as low risk, 13 were rated as unclear risk, and 2 were rated as high risk. Among the 5 randomized clinical trials assessed with RoB 2, 1 was rated as low risk and 4 as having some concerns. The single non-randomized clinical study assessed with ROBINS-I was rated as having a serious risk of bias.

Overall, 1 of 65 studies was rated as low risk of bias, 52 as unclear risk or having some concerns, and 12 as high or serious risk of bias. In the animal studies, the predominant limitation was insufficient reporting of procedures intended to reduce selection, performance, and detection bias, including sequence generation, allocation concealment, random housing, and blinding. Allocation concealment was generally rated as unclear when unreported and was not, by itself, considered sufficient to classify a study as high risk of bias. High-risk judgements were instead driven by confirmed threats to internal validity, including major or unexplained attrition, internal inconsistencies, missing ethics approval, inadequately characterized test materials, use of a non-validated disease model, or serious commercial conflicts of interest.

In the pure in vitro evidence base, unclear ratings mainly reflected incomplete reporting of blinding, selective-outcome-reporting safeguards, or conflicts of interest, whereas high-risk judgements were associated with inadequate characterization of the tested material or other major design limitations. Among the clinical studies, the randomized trials were generally limited by concerns related to the randomization process, deviations from intended interventions, or selective reporting. The uncontrolled before–after study was rated as having serious risk of bias, primarily because of confounding and the absence of a concurrent control group. Full domain-level judgements and study-specific justifications are reported in Supplementary Tables S2–S5.

### 2.9. Quantitative Synthesis

Quantitative synthesis was feasible only for selected preclinical model–outcome combinations. Overall, 17 preclinical studies contributed 60 comparisons across 11 outcomes, mainly from OVA-induced allergic airway disease and LPS-induced acute lung injury models. Clinical outcomes were not meta-analyzed because the clinical studies differed substantially in intervention, population, comparator, duration, and endpoint. Positive Hedges’ g values indicated a reduction in the pathological marker or injury-related outcome in the marine-derived intervention group relative to the corresponding disease-model control.

In OVA-induced allergic airway disease models, marine-derived interventions were associated with reductions in BALF eosinophils and type 2 inflammatory markers, including IL-4, IL-5, IL-13, and IgE-related outcomes. Pooled analyses showed reductions in BALF eosinophils (Hedges’ g 2.23, 95% CI 1.29–3.16), IL-4 (1.90, 0.89–2.90), IL-5 (2.18, 0.99–3.37), IL-13 (1.63, 0.82–2.43), OVA-specific IgE (1.46, 0.82–2.11), and total IgE (1.22, 0.21–2.23). The pooled effects were large and statistically significant across the selected outcomes, but heterogeneity was substantial for several estimates (I^2^ approximately 64–84%), and the number of contributing studies was small for some outcomes (e.g., total IgE, k = 3). These findings therefore indicated a consistent direction of effect across the pooled OVA model–outcome combinations rather than a precise estimate of the magnitude expected for any individual compound.

In LPS-induced acute lung injury models, pooled analyses showed reductions in TNF-α (Hedges’ g 4.45, 95% CI 2.25–6.65), IL-1β (3.62, 0.41–6.84), IL-6 (3.06, 0.98–5.15), myeloperoxidase activity (2.13, 1.31–2.96), and the lung wet-to-dry weight ratio (2.85, 1.87–3.83). Between-study heterogeneity was substantial for most outcomes, with I^2^ values of approximately 45–88% but was absent for myeloperoxidase activity (I^2^ = 0%) and lower for the wet-to-dry weight ratio (I^2^ = 44%). Across both disease-model clusters, the pooled estimates consistently favored the marine-derived interventions.

Post hoc stratified analyses indicated that the direction of effect was preserved across compound classes, whereas its magnitude was not (Supplementary Table S8). In the OVA cluster, all class-specific estimates favored the intervention, but significant between-class differences were detected for BALF eosinophils (*p* < 0.001), IL-13 (*p* = 0.023), IL-5 (*p* = 0.049), and OVA-specific IgE (*p* = 0.030). For BALF eosinophils, for example, pooled effects ranged from g = 1.25 (95% CI 0.35–2.14; k = 3) for polyphenol- or phlorotannin-rich preparations to g = 7.49 (4.74–10.25; k = 1) for echinochrome A, whereas the lipid extract Lyprinol showed a non-significant effect (g = 0.58, −0.49 to 1.65). In the LPS cluster, estimates for polysaccharides (IL-6: g = 2.25, 0.56–3.94; TNF-α: 3.13, 1.87–4.40; wet-to-dry ratio: 2.76, 0.73–4.80) and for C-phycocyanin (wet-to-dry ratio: 2.88, 0.62–5.13; myeloperoxidase: 2.43, 1.38–3.48) were directionally concordant, without significant between-class differences (*p* = 0.28–0.89), although the C-phycocyanin estimates for cytokines were highly heterogeneous and imprecise (k = 2). Stratification by preparation type did not reveal significant differences between isolated compounds or purified constituents and crude or multicomponent preparations for any testable outcome (*p* ≥ 0.13). In leave-one-out analyses, all pooled estimates retained a positive direction and statistical significance was preserved for 9 of 11 outcomes; IL-1β lost significance when either the C-phycocyanin study of Leung et al. [63] or the algal-polysaccharide study of Zhang et al. [62] was omitted, as did total IgE when either the fucoidan study of Maruyama et al. [46] or the more recent echinochrome study [28] was omitted. Excluding studies at high risk of bias did not materially change the estimates, except those for IL-1β (g = 4.30, −2.82 to 11.41; k = 2).

Small-study effects were explored using Egger’s regression test for outcomes with at least four contributing studies. Signals compatible with small-study effects were detected for several pooled outcomes; however, given the small number of studies within each meta-analysis, these results were considered exploratory and were not interpreted as definitive evidence of publication bias. The large, standardized effect sizes should likewise be interpreted cautiously because the pooled analyses combined structurally diverse compounds and preparations. Several outcomes showed substantial heterogeneity, and all contributing evidence was preclinical. Complete study-level data, pooled estimates, heterogeneity statistics, Egger-test results, and forest plots are reported in Supplementary Table S6 and Supplementary Figures S1–S11.

### 2.10. Certainty of Preclinical Meta-Analytic Evidence

The certainty of evidence was rated as very low for all 11 meta-analyzed outcomes. Downgrading reflected recurrent methodological limitations in the contributing studies, indirectness arising from the use of animal models and surrogate biomarker outcomes, imprecision related to the small number of studies contributing to several pooled estimates, inconsistency for outcomes with substantial between-study heterogeneity, and suspected publication bias or small-study effects where applicable.

Thus, although the pooled estimates were generally large, statistically significant, and consistent in direction, confidence in the magnitude and reproducibility of these effects was very limited. Quantitative synthesis supports the presence of a recurrent preclinical pharmacological signal across selected allergic-airway and acute-lung-injury models but does not provide confirmatory evidence of efficacy and cannot support direct clinical inference. Confirmation will require standardized and adequately characterized interventions, prospectively defined experimental protocols, appropriate randomization and blinding, transparent reporting of attrition and all prespecified outcomes, adequate sample sizes, and independent replication.

The outcome-level GRADE assessments and reasons for downgrading are reported in Supplementary Table S7. GRADE certainty was not formally assigned to the clinical evidence because the small number of studies and the heterogeneity of interventions, populations, and outcomes precluded pooled outcome-level assessment; the interpretability of the clinical findings should therefore be considered in conjunction with the RoB 2 and ROBINS-I assessments reported in Section 2.8.

## 3. Discussion

### 3.1. Principal Findings and Interpretation of the Evidence

This systematic review maps a broad but methodologically uneven body of evidence that points to biological activity of marine-derived natural compounds across experimental models of respiratory disease [7,8,9]. What emerges is a pattern rather than a proven treatment. No single compound class reached established efficacy in a defined respiratory condition; instead, structurally diverse marine-derived interventions were repeatedly reported to act on the same limited set of pathological processes that recur across respiratory diseases, i.e., inflammatory activation, oxidative stress, dysregulated immune responses, tissue injury, and remodeling.

This activity was documented across a wide range of marine sources and chemical classes, from sulfated polysaccharides [46,60], carotenoids [18,22], and polyphenols and phlorotannins [41,42] to lipid-derived products [44,51], peptides [71], and microbial metabolites [29,34]. Despite such chemical heterogeneity, the reported effects converged on a shared set of interconnected inflammatory, antioxidant, immune, and tissue-remodeling pathways rather than dispersing across unrelated mechanisms. Within the subset of preclinical studies comparable enough to be pooled, the direction of effect remained favorable across all 11 meta-analyzed outcomes (Section 2.9).

These observations are consistent with a recurrent preclinical pharmacological signal, yet they do not establish a general therapeutic effect of “marine-derived compounds” as a single intervention class. The evidence base remains dominated by small preclinical studies, heterogeneous preparations, surrogate outcomes, incompletely reported experimental procedures, and a limited number of clinical investigations. The central interpretation of this review is therefore one of biological convergence without therapeutic confirmation.

### 3.2. Mechanistic Convergence Versus Pharmacological Class Effect

One of the most consistent patterns across the included studies was the convergence of structurally different compounds on a relatively restricted network of biological processes. Across different experimental systems, marine-derived interventions repeatedly suppressed NF-κB- and MAPK-related inflammatory signaling, activated Nrf2/HO-1-dependent antioxidant responses, modulated type 2 and other immune pathways, attenuated inflammasome-related activity, and interfered with TGF-β/Smad-mediated remodeling (Section 2.3, Section 2.4, Section 2.5, Section 2.6 and Section 2.7). Each of these axes has an established role in respiratory pathobiology [80,81,82,83,84,85], which makes their recurrence across structurally unrelated marine compounds biologically coherent. These processes are, moreover, tightly interconnected: oxidative stress, innate immune activation, cytokine release, epithelial dysfunction, inflammatory-cell recruitment, and tissue remodeling frequently amplify one another [7,8,9]. Modulation of a single node may therefore propagate across several downstream outcomes as a plausible reading of the preclinical evidence rather than a causal mechanism confirmed across compounds. Mechanistic convergence, however, should not be interpreted as pharmacological equivalence. The interventions included in this review ranged from purified molecules with defined chemical structures to complex extracts, lipid mixtures, polysaccharide preparations, and incompletely characterized biological products. Compounds acting on the same signaling pathway can still differ markedly in potency, selectivity, bioavailability, metabolism, tissue distribution, toxicity, and manufacturability [81]. Part of the apparent convergence may also be an artefact of how the evidence was generated. NF-κB, for instance, is targeted by a broad range of dietary phytochemicals, including curcumin, resveratrol, quercetin, and epigallocatechin gallate, so its repeated inhibition is a common feature of natural-product pharmacology rather than a distinctive property of marine compounds [86]. Its recurrence may further reflect the predominance of LPS- and OVA-driven models in which this pathway is deliberately activated, rather than a mechanistic signature specific to marine sources.

Therefore, the evidence supports convergence at the level of biological pathways while leaving a class effect unproven. A productive next step would move beyond confirming that further marine products lower familiar inflammatory markers and instead identify which specific compounds, preparations, and molecular features produce reproducible effects under standardized experimental conditions.

### 3.3. Scope and Limits of the Quantitative Synthesis

The meta-analysis, comprising 17 preclinical studies contributing 60 comparisons across 11 outcomes, extends the narrative evidence in an important way. In OVA-induced allergic airway disease models, marine-derived interventions reduced BALF eosinophilia, IgE-related outcomes, and the type 2 cytokines IL-4, IL-5, and IL-13. In LPS-induced acute lung injury models, pooled effects favored the interventions for TNF-α, IL-1β, IL-6, myeloperoxidase activity, and pulmonary oedema measured by the lung wet-to-dry weight ratio. The consistency in direction across all 11 outcomes suggests that the recurrent anti-inflammatory signal observed narratively was not confined to isolated positive experiments.

The quantitative findings are best read as evidence of directional convergence. Standardized mean differences were often large, yet large effects in small preclinical studies are particularly vulnerable to methodological bias, selective reporting, model homogeneity, and small-study effects, as well documented in the in vivo literature [87], and compounded by incomplete reporting of experimental procedures [88]. Several pooled estimates also showed substantial between-study heterogeneity. Moreover, structurally different compounds and preparations were combined within shared model–outcome frameworks. This approach was appropriate for testing whether a broad cross-compound signal existed, but the resulting estimates should not be interpreted as the expected effect size of any individual intervention. The post hoc stratified analyses support this reading: the direction of effect was consistent across chemical classes, but its magnitude differed significantly between classes for several allergic-airway outcomes, and class-specific estimates frequently rested on one or two studies. The pooled estimates should therefore be regarded as a summary of directional consistency across heterogeneous interventions rather than as evidence that these compounds are interchangeable or share a common effect size. Compound-specific quantitative synthesis will require independent replication of the same, adequately characterized intervention in comparable models.

The meta-analysis therefore addresses a specific question: When different marine-derived interventions are tested in comparable preclinical respiratory models, do their effects converge in the same direction? Within the limits of the available evidence, the pooled results indicate directional consistency. Interchangeability of the interventions, reproducibility of the pooled effect magnitude, comparative superiority among compounds, and translation to humans all remain open questions, and treatment effects observed in animal experiments have frequently diverged from those subsequently measured in clinical trials [89]. The certainty assessment reinforces this reading. All 11 meta-analysed outcomes were rated as very low certainty because of recurrent risk-of-bias concerns, indirectness arising from animal models and surrogate outcomes [90], inconsistency or imprecision for several estimates, and suspected small-study effects where applicable. Statistical significance and effect magnitude are thus distinct dimensions from certainty of evidence: here, large pooled effects coexist with very low confidence in efficacy.

### 3.4. Why the Evidence Remains Translationally Immature

Translation depends less on whether biological activity is present than on whether it has been demonstrated with sufficient reliability, comparability, and translational relevance. On all three counts the current evidence falls short. Only 1 of 65 studies was rated at low risk of bias, against 52 rated unclear risk or some concerns and 12 rated high or serious risk. In animal studies, random sequence generation, allocation concealment, random housing, investigator blinding, and random outcome assessment were frequently unreported, a pattern consistent with long-standing deficiencies in the in vivo literature [87] and with persistent gaps in adherence to reporting standards [88]. High-risk judgements were additionally driven by unexplained attrition, internal inconsistencies, missing ethics approval, insufficient characterization of test materials, non-validated disease models, and serious commercial conflicts of interest.

Incomplete reporting is not proof that appropriate safeguards were absent. Yet when randomization, allocation procedures, blinding, attrition, or prespecified outcomes cannot be reconstructed from the published report, confidence in the estimated intervention effect is inevitably reduced, and this is a concern amplified in a literature characterized by small experimental groups and large reported effects.

Model dependence compounds this problem. The quantitative evidence was generated almost entirely from two highly standardized systems, OVA-induced allergic airway disease and LPS-induced acute lung injury, which are valuable for mechanistic and proof-of-concept research yet reproduce selected components of human disease rather than its full biological and clinical complexity [91]. OVA models predominantly capture experimentally induced type 2 inflammation, whereas severe human asthma encompasses type 2-low and mixed inflammatory endotypes that such models do not represent [92]. LPS models generate acute innate inflammatory responses that differ substantially from the clinical and biological heterogeneity of human ARDS [93]. The fibrosis evidence illustrates the same constraint in vitro, where anti-fibrotic activity was largely demonstrated against supraphysiological exogenous TGF-β1 stimulation, a reductionist system that does not reproduce the chronic, multicellular, self-amplifying remodeling of human pulmonary fibrosis [83,94]. Reproducibility within these systems therefore leaves the problem of indirectness unresolved.

Intervention identity poses further difficulty. Natural products are particularly sensitive to variation in species, geographical origin, harvesting conditions, extraction procedures, purification, molecular-weight distribution, formulation, and storage [95]. For complex extracts and polysaccharide-rich preparations, nominally similar interventions may therefore represent materially different exposures [96]. Without chemical characterization, batch standardization, and pharmacokinetic information, replication across laboratories and progression towards clinical development remain difficult.

Pharmacokinetics and pulmonary delivery represent a final, and arguably decisive, gap. Many of the compounds identified here (i.e., carotenoids, phycobiliproteins, and sulfated polysaccharides) were administered systemically in preclinical models, yet their oral bioavailability and lung-tissue exposure are frequently limited and rarely quantified [97,98]. The translational cost of this omission is illustrated by a small randomized, placebo-controlled crossover trial of sulforaphane, a widely studied Nrf2 activator, in which supplementation in healthy volunteers neither induced antioxidant gene expression nor protected against ozone-induced neutrophilic airway inflammation [99], despite the well-established role of this pathway in respiratory disease [81,82]. Demonstrable target engagement in the lung, rather than systemic administration alone, is therefore likely to be a prerequisite for clinical translation and a requirement that may depend on inhaled or otherwise optimized formulations [100].

### 3.5. From Biological Activity to Candidate Prioritization

The current evidence base reports biological activity for many compounds and preparations but provides limited basis for clearly prioritizing development candidates. Continued accumulation of isolated positive studies is unlikely, by itself, to advance the field, consistent with the broader disconnect documented between preclinical findings and clinical translation [89]. A more selective translational framework is required.

Candidate prioritization should integrate at least five dimensions: reproducibility across independent laboratories; adequate chemical characterization and batch standardization; evidence of activity across more than one complementary experimental system; plausible exposure at pharmacologically relevant concentrations; and an acceptable preliminary safety profile, in line with established principles for natural-product drug development [95]. Additional value should be assigned to compounds supported by mechanistic coherence across in vitro and in vivo studies, dose–response relationships, active comparators, and evidence that the observed effect is not restricted to a single surrogate biomarker.

Under such a framework, priority follows the completeness of the chain from compound identity to target engagement, biological effect, reproducibility, exposure, and safety. The size of the effect reported in a single experiment carries comparatively little weight in this ranking. This shift is particularly important for complex extracts. Such preparations may offer multi-target activity, but their development requires rigorous compositional profiling and identification of the constituents responsible for the reported biological effects. Purified compounds provide greater pharmacological precision but may lose potential synergistic effects present in the original biological matrix [101,102]. The appropriate development pathway may therefore differ by intervention type, but the requirement for reproducible identity and exposure is common to both.

### 3.6. Clinical Evidence and the Translational Gap

Clinical evidence remains insufficient to determine whether the preclinical signals identified in this review translate into meaningful respiratory benefit. The available human studies were few and heterogeneous with respect to compound, formulation, population, treatment duration, comparator, and outcome selection [36,37,38,44]. Most focused on asthma or allergic airway disease, and no sufficiently homogeneous clinical outcome could be pooled.

Some studies reported favorable changes in symptoms, pulmonary function, oxidative-stress markers, or immune outcomes, but these findings were not consistent across interventions. Recent clinical evidence also included neutral findings for clinically relevant outcomes [38], illustrating that strong biological plausibility and favorable preclinical results do not guarantee therapeutic efficacy.

Several features of the clinical evidence further limit its interpretability (Table 1). The interventions were nutritional supplements or lipid preparations rather than pharmaceutical-grade formulations, and the delivered doses of the putative active constituents were modest, approximately 72 mg EPA plus 48 mg DHA per day in the PCSO-524 trial and at least 120 mg EPA plus 180 mg DHA per day in the salmon-oil trial, with no link to pharmacokinetic or target-engagement data [37,38]. Favorable findings clustered in short-term studies using physiological, symptom-based, or biomarker endpoints such as hyperpnea-induced bronchoconstriction, exhaled mediators, or ex vivo cytokine production [36,37,59]; the one trial with an exacerbation-based primary endpoint was neutral [38]. Most studies lacked prospective registration, some relied on completer analyses or self-reported symptom scores, and product manufacturers were frequently involved in supply or funding [37,38,44]. All were conducted in adults with asthma or allergic rhinitis, so no clinical evidence exists for children or for COPD, pulmonary fibrosis, ALI/ARDS, or respiratory infections. Safety data were sparse and not systematically collected: no signal of harm emerged, but the available studies cannot exclude uncommon adverse events or interactions with standard inhaled therapy.

The gap between preclinical and clinical evidence reflects discontinuity across the translational pathway. Compounds tested clinically were not always those supported by the strongest or most reproducible preclinical evidence, preclinical dose–exposure relationships were rarely linked to human pharmacokinetics, and outcome selection often differed substantially between experimental and clinical studies. Future early-phase clinical development should therefore be preceded by explicit candidate selection and should incorporate pharmacokinetics, target engagement, safety, and mechanistically aligned biomarkers. Proof-of-concept trials should use standardized formulations and prospectively defined outcomes that can test whether the biological mechanisms suggested preclinically are also engaged in humans. Larger efficacy trials should follow only when this translational chain has been demonstrated.

### 3.7. Strengths and Limitations

This review has several strengths. It integrates in vitro, in vivo, and clinical evidence across the major respiratory applications investigated for marine-derived natural compounds while preserving the distinction between experimental and human evidence. The broad qualitative synthesis was complemented by quantitative pooling only when studies shared a sufficiently comparable disease model and outcome. Risk of bias was assessed using design-specific tools, and certainty was evaluated separately for each meta-analyzed preclinical outcome. This approach allowed statistical significance, methodological quality, and certainty of evidence to be interpreted as distinct dimensions.

Several limitations should also be acknowledged. First, the diversity of compounds, preparations, models, doses, routes of administration, and outcomes limited quantitative synthesis to a subset of the evidence base. Second, the meta-analysis combined structurally different interventions within common model–outcome frameworks. This was intended to evaluate cross-compound convergence and should not be interpreted as evidence of pharmacological interchangeability. Post hoc stratification by chemical class and preparation type partly addressed this issue, but it was not prespecified and was limited by the small number of studies per class. Third, the highest-dose eligible comparison was selected according to the prespecified analytical strategy. This avoided non-independence from multiple treatment arms but may preferentially represent maximal experimental efficacy rather than the most clinically relevant exposure.

Fourth, most pooled outcomes were based on small numbers of studies, several showed substantial heterogeneity, and small-study effects were suspected for multiple outcomes. Egger’s regression was only exploratory because the number of studies per meta-analysis was limited. Fifth, poor reporting frequently resulted in unclear risk-of-bias judgements; although this does not prove that appropriate methods were not used, it reduces confidence in the reported effects. Sixth, the restriction to English-language publications and the exclusion of grey literature may have increased susceptibility to publication bias. Finally, clinical heterogeneity precluded quantitative synthesis and formal outcome-level certainty assessment of the human evidence.

### 3.8. Research Priorities and Conclusions

The next phase of research should focus less on expanding the catalogue of marine compounds reported to have anti-inflammatory activity and more on establishing a reproducible translational pipeline. Preclinical studies should be prospectively planned, adequately powered, randomized, blinded where feasible, and reported in accordance with current experimental standards [88]. Intervention identity should be supported by chemical characterization, batch information, and, where relevant, pharmacokinetic or tissue-exposure data [95]. Replication by independent groups should become a prerequisite for candidate advancement.

Quantitative synthesis should also evolve from broad cross-compound comparisons towards compound-specific or chemically coherent meta-analyses as the evidence base matures. Harmonization of core outcomes within major respiratory models would substantially improve comparability and reduce selective reporting in future studies. For compounds progressing towards clinical investigation, preclinical models should be selected to test specific translational hypotheses rather than to reproduce additional demonstrations of generic anti-inflammatory activity. Sustainability and biodiversity considerations must also be integrated into the development framework for marine-derived therapeutics. The low natural abundance of many bioactive marine metabolites is a recognized constraint for pharmaceutical development [103], and wild harvest at the scale required would be environmentally unsustainable and risk depleting the biodiversity that constitutes the field’s core asset. Biotechnological solutions such as heterologous biosynthesis, marine aquaculture, and fermentation-based production of marine-derived compounds should be actively developed in parallel with pharmacological research to ensure scalable and ecologically responsible supply chains [103].

In conclusion, marine-derived natural compounds represent a chemically diverse source of compounds and preparations with reported biological activity on inflammatory, oxidative, immune, and tissue-injury pathways relevant to respiratory disease. The consistency of direction across the meta-analyzed preclinical outcomes is compatible with a recurrent cross-study pharmacological signal. However, this signal is accompanied by pervasive methodological uncertainty, substantial indirectness, heterogeneous interventions, suspected small-study effects, and very low certainty of evidence.

The current literature therefore supports cautious prioritization of selected candidates for rigorous evaluation, not a general claim of therapeutic efficacy for marine-derived compounds. Progress towards possible respiratory therapeutics will depend on moving from repeated demonstrations of biological activity to standardized compounds, reproducible experimental evidence, explicit candidate-selection criteria, and mechanistically continuous clinical development.

## 4. Methods

### 4.1. Search Strategy and Information Sources

The present systematic review was registered in PROSPERO (CRD420261396319) and conducted in accordance with the PRISMA 2020 guidelines [17]; the completed PRISMA 2020 checklist is provided as Supplementary Material. The review title was subsequently refined relative to the PROSPERO-registered protocol to more accurately reflect the final scope of the manuscript; the review question, eligibility criteria, and methodology remained unchanged. A comprehensive literature search was performed in PubMed/MEDLINE, Scopus, and Web of Science for studies published from January 2000 to December 2025. The search strategy combined controlled vocabulary, where available, and free-text terms related to marine-derived natural compounds and respiratory diseases or respiratory-relevant experimental models, including asthma, chronic obstructive pulmonary disease, acute lung injury, interstitial lung diseases, pulmonary fibrosis, respiratory infections, and pollution- or exposure-related lung injury. Only full-text articles published in English were considered eligible. The complete database-specific search strategies are reported in the Supplementary Material.

### 4.2. Eligibility Criteria

Eligibility criteria were defined a priori and are summarized in Table 2. Studies were eligible if they investigated natural compounds, extracts, fractions, or preparations directly derived from marine organisms, including macroalgae, microalgae or cyanobacteria, marine invertebrates, marine microorganisms, or marine-derived biological materials. For this review, “marine-derived natural compounds” was used as an operational umbrella term encompassing chemically defined molecules, enriched fractions, lipid extracts, polysaccharide preparations, and crude or whole-organism preparations, provided that the marine origin of the tested material was explicit. Consistent with this definition, semi-synthetic derivatives prepared directly from an isolated marine-derived natural product were considered eligible (one study [43]), whereas synthetic analogues without a direct marine-derived precursor were excluded.

Eligible study designs included original in vitro studies using respiratory-relevant cells or disease-relevant stimulated systems, original in vivo animal studies, randomized or non-randomized clinical intervention studies, and studies combining in vitro and in vivo experiments. Eligible models had to address respiratory diseases or airway/lung pathobiology, including allergic airway disease, asthma, acute lung injury or ARDS, pulmonary fibrosis or lung remodeling, respiratory infection, COPD-related injury, cigarette-smoke exposure, particulate-matter exposure, or other environmentally induced airway or lung injury models. Studies were required to report quantitative respiratory-relevant outcomes, including inflammatory, oxidative-stress, immune, remodeling, histological, lung-function, pathogen-related, or clinical symptom endpoints.

Studies were excluded if they evaluated synthetic derivatives without a direct marine-derived natural compound or preparation, non-respiratory disease models, purely in silico analyses, reviews, editorials, conference abstracts, case reports, case series, or antimicrobial studies that did not include a respiratory disease model or respiratory-relevant outcome. Articles for which the full text was unavailable were also excluded.

### 4.3. Study Selection

After removal of duplicate records, titles and abstracts were independently screened by two reviewers against the predefined eligibility criteria. Records considered potentially eligible by either reviewer underwent full-text assessment to confirm eligibility. Disagreements at either stage were resolved by discussion and consensus; when consensus could not be reached, a third reviewer was consulted. Reasons for exclusion at the full-text stage were recorded and are summarized in the PRISMA flow diagram.

### 4.4. Data Extraction

Data were independently extracted by two reviewers using a standardized extraction form. Extracted information included first author; year; country; study design; marine source organism or biological material; compound or preparation tested; chemical class, degree of characterization or standardization when reported; experimental or clinical mode; population or animal species; sample size; dose, route, timing and duration of administration; comparator or disease-model control; biological compartment; outcomes assessed; and main findings. For clinical studies, additional extracted data included participant characteristics, intervention duration, control condition, clinical endpoints, and safety information when available. For studies eligible for quantitative synthesis, numerical outcome data were extracted from text, tables, or figures when sufficient information was available to compute standardized mean differences. Studies were grouped by respiratory disease context and model type.

### 4.5. Study Classification and Evidence Mapping

Each included study was assigned to one primary design category to avoid double counting across evidence strata. The prespecified hierarchy for design classification was clinical human study > in vivo study > pure in vitro study. Thus, studies combining in vitro and in vivo experiments were classified as in vivo for the risk-of-bias partitioning and evidence mapping, while their mechanistic in vitro findings were retained in the narrative synthesis. Using this hierarchy, the 65 unique included studies comprised 44 studies with an in vivo component, 15 pure in vitro studies, 5 randomized clinical trials, and 1 non-randomized clinical study. Studies were then mapped according to respiratory disease context: asthma and allergic airway diseases, acute lung injury and ARDS, pulmonary fibrosis and lung remodeling, respiratory infections, and pollution- and exposure-related lung injury.

### 4.6. Narrative Synthesis

Given the expected heterogeneity in marine sources, compound classes, preparation methods, experimental models, doses, routes of administration, and reported outcomes, the evidence was first synthesized narratively. The synthesis was organized by respiratory disease context and by level of evidence, distinguishing pure in vitro studies, in vivo models, and clinical studies. Particular attention was given to recurrent mechanistic pathways, including inflammatory signaling, oxidative-stress regulation, immune modulation, epithelial or endothelial injury, and tissue remodeling. Mechanistic convergence across models was interpreted as pharmacological plausibility rather than evidence of clinical efficacy. Clinical studies were summarized separately because interventions, populations, and endpoints were too heterogeneous for quantitative pooling.

### 4.7. Quantitative Synthesis (Meta-Analysis)

Quantitative synthesis was restricted to preclinical model–outcome combinations for which at least three independent studies reported sufficiently comparable outcomes. Clinical studies were not meta-analyzed because of the small number of trials and the non-overlapping interventions, populations, comparators, and endpoints. For each eligible comparison, the standardized mean difference was calculated as Hedges’ g (bias-corrected) [104] comparing the marine-derived compound group with the corresponding disease-model control group. To harmonize interpretation across outcomes, effect sizes were coded so that a positive Hedges’ g indicated a reduction of the pathological marker or injury-related outcome by the marine-derived intervention relative to the disease-model control. When multiple doses were reported within the same study, the highest-dose group was selected for the primary analysis to avoid including multiple correlated comparisons from the same experiment and to capture the maximal pharmacological signal. When studies reported outcomes in multiple biological compartments, data were extracted from the compartment most consistently reported across studies within the same model–outcome cluster. Random-effects meta-analyses were performed using the Der Simonian-Laird estimator [105]. Between-study heterogeneity was assessed using Cochran’s Q, I^2^, and τ^2^. Small-study effects were explored using Egger’s regression test [106] when at least four studies contributed to a given pooled estimate. Because the number of studies per outcome was small, Egger’s test was interpreted as an exploratory signal rather than definitive evidence of publication bias. Dispersion measures reported as standard error of the mean were converted to standard deviations. Studies that did not report, or allow derivation of, an appropriate measure of dispersion were excluded from quantitative synthesis. Additional post hoc analyses, not prespecified in the registered protocol, were performed to examine whether pooled estimates were driven by particular types of intervention. Each pooled outcome was stratified by the chemical class of the tested intervention (polysaccharides; polyphenol- or phlorotannin-rich preparations; carotenoids; phycobiliproteins; naphthoquinone pigments; marine lipid extracts; other crude seaweed extracts) and, separately, by preparation type (isolated compound or purified constituent versus crude or multicomponent preparation). Random-effects estimates were computed within each subgroup, and between-subgroup differences were assessed with the Q-test for subgroup differences. The robustness of the pooled estimates was further explored with leave-one-out analyses and by excluding studies rated at high risk of bias. Compound-specific meta-analyses were not feasible, as no single compound or preparation had been evaluated in at least three independent studies for the same model–outcome combination; where two studies were available, within-compound estimates were reported descriptively. Per-study data, pooled estimates, and forest plots are reported in Supplementary Table S6 and Supplementary Figures S1–S11. The post hoc analyses are reported in Supplementary Table S8. Statistical analyses were performed using Python 3.11 (Python Software Foundation, Wilmington, DE, USA).

### 4.8. Risk-of-Bias Assessment

Risk of bias was assessed independently by two reviewers using tools appropriate to the study design. Randomized clinical trials were assessed with the revised Cochrane Risk of Bias tool for randomized trials (RoB 2) [107]. The single non-randomized clinical intervention study was assessed with ROBINS-I [108], because its uncontrolled before–after design could not be appropriately evaluated using RoB 2. Studies with an in vivo component were assessed with the SYRCLE risk-of-bias tool [109], and pure in vitro studies were assessed with the OHAT/NTP risk-of-bias tool [110].

To avoid double counting, risk-of-bias tool assignment followed the prespecified study-classification hierarchy: clinical human studies first, then in vivo animal studies, and finally pure in vitro studies. Accordingly, 5 randomized clinical trials were assessed using RoB 2, 1 non-randomized clinical study with ROBINS-I, 44 studies with an in vivo component using SYRCLE, and 15 pure in vitro studies with OHAT. For studies combining in vitro and in vivo experiments, tool assignment was based on the in vivo component, while relevant in vitro mechanistic findings were retained in the narrative synthesis.

RoB 2 and ROBINS-I overall judgements followed the standard algorithms for each tool. Because SYRCLE and OHAT do not prescribe a single fixed algorithm for deriving overall judgements, explicit decision rules were prespecified. For SYRCLE, a study was rated as high risk of bias if any domain critical to internal validity was judged high risk, including known baseline imbalance, detection bias for a subjective primary outcome, major or unexplained attrition, missing ethics approval, major internal inconsistency, non-validated disease model, or serious undisclosed commercial conflict of interest. Allocation concealment rated as unclear when unreported was not, by itself, considered sufficient to classify a study as high risk. For OHAT, studies were rated as high risk when the test material was inadequately characterized, when the design was confounded, or when a material commercial conflict of interest directly affected interpretation. Studies not meeting criteria for low or high risk were rated as unclear or some concerns, depending on the tool. Disagreements were resolved by consensus and, when necessary, by a third reviewer. Full domain-level judgements are reported in Supplementary Tables S2–S5.

### 4.9. Certainty of Evidence

The certainty of the quantitative preclinical evidence was assessed for each meta-analyzed outcome using a GRADE approach adapted for preclinical evidence synthesis. Certainty was downgraded for risk of bias, inconsistency, indirectness, imprecision, and suspected publication bias. Indirectness was considered inherent when evidence came from animal models or surrogate biomarker outcomes rather than clinical respiratory endpoints. Imprecision was considered when the number of contributing studies was small, or confidence intervals were wide. Suspected publication bias was informed by small-study effects and by the broader predominance of small, single-laboratory preclinical studies.

GRADE certainty was applied only to outcomes included in the preclinical meta-analysis. Clinical studies were not assigned pooled GRADE certainty ratings because of the limited number of studies, heterogeneity of interventions and outcome measures, and the absence of outcome-level pooling. The certainty and interpretability of clinical findings were therefore considered in relation to the corresponding RoB 2 or ROBINS-I judgements.

## Figures and Tables

**Table 1:** Characteristics, main results, and risk of bias of the clinical studies included in the review.

Study	Design and Population	Sample Size	Intervention (Daily Dose) and Comparator	Duration	Outcomes Assessed	Main Results	Overall Risk of Bias
Emelyanov 2002 [36]	Double-blind, placebo-controlled, parallel-group RCT; adults with steroid-naïve atopic asthma	46 randomized (23/23); all completed	Lipid extract of *Perna canaliculus* (Lyprinol), 2 capsules b.i.d. (each 50 mg n − 3 PUFA + 100 mg olive oil) vs. olive-oil placebo	8 weeks	Daytime wheeze, morning PEF, exhaled H_2_O_2_, β_2_-agonist use, FEV1	Reduced daytime wheeze (*p* = 0.026) and exhaled H_2_O_2_ (*p* = 0.022); increased morning PEF (+47.0 vs. −33.4 L/min); reduced β_2_-agonist use; FEV1 unchanged; no significant side effects	Some concerns (RoB 2)
Mickleborough 2013 [37]	Double-blind, placebo-controlled, randomized crossover (2-week washout); adults with asthma and hyperpnea-induced bronchoconstriction	20	PCSO-524 (*P. canaliculus* lipid fraction), 8 capsules/day (~72 mg EPA + 48 mg DHA) vs. placebo	3 weeks per period	Maximum post-EVH fall in FEV1; EBC cysteinyl leukotrienes and 8-isoprostane; urinary mediators; FeNO; symptoms; rescue medication	Maximum post-EVH FEV1 fall −8.4% vs. −22.5% with placebo (*p* < 0.05); reduced EBC cysteinyl leukotrienes and 8-isoprostane; improved symptoms and reduced rescue-medication use	Low (RoB 2)
Mølsæter 2025 [38]	Double-blind, placebo-controlled, multicentre exploratory RCT; adults with type 2 (eosinophilic) asthma	70 randomized; 66 analyzed (33/33)	Unrefined salmon oil (*Salmo salar*; OmeGO), 6 g/day (≥120 mg EPA, ≥180 mg DHA) vs. MCT-oil placebo	20 weeks	Composite of moderate/severe exacerbations (primary); ACQ-5; FEV1; FeNO; adverse events	No effect on the primary outcome (median time to first event 37 vs. 15 days, *p* = 0.347; ≥1 event 73% vs. 82%); no differences in ACQ-5, FEV1, or FeNO; adverse events similar between groups	Some concerns (RoB 2)
Denisenko 2023 [44]	Open-label, uncontrolled before–after study; obese adults with mild asthma on budesonide/formoterol (healthy volunteers as untreated reference)	19 patients (+16 healthy volunteers)	1-*O*-alkylglycerols from the squid *Berryteuthis magister*, 0.4 g/day (0.2 g b.i.d.) added to usual therapy; no concurrent control	3 months	ACQ; spirometry (FEV1, FEV1/VC); plasma fatty acids and plasmalogens; cytokines; adipokines; oxylipins	Increased FEV1 and FEV1/VC; ACQ improved in 17/19 patients; reduced TNF-α, IL-4, IL-17a, TXB_2_, and LTB_4_; increased n − 3 PUFA and plasmalogens	Serious (ROBINS-I)
Cingi 2008 [58]	Double-blind, placebo-controlled, parallel-group RCT; adults with allergic rhinitis	150 randomized; 129 completed (85/44)	*Spirulina platensis*, 2 g/day vs. placebo	6 months	Nasal symptoms (discharge, sneezing, congestion, itching); physical findings; patient-rated satisfaction and effectiveness	Improvement in all symptoms and physical findings vs. placebo (*p* < 0.001)	Some concerns (RoB 2)
Mao 2005 [59]	Double-blind, placebo-controlled, randomized crossover; adults with allergic rhinitis	36 enrolled	Spirulina-based supplement, 1 g/day or 2 g/day vs. placebo	12 weeks	IL-4, IFN-γ, and IL-2 production by PHA-stimulated PBMCs (no clinical endpoints)	IL-4 reduced by 32% with 2 g/day (*p* = 0.0082); no effect with 1 g/day; IFN-γ and IL-2 unchanged	Some concerns (RoB 2)

**Table 2:** Eligibility criteria.

Domain	Criteria
Study characteristics	Original in vitro studies with disease-relevant cell lines or primary cultures under stimulated conditions, e.g., LPS, OVA, TGF-β, or cigarette smoke exposure; original in vivo studies; clinical studies; respiratory diseases or airway/lung models involving upper or lower respiratory tract pathology; quantitative data required.
Intervention	Natural compounds directly derived from marine organisms (e.g., macroalgae, microalgae, marine invertebrates, marine microorganisms); extracts or purified marine-derived molecules.
Outcomes	Respiratory-related endpoints including inflammation, oxidative stress, immune modulation, lung injury or remodeling, histopathology, pulmonary function, or clinical symptom measures.
Exclusions	Synthetic derivatives not directly marine-derived; non-respiratory studies; reviews/editorials/conference abstracts; case reports and case series; exclusively in silico or antimicrobial studies without respiratory models; unavailable full text.

## Data Availability

The original contributions presented in this study are included in the article/Supplementary Material. Further inquiries can be directed to the corresponding author.

## Supplementary Materials

The following supporting information can be downloaded at https://www.mdpi.com/article/10.3390/md24100358/s1: Supplementary Methods and Overview (database-specific search strategies and risk-of-bias methods); PRISMA 2020 Checklist; Table S1: Characteristics of included studies, by disease category, with Tables S1A–S1E (asthma and allergic airway diseases; acute lung injury and ARDS; pulmonary fibrosis and lung remodeling; respiratory infections; pollution- and exposure-related lung injury); Table S2: SYRCLE risk of bias, in vivo animal studies; Table S3: OHAT risk of bias, in vitro studies; Table S4: Cochrane RoB 2, randomised controlled trials; Table S5: ROBINS-I, non-randomised clinical study; Table S6: Pooled random-effects estimates; Table S7: GRADE certainty of evidence (Summary of Findings); Table S8: Post hoc analyses, with Table S8A (pooled preclinical outcomes stratified by chemical class and by preparation type) and Table S8B (leave-one-out and risk-of-bias sensitivity analyses); Figures S1–S11: Forest plots of the pooled preclinical outcomes (S1–S5, acute lung injury, LPS model: IL-1β, IL-6, lung wet/dry ratio, MPO, TNF-α; S6–S11, asthma, OVA model: BALF eosinophils, IL-13, IL-4, IL-5, OVA-specific IgE, total IgE). References [18,19,20,21,22,23,24,25,26,27,28,29,30,31,32,33,34,35,36,37,38,39,40,41,42,43,44,45,46,47,48,49,50,51,52,53,54,55,56,57,58,59,60,61,62,63,64,65,66,67,68,69,70,71,72,73,74,75,76,77,78,79,111,112,113] are cited in the Supplementary Materials.
