# Supplementary material for: Marine-Derived Natural Compounds in Respiratory Diseases: A Systematic Review of Preclinical Mechanisms, Clinical Evidence, and Translational Gaps
Source: Mar Drugs. 2026 Oct 13;24(10):358. doi: 10.3390/md24100358 (PMC13706628; doi:10.3390/md24100358)
Supplement: Supplementary file 1 [file marinedrugs-24-00358-s001.zip › marinedrugs-4565772-supplementary.pdf]

Supplementary Material

Marine-derived natural compounds in respiratory diseases: a systematic review of preclinical mechanisms, clinical evidence, and translational gaps

Supplementary Methods and Overview

This document provides the full-text-based supporting tables for the systematic review: the characteristics of all included studies (Table S1, organized by respiratory-disease category) and the methodological risk-of-bias (RoB) assessments (Tables S2–S5). All data were extracted and all RoB judgements were made from the complete full texts of the included articles (not from abstracts).

Search strategy. Full database-specific search strings used for the literature search described in Section 4.1 of the main manuscript are reported below. Searches were restricted to English-language, full-text articles; no additional filters were applied.

PubMed/MEDLINE. ("marine organism*"[Title/Abstract] OR "marine natural product*"[Title/Abstract] OR "seaweed"[Title/Abstract] OR "algae"[Title/Abstract] OR "marine sponge*"[Title/Abstract] OR "marine bacteria"[Title/Abstract] OR "marine cyanobacteria"[Title/Abstract] OR "marine actinomycete"[Title/Abstract] OR "marine fungi"[Title/Abstract] OR "soft coral"[Title/Abstract] OR "gorgonian"[Title/Abstract] OR "tunicate"[Title/Abstract] OR "ascidian"[Title/Abstract] OR "marine mollusc"[Title/Abstract] OR "bivalve"[Title/Abstract] OR "gastropod"[Title/Abstract] OR "sea cucumber"[Title/Abstract] OR "sea urchin"[Title/Abstract] OR "Marine Drugs"[Journal])
AND
("lung disease*"[Title/Abstract] OR "respiratory disease*"[Title/Abstract] OR "chronic obstructive pulmonary disease"[Title/Abstract] OR "COPD"[Title/Abstract] OR "asthma"[Title/Abstract] OR "rhinitis"[Title/Abstract] OR "respiratory allerg*"[Title/Abstract] OR "pulmonary fibrosis"[Title/Abstract] OR "lung inflammation"[Title/Abstract] OR "bronchitis"[Title/Abstract] OR "respiratory tract infection*"[Title/Abstract] OR "lung injury"[Title/Abstract])
AND
("inflammation"[Title/Abstract] OR "oxidative stress"[Title/Abstract] OR "antioxidant"[Title/Abstract] OR "anti-inflammatory"[Title/Abstract] OR "immunomodulat*"[Title/Abstract] OR "redox"[Title/Abstract])

Scopus. (TITLE-ABS-KEY("marine organism*" OR "marine animal*" OR "marine compound*" OR "marine molecule*" OR "marine natural product*" OR "marine extract*" OR "marine-derived" OR "seaweed" OR "algae" OR "marine sponge*" OR "marine bacteria" OR "marine microalgae" OR "marine secondary metabolite*" OR "marine bioactive compound*" OR "holothuria" OR "sea cucumber" OR echinoderms OR "sea urchin*"))
AND
(TITLE-ABS-KEY("lung disease*" OR "respiratory disease*" OR "chronic obstructive pulmonary disease" OR "COPD" OR "asthma" OR "rhinitis" OR "respiratory allerg*" OR "pulmonary fibrosis" OR "lung inflammation" OR "bronchitis" OR "respiratory tract infection*" OR "lung injury"))
AND
(TITLE-ABS-KEY("inflammation" OR "oxidative stress" OR "antioxidant" OR "anti-inflammatory" OR "immunomodulat*" OR "redox"))

Web of Science. TS=(("marine organism*" OR "marine animal*" OR "marine compound*" OR "marine molecule*" OR "marine natural product*" OR "marine extract*" OR "marine-derived" OR "seaweed" OR "algae" OR "marine sponge*" OR "marine bacteria" OR "marine microalgae" OR "marine secondary metabolite*" OR "marine bioactive compound*" OR holothuria OR "marine sponge*" OR "sea cucumber" OR echinoderms OR "sea urchin*"))
AND
TS=(("lung disease*" OR "respiratory disease*" OR "chronic obstructive pulmonary disease" OR "COPD" OR "asthma" OR "rhinitis" OR "respiratory allerg*" OR "pulmonary fibrosis" OR "lung inflammation" OR "bronchitis" OR "respiratory tract infection*" OR "lung injury"))
AND
TS=(("inflammation" OR "oxidative stress" OR "antioxidant" OR "anti-inflammatory" OR "immunomodulat*" OR "redox"))

Design classification and RoB-tool assignment. Each study was assigned a single primary design for RoB partitioning using the precedence clinical (human) > *in vivo* (any animal component, including combined *in vitro*+*in vivo*) > *in vitro* (no animal/human component). This yields 44 in vivo studies (assessed with SYRCLE), 15 pure in-vitro studies (assessed with the OHAT tool), 5 randomized controlled trials (assessed with Cochrane RoB 2) and 1 non-randomized clinical study (assessed with ROBINS-I).

Twenty-one of the 44 in-vivo studies combined *in-vitro* and *in-vivo* experiments and are classified here as *in-vivo*, consistent with the hierarchy above. Every one of the 65 unique included studies is assessed for risk of bias; none is left unassessed.

Risk-of-bias tools. SYRCLE [21] for in-vivo animal studies; the OHAT/NTP tool [22] for *in vitro* studies; the revised Cochrane Risk-of-Bias tool RoB 2 [20] for RCTs; and ROBINS-I [24] for the non-randomized study. Each domain was rated Low / Unclear (Some concerns) / High, or, for ROBINS-I, Low / Moderate / Serious / Critical.

Overall-judgement rules. RoB 2 and ROBINS-I follow their standard published algorithms. Because SYRCLE and OHAT have no fixed algorithm for an overall verdict, the following explicit rules were applied:

- SYRCLE - HIGH if any domain critical to internal validity is High (a known baseline imbalance; detection bias on a subjective primary outcome; major/unexplained attrition; or a serious 'other' flaw such as an undisclosed commercial conflict of interest in a study testing the sponsor's own product, a non-validated disease model, a major internal inconsistency, or missing ethics approval). LOW if sequence generation is Low, no domain is High, and detection/attrition/reporting are adequate. UNCLEAR otherwise. Allocation concealment being Unclear (the norm for animal work) does not by itself raise a study to High.
- OHAT - HIGH if exposure characterization is High (uncharacterized test material), if there is a commercial conflict of interest, or if the design is confounded. LOW if selection, confounding, exposure, outcome, attrition and 'other' domains are all Low. UNCLEAR otherwise (typically driven by unreported blinding and absent pre-registration, which are normative for single-laboratory in-vitro pharmacology).

PRISMA 2020 Checklist

Completed PRISMA 2020 checklist mapping each reporting item to the section, page or table of the manuscript (and this Supplementary Material) where it is addressed.

Section	Item #	Checklist item	Location where item is reported
**TITLE**			
Title	1	Identify the report as a systematic review.	Title
**ABSTRACT**			
Abstract	2	See the PRISMA 2020 for Abstracts checklist.	Abstract
**INTRODUCTION**			
Rationale	3	Describe the rationale for the review in the context of existing knowledge.	Section 1 (Introduction), paragraphs 3-4
Objectives	4	Provide an explicit statement of the objective(s) or question(s) the review addresses.	Section 1 (Introduction), final paragraph
**METHODS**			
Eligibility criteria	5	Specify the inclusion and exclusion criteria for the review and how studies were grouped for the syntheses.	Section 4.2 (Eligibility criteria); Table 2
Information sources	6	Specify all databases, registers, websites, organisations, reference lists and other sources searched or consulted, and the date each source was last searched.	Section 4.1 (Search strategy and information sources)
Search strategy	7	Present the full search strategies for all databases, registers and websites, including filters and limits used.	Section 4.1; full database-specific strings in Supplementary Material
Selection process	8	Specify the methods used to decide whether a study met the inclusion criteria, including how many reviewers screened each record/report, whether independently, and automation tools used.	Section 4.3 (Study selection)
Data collection process	9	Specify the methods used to collect data, including how many reviewers collected data, whether independently, and how data were obtained from study investigators.	Section 4.4 (Data extraction)
Data items	10a	List and define all outcomes for which data were sought.	Section 4.4 (Data extraction); Section 4.6 (Narrative synthesis)
Data items	10b	List and define all other variables for which data were sought.	Section 4.4 (Data extraction); Section 4.5 (Study classification and evidence mapping)
Study risk of bias assessment	11	Specify the methods used to assess risk of bias, including how many reviewers assessed each study and whether independently, and tools used.	Section 4.8 (Risk of bias assessment)
Effect measures	12	Specify for each outcome the effect measure(s) used in the synthesis or presentation of results.	Section 4.7 (Quantitative synthesis (meta-analysis))
Synthesis methods	13a	Describe the processes used to decide which studies were eligible for each synthesis.	Section 4.6; Section 4.7
Synthesis methods	13b	Describe any methods required to prepare the data for presentation or synthesis.	Section 4.6; Section 4.7
Synthesis methods	13c	Describe methods used to tabulate or visually display results of individual studies and syntheses.	Section 4.6 (Narrative synthesis)
Synthesis methods	13d	Describe methods used to synthesise results and provide rationale (e.g. meta-analysis).	Section 4.7 (Quantitative synthesis (meta-analysis))
Synthesis methods	13e	Describe methods used to explore possible causes of heterogeneity among study results.	Section 4.7
Synthesis methods	13f	Describe sensitivity analyses conducted to assess robustness of synthesised results.	Section 4.7
Reporting bias assessment	14	Describe methods used to assess risk of bias due to missing results in a synthesis (arising from reporting biases).	Section 4.7 (small-study effects / Egger test)
Certainty assessment	15	Describe methods used to assess certainty in the body of evidence for an outcome.	Section 4.9 (Certainty of evidence)
**RESULTS**			
Study selection	16a	Describe results of the search and selection process, from the number of records identified to the number of studies included, ideally using a flow diagram.	Section 2.1 (Study selection)

Study selection	16b	Cite studies that might appear to meet the inclusion criteria but were excluded, and explain why.	Section 2.1
Study characteristics	17	Cite each included study and present its characteristics.	Section 2.2 (Study characteristics and evidence map); Table S1A-E
Risk of bias in studies	18	Present assessments of risk of bias for each included study.	Section 2.8 (Risk of bias); Tables S2-S5
Results of individual studies	19	For all outcomes, present a summary of results for each study.	Sections 2.3-2.7 (disease-domain narrative results)
Results of syntheses	20a	Briefly summarise the characteristics and risk of bias among studies contributing to each synthesis.	Section 2.9 (Quantitative synthesis)
Results of syntheses	20b	Present results of all statistical syntheses, including effect estimates, confidence intervals, and heterogeneity measures.	Section 2.9; Table S6
Results of syntheses	20c	Present results of investigations of possible causes of heterogeneity.	Section 2.9
Results of syntheses	20d	Present results of sensitivity analyses.	Section 2.9
Reporting biases	21	Present assessments of risk of bias due to missing results for each synthesis.	Section 2.9 (Egger regression test)
Certainty of evidence	22	Present assessments of certainty in the body of evidence for each outcome.	Section 2.10 (Certainty of preclinical meta-analytic evidence); Table S7
**DISCUSSION**			
Discussion	23a	Provide a general interpretation of the results in the context of other evidence.	Sections 3.1-3.2
Discussion	23b	Discuss limitations of the evidence included in the review.	Section 3.7 (Strengths and limitations)
Discussion	23c	Discuss limitations of the review processes used.	Section 3.7 (Strengths and limitations)
Discussion	23d	Discuss implications of the results for practice, policy and future research.	Sections 3.5, 3.6, 3.8
**OTHER INFORMATION**			
Registration and protocol	24a	Provide registration information for the review, including register name and registration number.	PROSPERO CRD420261396319; Abstract (Methods); Section 4.1
Registration and protocol	24b	Indicate where the review protocol can be accessed, or state that a protocol was not prepared.	PROSPERO record CRD420261396319
Registration and protocol	24c	Describe and explain any amendments to information provided at registration or in the protocol.	Section 4.1 (Methods), lines 120-124: title refined relative to PROSPERO-registered protocol; review question, eligibility criteria, and methodology unchanged
Support	25	Describe sources of financial or non-financial support for the review, and the role of funders.	Back matter - Funding
Competing interests	26	Declare any competing interests of review authors.	Back matter - Conflicts of Interest / Declaration of competing interest
Availability of data, code and other materials	27	Report which of the following are publicly available: template data collection forms, data extracted from included studies, analytic code, other materials.	Back matter - Data Availability Statement

Table S1. Characteristics of included studies, by disease category

Studies are ordered alphabetically by first author within each category. NR = not reported. Design: *in vitro* / *in vivo* / *in vitro* + *in vivo* / clinical.

Table S1A - Asthma and allergic airway diseases (n = 28)

Study (Author Year)	Country	Design	Marine source (species)	Compound (class)	Model system	Dose / route / duration	Key outcomes & main findings	Main mechanism / pathway
Abdelmawgood 2023 [27]	Egypt	*In vitro* + *in vivo*	Sea urchin (*Paracentrotus lividus*)	Echinochrome A (polyhydroxylated naphthoquinone pigment)	OVA asthma in 32 female BALB/c mice; cell-free DPPH/RBC-hemolysis assays + Keap1 docking	0.1 & 1 mg/kg i.p., 1 h before each challenge (days 21–23); *in vitro* DPPH 20–100 μg/mL, hemolysis 50–300 μg	Ech 1 mg/kg dose-dependently lowered serum IgE, IL-4, IL-1β, MDA/NO and raised GST/CAT/SOD; reduced nasal scratching and improved histopathology (docking −7.9 kcal/mol).	Keap1/Nrf2 activation; antioxidant/anti-inflammatory
Abdelmawgood 2024 [28]	Egypt	*In vivo*	Sea urchin (species not named)	Echinochrome (polyhydroxylated naphthoquinone pigment)	OVA allergic asthma in 32 female BALB/c mice	0.1 & 1 mg/kg i.p. daily for 7 days from day 17 (1 h before challenge)	Dose-dependently reduced nasal scratching, BALF eosinophils, serum IgE (670.6→430.6 pg/mL), IL-4/IL-13, MDA/NO; raised GSH/CAT/GST; cut %CD3+ cells and p-NF-κB p65.	NF-κB p65 suppression; Th2-cytokine, IgE and oxidative-stress attenuation
Abu Bakar 2015 [50]	Malaysia	*In vivo*	Red seaweed (*Eucheuma cottonii*)	*E. cottonii* extract SECE (lectins/peptides + polyphenols)	OVA allergic asthma in female Sprague-Dawley rats (n=6/group); SRBC DTH study	150 & 300 mg/kg oral once daily, weeks 6–10; comparator loratadine 10 mg/kg	Dose-dependently reduced lung eosinophilia/inflammation and bronchiole smooth-muscle thickness; downregulated IL-4, TNF-α, NF-κB, EGFR, MMP-9 and upregulated IFN-γ; 300 mg/kg often outperformed loratadine.	NF-κB suppression (TNF-α/EGFR/MMP-9); Th2→Th1 shift
Cingi 2008 [58]	Turkey	Clinical RCT	Cyanobacterium (*Spirulina platensis*)	*Spirulina whole-algae*supplement (contains phycocyanin)	150 allergic rhinitis patients (19–49 yr); double-blind placebo-controlled (129 completed)	2,000 mg/day oral for 6 months	Significantly improved all four symptoms and physical findings vs placebo (P<0.001); satisfaction 7.21 vs 3.40 and effectiveness 7.44 vs 3.54 (both P<0.001).	Not directly tested; proposed anti-inflammatory (prior IL-4 reduction)
Coulson and O'Donnell 2000 [25]	Australia	*In vivo*	Marine sponge (*Petrosia contignata*)	Contignasterol IZP-94,005 (highly oxygenated steroid)	OVA-sensitized guinea-pigs; allergen-induced plasma-exudation model	0.2–200 mg/kg i.t. superfused 30 or 5 min before OVA; comparators budesonide & nedocromil 200 mg/kg i.t.	At 30 min pre-OVA, 100 & 200 mg/kg significantly inhibited exudation (p≤0.05), comparable to budesonide/nedocromil; at 5 min pre-OVA only nedocromil worked.	Inhibition of allergen-induced microvascular leak; proposed mast-cell mediator-release inhibition (non-GR)
Denisenko 2023 [44]	Russia	Clinical (non-randomized)	Squid (*Berryteuthis magister*)	1-O-alkyl-glycerols / alkylglycerols (ether lipids; supplement Incodamarine)	Open-label non-randomized trial; 19 obese mild-asthma patients + 16 healthy controls	0.4 g/day pure AGs oral (0.2 g BID) for 3 months, on top of budesonide/formoterol	FEV1 rose (105.2→109.0, p<0.001); clinically significant ACQ improvement in 17/19 by 3 months; n-3 PUFA up, arachidonic acid/LTB4/TXB2 down; adiponectin up, leptin down; several cytokines reduced.	AGs as plasmalogen precursors; reduced 5-LOX/COX eicosanoids; NF-κB/MAPK inhibition; leptin/adiponectin rebalance
Emelyanov 2002 [36]	Russia	Clinical RCT	NZ green-lipped mussel (*Perna canaliculus*)	Lipid extract Lyprinol (omega-3 PUFA EPA+DHA + carotenoids)	46 adults with mild-to-moderate steroid-naive atopic asthma; double-blind RCT (23 vs 23)	2 caps (50 mg omega-3 + 100 mg olive oil each) BID for 8 weeks; placebo 150 mg olive oil	Significantly reduced daytime wheeze (p=0.026), increased morning PEF (+47.0 vs −33.4 L/min, p=0.00001), reduced exhaled H2O2 (p=0.022) and β2-agonist use; no change in FEV1.	Omega-3 PUFA inhibition of 5-LOX and COX; antioxidant carotenoid action
Herath 2020 [112]	Republic of Korea	*In vivo*	Brown alga (*Sargassum horneri*)	*S. horneri* ethanol extract SHE; active mojabanchromanol (chromene)	OVA-sensitization + PM-challenge in female BALB/c mice (n=4–8, 7 groups)	200 & 400 mg/kg oral daily before PM exposure; prednisone 5 mg/kg control	Dose-dependently reduced AHR, eosinophil infiltration, mast-cell degranulation and serum IgE; SHE 400 lowered DC population, GATA-3/RORγT, IL-4/IL-13/IL-17a and STAT3/STAT5 phosphorylation; often matched prednisone.	Dual Th2 (GATA3/STAT5) and Th17 (RORγT/STAT3) suppression; reduced DC/mast-cell activation
Kim J 2023 [41]	Republic of Korea	*In vivo*	Brown seaweed (*Agarum cribrosum*)	*A. cribrosum* 70% acetone extract + isolated phlorotannin Trifuhalol A	OVA inhalation-induced allergic asthma in female BALB/c mice (6 groups, N=6)	AC 100 mg/kg and trifuhalol A 10/20 mg/kg oral daily from day 1; dexamethasone 1.5 mg/kg i.p. control	Reduced bronchial epithelium thickening (35.1→17.7 μm with TH 20), mast-cell infiltration, serum/BALF OVA-specific IgE and Th2 cytokines; TH 20 mg/kg cut lung IL-1β/IL-6/TNF-α without splenic atrophy (unlike dexamethasone).	Th2-cytokine and OVA-IgE suppression; reduced mast-cell/eosinophil infiltration
Kim SK 2008 [49]	Republic of Korea	*In vivo*	Brown alga (*Ecklonia cava*)	*E. cava* 95% ethanolic crude extract (phlorotannin-rich)	OVA-sensitized/challenged asthma in female BALB/c mice	20 mg/kg/day i.p. (figures state 40 mg/kg) on days 16–20 (5 days)	Reduced eosinophil influx; cut IL-4 66%, IL-5 84%, TNF-α 73%; reduced AHR; lowered OVA-	Th2 inhibition without Th1 augmentation; SOCS-3 downregulation;

Study (Author Year)	Country	Design	Marine source (species)	Compound (class)	Model system	Dose / route / duration	Key outcomes & main findings	Main mechanism / pathway
							specific IgE 67%; reduced MMP-9, EPO (~42%), SOCS-3.	reduced MMP-9/EPO/IgE
Lee Dae-Sung 2011 [47]	Republic of Korea	*In vitro* + *in vivo*	Red seaweed (*Polyopes affinis*)	*P. affinis* 95% ethanolic polyphenolic extract (phlorotannin-rich)	OVA asthma in female BALB/c mice; cell-free ESR antioxidant assays	20 mg/kg/day i.p. on days 16–20; *in vitro* up to 200 μg/mL	At 200 μg/mL scavenged DPPH 39%, superoxide 85%, hydroxyl 69%, peroxyl 74%; *in vivo* reduced BAL eosinophils, IL-4/IL-5, AHR, EPO (~35%) and OVA-specific IgE (72%).	Phlorotannin antioxidant/radical scavenging; Th2 suppression
Lee 2016 [52]	Republic of Korea	*In vivo*	Sea cucumber (*Stichopus / Apostichopus japonicus*)	Sea-cucumber aqueous/ethanol crude extract (EPA/DHA constituents)	OVA/alum allergic airway inflammation in female C57BL/6 mice	Extract (10× diluted, 200 μl) oral gavage every other day up to 7× before induction (days 1–13)	Decreased AHR and lung eosinophils; lowered Th2 cytokines (IL-4/5/13), raised IL-10/TGF-β and Foxp3; increased CD4+CD25+Foxp3+ Treg ratio in MLN.	Treg activation (IL-10/TGF-β, Foxp3); Th2 suppression
Lee 2018 [53]	Republic of Korea	*In vitro* + *in vivo*	Sea cucumber (*Apostichopus japonicus*)	n-hexane (fatty-acid-enriched) fraction (PUFAs/EPA/palmitoleic/BCFA)	OVA/alum allergic airway inflammation in female C57BL/6 mice; primary splenocyte Il10 assay	Oil-phase extract ~10 mg/dose oral 7× at 2-day intervals before induction; *in vitro* 1 μg/mL on splenocytes	Only the n-hexane phase raised splenocyte Il10; *in vivo* decreased AHR, eosinophilia and goblet-cell hyperplasia, lowered Th2/Th17 cytokines, raised TGF-β/IL-10 and Foxp3+ cells.	Fatty-acid induction of Foxp3+ Treg and IL-10/TGF-β, countering Th2/Th17
Lee 2025 (*Codium fragile*) [40]	Republic of Korea	*In vitro* + *in vivo*	Green seaweed (*Codium fragile*)	Hot-water extract WCF; marker oleamide (fatty-acid amide)	OVA allergic asthma in female BALB/c mice (n=15/group); HPLC-PDA oleamide validation	WCF 50/100/200 mg/kg oral once daily for 1 week from day 31	WCF200 reduced CD4+/CD8+ and Th2 cells/Th2:Th1 ratio (Th1 up), cut BALF eosinophils (3.31→1.76), IL-33/IL-5/IL-13/IL-1β and OVA-IgE; restored histology, reduced MDA, raised GSH/SOD.	Th2 suppression; NF-κB and TGF-β1/Smad inhibition; reduced oxidative stress and JNK/BAX/caspase-3 apoptosis
Liu 2025 (Alternariol) [29]	China	*In vitro* + *in vivo*	Deep-sea marine fungus (*Alternaria sp.*, strain Xia 20)	Alternariol / AOH (dibenzo-α-pyrone polyketide)	OVA-OT-II CD4+ T-cell differentiation *in vitro*; C57BL/6J OVA/Alum allergic airway mice	8 mg/kg oral gavage; homeostasis 14 days, lung-injury arm 3 days during immunization + daily through challenge (days 18–21)	Dose-dependently suppressed Th1 cytokines (IFN-γ, IL-2, TNF-α), induced ~30% T-cell apoptosis; *in vivo* attenuated OVA-induced lung injury and immune-cell infiltration (APC-independent).	Inhibits early T-cell activation and Th1 TFs (T-bet, Eomes); impairs migration; mild apoptosis
Mao 2005 [59]	USA	Clinical RCT	Cyanobacterium (*Spirulina platensis/Arthrospira*)	*Spirulina supplement*(active C-phycocyanin)	Allergic rhinitis patients (36 enrolled, 18–55 yr); double-blind crossover; PHA-stimulated PBMC	1,000 or 2,000 mg/day oral capsules × 12 weeks	*Spirulina*2,000 mg/day reduced IL-4 by 32% (P=0.0082); 1,000 mg/day not significant; no change in IFN-γ or IL-2.	Th2 suppression via IL-4 inhibition
Maruyama 2005 [46]	Japan	*In vivo*	Brown alga (*Undaria pinnatifida*, mekabu)	Mekabu fucoidan (sulfated polysaccharide)	OVA/alum + nebulized-OVA asthma model in male BALB/c mice (n=20/group)	50 mg/kg i.p. daily × 4 days before sensitization	Significantly decreased BALF IL-4/IL-5/IL-13 and total/OVA-specific IgE; eosinophils absent in treated mice; near-normal lung histology (IFN-γ unchanged).	Downregulation of Th2 responses (cytokines + IgE) without IFN-γ augmentation
Mickleborough 2013 [37]	USA	Clinical RCT	NZ green-lipped mussel (*Perna canaliculus*)	PCSO-524 / Lyprinol (omega-3 PUFA extract, EPA+DHA)	20 mild-moderate asthmatic adults with hyperpnea-induced bronchoconstriction	8 caps/day (~72 mg EPA + 48 mg DHA) oral × 3 weeks; double-blind crossover (2-wk washout)	Reduced max post-EVH FEV1 fall (8.4% vs 19.3% usual / 22.5% placebo, p<0.05); reduced EBC cysteinyl-LTs, 8-isoprostane, urinary PGF2, CC16; improved symptoms/rescue use.	Pro-resolving/anti-inflammatory; EPA competes with arachidonic acid for COX-2/5-LO
Mølsæter 2025 [38]	Norway	Clinical RCT	Atlantic salmon (*Salmo salar*)	OmeGO unrefined salmon oil (n-3 PUFAs EPA, DHA, DPA)	Adults (18–75 yr) with type-2 eosinophilic asthma; 70 enrolled, multicenter	6 g/day oral × 20 weeks; double-blind vs MCT placebo	No significant difference in exacerbation rates; median time to first composite event 37 d (OmeGO) vs 15 d (placebo), p=0.347; weekly event frequency similar.	n-3 PUFA-derived specialized pro-resolving lipid mediators modulating type-2 inflammation
Park WS 2013 [48]	Republic of Korea	*In vivo*	Red marine alga (*Callophyllis japonica*)	*C. japonica* ethanol extract CJE (phenolic-rich)	OVA allergic asthma in female BALB/c mice (n=6/group); cell-free ESR assays	20 mg/kg i.p. on days 16–20	Strong radical scavenging (DPPH 71%, alkyl 79%, superoxide 76%); reduced BAL eosinophils, AHR (Penh), Th2 cytokines (IL-4/IL-5) and OVA-specific IgE.	Antioxidant scavenging; suppression of Th2 cytokines, eosinophils and IgE
Sanjeewa KK 2019 [54]	Republic of Korea	*In vivo*	Brown seaweed (*Sargassum horneri*)	*S. horneri* 70% ethanol extract SHE (polyphenol/flavonoid-rich)	OVA + PM-induced lung inflammation in BALB/c mice (7 groups, n=4); prednisone control	200 or 400 mg/kg oral before daily PM exposure (PM × 7 days)	Reduced MAPK phosphorylation, iNOS, COX2 and TNF-α/IL-1β/IL-6; up-regulated Nrf2/HO-1; 400 mg/kg comparable to prednisone.	MAPK suppression → reduced iNOS/COX2/cytokines; Nrf2/HO-1 upregulation
Strangman 2009 [30]	USA	*In vitro*	Marine-derived actinomycete (*Streptomyces sp.*, CNQ431)	Splenocins A–J (9-membered bis-lactones / antimycin-class polyketides)	Ex vivo OVA-immunized BALB/c mouse splenocyte cytokine bioassay; dexamethasone comparator	Pure compounds in DMSO, 48 h; IC50 2–50 nM	Compounds 1–9 gave low-nanomolar suppression of TH2 cytokines (IL-5, IL-13) and APC cytokines (IL-1, TNF-α), comparable to	Suppression of cytokine production at the APC–TH2 interface (antimycin-related)

Study (Author Year)	Country	Design	Marine source (species)	Compound (class)	Model system	Dose / route / duration	Key outcomes & main findings	Main mechanism / pathway
							dexamethasone; minimal cytotoxicity (therapeutic ratio >200).	
Wood 2010 [51]	Australia	*In vivo*	NZ green-lipped mussel (*Perna canaliculus*, Lyprinol)	Lyprinol (omega-3 PUFA extract; EPA 5.0%, DHA 3.9%); fish-oil comparator	OVA-induced allergic airway disease in male BALB/c mice, low-omega-3 diet	200 µL Lyprinol or fish oil daily gavage from 14 days before sensitization	Lyprinol (not fish oil) reduced eosinophil influx (BALF, lung, blood), mucus hypersecretion and AHR; no change in serum IgG1/IgG2a or IL-4/IL-5/IL-13/IFN-γ.	Suppression of eosinophilic inflammation/AHR independent of Th2 cytokines; lipid-mediator modulation
Wu 2021 [18]	Taiwan	*In vitro* + *in vivo*	Brown algae / marine diatoms	Fucoxanthin (marine carotenoid/xanthophyll)	Human BEAS-2B cells + THP-1 adhesion *in vitro*; female BALB/c OVA asthma *in vivo* (n=10/group); prednisolone control	*In vitro* 0–30 µM 1 h pretreatment; *in vivo* 10 or 30 mg/kg i.p., 1 h before challenges	Decreased THP-1 adhesion, cytokines, eotaxin and ROS in BEAS-2B; *in vivo* alleviated AHR, goblet hyperplasia, eosinophilia, BALF Th2 cytokines; raised GSH/SOD, reduced MDA. Comparable to prednisolone.	Antioxidant (GSH/SOD up, MDA/ROS down) and anti-inflammatory (Th2 cytokines, eotaxin, adhesion)
Yang CH 2015 [45]	Taiwan	*In vitro*	Marine brown algae/seaweed (species NR)	Oligo-fucoidan (low-MW sulfated polysaccharide)	Primary rat (Brown Norway) airway smooth-muscle cells; PDGF-BB-stimulated proliferation	100/500/1000 µg/mL with 40 ng/mL PDGF-BB for 24/48/72 h	Reduced PDGF-stimulated ASMC proliferation 75–99% at 48 h and induced G1/G0 arrest without apoptosis; outperformed budesonide/fenoterol; reduced ERK1/2, Akt, NF-κB phosphorylation.	Suppression of ERK1/2 and Akt and NF-κB activation → reduced proliferation / G1 arrest
Yeom 2021 [24]	Republic of Korea	*In vitro*	Marine sponge (*Luffariella variabilis*)	Manoalide (sesterterpenoid)	Human mast-cell line HMC-1 stimulated with PMACI	0.01/0.1/1 ng/mL, 1 h pretreatment then PMACI (8 h cytokines / 30 min MAPK)	Reduced IL-1β, TSLP, TNF-α, IL-6, IL-8 from PMACI-activated HMC-1 via caspase-1 blockade, without cytotoxicity; attenuated NF-κB and MAPK activation.	Inhibition of caspase-1 and NF-κB; attenuation of MAPK (p38/JNK/ERK)
Yu ZN 2024 [56]	Republic of Korea	*In vivo*	Brown seaweed (*Undaria pinnatifida*)	*U. pinnatifida* extract UPE (70% ethanol, galactofucan-rich)	OVA allergic rhinitis in male BALB/c mice (6 groups, n=6); rat peritoneal mast cells (C48/80); dexamethasone control	50/100/200 mg/kg oral once daily days 16–28, 1 h before challenge	Dose-dependently ameliorated nasal symptoms, mucosal swelling and goblet hyperplasia; rebalanced Th1/Th2 and Th17/Treg; reduced eosinophil/mast-cell migration and anti-OVA IgE/IgG1; inhibited C48/80 RPMC degranulation.	NF-κB and MAPK (p38/ERK/JNK) suppression; Th1/Th2 and Th17/Treg rebalance
Yu ZN 2025 [57]	Republic of Korea	*In vivo*	Brown seaweed (*Undaria pinnatifida*)	*U. pinnatifida* extract UPE (70% ethanol, galactan/galactofucan-rich)	OVA-induced combined allergic rhinitis & asthma syndrome (CARAS) in male BALB/c mice (6 groups, n=6); dexamethasone control	50/100/200 mg/kg oral once daily days 15–30, 1 h before intranasal challenge	Reduced OVA-specific immunoglobulins and symptoms; attenuated NALF/BALF inflammatory cells and Th2 cytokines; protected nasal epithelial barrier (E-cadherin, ZO-1, occludin); enhanced antioxidants; downregulated IL-33.	Tight-junction protection, Nrf-2/HO-1/SOD antioxidant upregulation, IL-33 downregulation, NF-κB/MAPK suppression

Table S1B - Acute lung injury and ARDS (n = 14)

Study (Author Year)	Country	Design	Marine source (species)	Compound (class)	Model system	Dose / route / duration	Key outcomes & main findings	Main mechanism / pathway
Abuohashish 2021 [67]	Saudi Arabia	*In vivo*	Marine probiotic bacterium (*Enterococcus faecium* EA9, shrimp gut)	*E. faecium* EA9 (marine probiotic lactic-acid bacterium)	CLP sepsis-induced ALI in male Wistar rats	1×10^7 CFU/mL, 250 µL oral gavage daily × 10 days pre-CLP	Pretreatment attenuated lung-injury score and edema; reduced lung TNF-α, IL-6, IL-1β and TBARS; restored GSH and CAT/SOD/GPx/GR.	Antioxidant and anti-inflammatory (no specific signaling pathway assayed)
Alharbi R.M. 2020 [68]	Saudi Arabia	*In vivo*	Brown alga (*Hydroclathrus clathratus*, Red Sea)	Ethanolic extract of *H. clathratus* (crude brown-algal extract)	$CuSO_4$-induced lung injury in male albino rats	100 mg/kg oral daily × 28 days (co-administered with $CuSO_4$)	Co-treatment ameliorated MDA, restored GSH/GST, improved histology; downregulated caspase-3/Bax and upregulated Bcl-2 (p<0.001).	Antioxidant and anti-apoptotic (caspase-3/Bax down, Bcl-2 up)
Fagbohun 2024 [66]	Canada	*In vivo*	Sea cucumber (*Cucumaria frondosa*) (+ terrestrial wild blueberry)	Sea-cucumber saponin-rich extract (frondoside A) ± blueberry anthocyanins	Intranasal LPS (P. aeruginosa)-induced ALI in male C57BL/6 mice	Oral dietary, SC ~27.7 mg/mouse/day (frondoside A ~1 mg/kg/day) daily × 7 days pre-LPS	SC repressed NF-κB/MAPK and lowered IL-1β/IL-6/TNF; SC+blueberry combination synergistically reduced ALI lesions and pro-inflammatory cytokines.	Suppression of NF-κB and MAPK (JNK/ERK); synergistic combination
Fang 2023 [43]	China	*In vitro* + *in vivo*	DHA from marine microalgae oil (species not named)	DHA-ethanolamine derivative J9 (omega-3 PUFA ethanolamine ester; Nur77 modulator)	LPS RAW264.7 macrophages, A549; intratracheal LPS-induced ALI in C57BL/6 mice	*In vitro* ≤10 µM; *in vivo* 10/20/30 mg/kg i.p. daily × 1 week pre-LPS	J9 suppressed IL-1β/IL-6/TNF-α (effect lost on Nur77 knockdown); binds Nur77-LBD (KD 8.58 µM); *in vivo* 30 mg/kg reduced BALF/lung cytokines and histopathology.	Binds Nur77 (NR4A1) → inhibits NF-κB Nur77-dependently

Study (Author Year)	Country	Design	Marine source (species)	Compound (class)	Model system	Dose / route / duration	Key outcomes & main findings	Main mechanism / pathway
Gao 2024 [33]	China	*In vitro + in vivo*	Marine sponge (*Pericharax heteroraphis*)	Naamidine J (marine imidazole alkaloid)	LPS RAW264.7 macrophages, BMDMs, HEK293T; LPS-induced ALI mice	*In vitro* 5 μM, 24 h; *in vivo* 20 mg/kg (route/duration in SI)	Inhibited LPS-induced TNF-α/IL-1β/IL-6; binds CSE1L (KD 5.41 μM); *in vivo* reduced lung damage, edema, M1 and increased M2 macrophages; effect abolished by CSE1L knockdown.	Binds CSE1L (exportin-2) → blocks SP1 nuclear translocation → suppresses macrophage inflammation
Lee SH 2025 (*Endarachne binghamiae*) [65]	Republic of Korea	*In vitro + in vivo*	Brown alga (*Endarachne binghamiae*, Jeju)	Hot-water extract of E. binghamiae (EB-WE; crude aqueous extract)	LPS RAW264.7 macrophages; intranasal LPS-induced ALI in male BALB/c mice	*In vitro* 0–500 μg/mL; *in vivo* 2 or 5 mg/mouse oral daily × 3 days pre-LPS	Non-cytotoxic to 500 μg/mL; inhibited NO, TNF-α, IL-6 and COX-2/iNOS mRNA; *in vivo* reduced BALF/serum cytokines, fever and pulmonary edema; high dose approached dexamethasone.	Downregulation of MAPK (ERK/JNK/p38), NF-κB and PI3K/AKT signaling
Leung 2013 [63]	Taiwan	*In vivo*	Blue-green alga (*Spirulina platensis*)	C-Phycocyanin (CPC; biliprotein)	Intratracheal LPS-induced ALI in male Sprague-Dawley rats	50 mg/kg i.p. once, 3 h after LPS (post-treatment)	Post-treatment reduced BALF protein, PMN influx, edema, MPO and BALF TNF-α/IL-1β/IL-6/CINC-3 ($P<0.001$); reduced oxidative/nitrosative stress and improved histology.	NF-κB suppression → lower iNOS/COX-2/cytokines; anti-apoptotic (Bax/caspase-3 down, Bcl-2 up)
Li X 2021 [19]	China	*In vitro + in vivo*	Brown seaweed (*Undaria pinnatifida*, *Saccharina japonica*, *Sargassum fulvellum*)	Fucoxanthin (carotenoid/xanthophyll)	LPS RAW264.7 macrophages; intratracheal LPS-induced ALI mice	*In vitro* 5/10/20 μM, 1 h pre-LPS; *in vivo* 10 mg/kg i.v., 30 min pre-LPS	Dose-dependently suppressed COX-2/iNOS and IL-6/TNF-α/IL-1β; binds TLR4 (docking); *in vivo* reversed BALF protein/cell rises, edema and lung CD68/COX-2/iNOS.	Inhibits TLR4/MyD88/NF-κB axis by directly targeting TLR4
Nie 2018 [111]	China	*In vivo*	Brown seaweed (*Fucus vesiculosus*)	Fucoidan (sulfated polysaccharide)	Hyperoxic (100% O2, 36 h) lung injury in BALB/c mice	100 μg/mL nebulized inhalation, concurrent with 36 h hyperoxia	Reversed weight loss/lung weight gain; reduced BALF cells, macrophage/CD68 markers and IL-1/IL-6/TNF-α; attenuated alveolar wall thickening.	Inhibition of ERK1/2 phosphorylation → lower cytokines and inflammatory infiltration
XingXing Zhang 2024 [62]	China	*In vitro + in vivo*	Algal/seaweed polysaccharides (species NR)	Algal polysaccharides (APs; subtype unidentified)	LPS BEAS-2B cells; LPS (i.p.) sepsis-associated ALI in male BALB/c mice	*In vitro* 50 μg/mL, 24 h; *in vivo* 50 mg/kg aerosolized every 12 h × 3 over 48 h	Reduced LDH/NO/ROS/MDA, restored SOD/GSH, lowered iNOS/COX-2/cytokines; *in vivo* reduced edema, injury score, neutrophils, CXCL2/MPO; efficacy approached dexamethasone.	Inhibits TLR4/NF-κB and activates Nrf2/HO-1 (confirmed by ML385)
Zeng 2025 [61]	China	*In vitro + in vivo*	Brown algae (laminarin; species NR)	Laminarin (low-MW β-glucan polysaccharide)	LPS RAW264.7 macrophages, septic-patient PBMCs; LPS (i.p.) sepsis/ALI in male C57BL/6J mice	*In vivo* 800 mg/kg i.p. once, 1 h post-LPS; *in vitro* 0.3125 mg/mL, 24 h	Improved survival ~49%; reduced serum TNF-α (~37%) and IL-6 (~29%), edema, BALF protein/neutrophils and lung M1 polarization; HIF-1α knockdown abolished effects.	Downregulates HIF-1α → lower glycolysis, ROS and NLRP3 → inhibits M1 polarization
Zhang 2022 [64]	China	*In vitro + in vivo*	Cyanobacterium (*Spirulina platensis*)	C-Phycocyanin (C-PC; biliprotein)	Seawater + LPS (i.t.) ALI in male C57BL/6 mice; RAW264.7 and MLE-12 cells	*In vivo* 100/200/400 mg/kg i.p. daily × 7 days; *in vitro* 120 μg/mL	Dose-dependently reduced edema, MPO, BALF protein and serum IL-1β/TNF-α/IL-6/MCP-1; reduced oxidative stress and NLRP3/ASC/caspase-1; reduced epithelial apoptosis.	Inhibits NF-κB/NLRP3 inflammasome in macrophages; anti-apoptotic in epithelium
Zhang (*Costaria costata*) 2024 [60]	Republic of Korea	*In vitro + in vivo*	Brown alga (*Costaria costata*)	Fucoidan from C. costata (FCC; sulfated polysaccharide, 420 kDa)	Mouse BMDCs; LPS-induced lung inflammation and LPS sepsis/survival in C57BL/6 mice	*In vitro* 10/20/50 μg/mL, 1 h pre-LPS; *in vivo* 25/50/100 mg/kg oral daily × 3 days	Suppressed LPS-induced BMDC co-stimulatory/MHC markers and IL-1β/IL-6/IL-12/TNF-α (FCC alone non-activating); *in vivo* reduced lung leukocyte infiltration; sepsis survival 40–50%, comparable to dexamethasone.	Suppression of dendritic-cell activation (co-stimulatory molecules, MHC, cytokines)
Zhang (Fucoidan) 2020 [69]	China	*In vivo*	Brown seaweed/algae (fucoidan; species NR)	Fucoidan (L-fucose-enriched sulfated polysaccharide)	Hyperoxia (80% O2) lung injury/BPD in newborn Sprague-Dawley rat pups; ex vivo lung fibroblasts	80 mg/kg/day i.p. from P8 × 3 days (hyperoxia P1–P22)	Reduced lung W/D ratio, BALF protein/cells/LDH and collagen deposition; decreased apoptosis (Bcl-2/Bax up); restored SOD/GSH, lowered MDA; reduced α-SMA/collagen I.	Suppresses apoptosis and oxidative stress; inhibits fibroblast-to-myofibroblast differentiation

Table S1C - Pulmonary fibrosis and lung remodeling (n = 8)

Study (Author Year)	Country	Design	Marine source (species)	Compound (class)	Model system	Dose / route / duration	Key outcomes & main findings	Main mechanism / pathway
Cai 2024 [31]	China	*In vitro*	Mangrove sediment-derived fungus (*Talaromyces sp.* SCSIO 41412)	Talaroterphenyl A (oxidized p-terphenyl derivative)	TGF-β1-induced MRC-5 lung fibroblasts; LPS-stimulated RAW264.7; PDE4 assay; docking	5/10/20 μM; TGF-β1 48 h, LPS co-treatment	PDE4 IC50 0.62 μM; downregulated FN1, COL1, α-SMA in MRC-5; reduced IL-6, TNF-α, IL-1β in macrophages; low cytotoxicity.	PDE4 inhibition (raises cAMP); reduced FN1/COL1/α-SMA expression

Study (Author Year)	Country	Design	Marine source (species)	Compound (class)	Model system	Dose / route / duration	Key outcomes & main findings	Main mechanism / pathway
Dhaouaf 2023 [72]	Tunisia	*In vivo*	Red seaweed (*Corallina officinalis*)	C. officinalis aqueous extract COAE (crude polyphenol-rich)	Bleomycin-induced lung fibrosis in male Wistar rats	150 or 300 mg/kg oral gavage once daily × 21 days (from day 3 post-BLM)	Dose-dependently reversed MDA, restored SOD/CAT/-SH, reduced BALF inflammatory cells, attenuated histological severity.	Antioxidant (radical scavenging, restored SOD/CAT/thiols); anti-inflammatory
Kim_Choi 2020 [35]	Republic of Korea	*In vitro*	Brown alga (*Sargassum thunbergii*)	Indole-6-carboxaldehyde (I6CA; indole derivative)	H2O2-induced oxidative stress in V79-4 Chinese hamster lung fibroblasts	≤300 μM, 1 h pretreatment before 1 mM H2O2 for 24 h	Blocked H2O2-induced ROS and G2/M arrest, restored viability, reduced apoptosis; ZnPP abolished effects (HO-1 dependence).	Nrf2/HO-1 activation → antioxidant/anti-apoptotic
Li 2023 (Astaxanthin) [21]	China	*In vitro + in vivo*	Astaxanthin from marine organisms (shrimp, oysters, salmon)	Astaxanthin (AST; carotenoid/xanthophyll)	Radiation-induced lung injury in C57BL/6 mice (16 Gy); irradiated MLE-12 cells (8 Gy)	*In vivo* 25 mg/kg i.p. daily 1 wk pre/post irradiation; *in vitro* 30 μM pretreatment 2–12 h	Reduced weight loss, lung coefficient, fibrosis (Masson), inflammatory cytokines and apoptosis; preserved mitochondrial membrane potential *in vitro*.	Inhibition of apoptosis (Bax/cleaved caspase-3 down, MMP preserved); anti-inflammatory/antioxidant
Liu X 2017 [70]	China	*In vitro*	Brown algae (laminarin; species NR)	Laminarin (β-1,3/1,6-glucan polysaccharide)	H2O2-induced oxidative damage in human lung fibroblast MRC-5; NRF2 siRNA/overexpression	~0.020 mg/mL (methods state higher; inconsistent), pre- or post-H2O2 (600 μM, 24 h)	Pre-treatment relieved SOD/MDA/GSH/CAT changes, promoted NRF2 mRNA/protein and nuclear translocation, suppressed KEAP1, induced NQO1/GCLC/HO1.	NRF2/KEAP1 signaling → antioxidant defense
Liu 2022 (Phycocyanin) [71]	China	*In vitro*	*Spirulina*(cyanobacterium)	Phycocyanin peptide (phycobiliprotein-derived)	LPS-stimulated RAW264.7; TGF-β1-induced A549 (EMT/fibrosis); HFL-1; Keap1 docking	Inflammation 50–200 μg/mL (24 h); fibrosis 10–30 μg/mL (72 h)	Dose-dependently reduced NO, TNF-α, IL-6; restored A549 morphology, reduced Collagen I and α-SMA, increased Nrf2/NQO1/HO-1.	Keap1-Nrf2-HO-1 activation; inhibits NF-κB-driven EMT
Ma SY 2017 [20]	Republic of Korea	*In vitro + in vivo*	Brown seaweed (fucoxanthin; species NR)	Fucoxanthin (Fx; carotenoid/xanthophyll)	TGF-β1-stimulated human pulmonary fibroblasts (Smad2/3 siRNA); bleomycin lung fibrosis in C57BL/6 mice	*In vitro* 5/10/30 μM pretreatment 30 min before TGF-β1; *in vivo* 10 mg/kg i.p. daily from day 7, killed day 14	Dose-dependently inhibited α-SMA, type-1 collagen, fibronectin, IL-6 and collagen-gel contraction; *in vivo* reduced BLM fibrosis and collagen.	Inhibition of TGF-β1 signaling - Smad2/3-dependent and -independent (p38 MAPK, PI3K/Akt)
Seo 2025 (*Caulerpa okamurae*) [73]	Republic of Korea	*In vitro + in vivo*	Green seaweed (*Caulerpa okamurae*)	*C. okamurae* extract COE (50% ethanol, carotenoid-rich; fucoxanthin candidate active)	TGF-β-induced MRC-5 fibroblasts; LPS+nigericin BMDMs (NLRP3); bleomycin lung fibrosis in C57BL/6J mice	*In vitro* 50 μg/mL with TGFβ 24–48 h; *in vivo* 2.5 mg/kg oral gavage every 2 days (days 1–15), euthanized day 28	Reduced fibrotic (α-Sma, Mmp1, Col1a1, Vimentin) and inflammatory markers; attenuated *in vivo* fibrosis; inhibited NLRP3 inflammasome.	TGFβ/SMAD2/3 and MAPK inhibition; NLRP3 inflammasome suppression

Table S1D - Respiratory infections (n = 5)

Study (Author Year)	Country	Design	Marine source (species)	Compound (class)	Model system	Dose / route / duration	Key outcomes & main findings	Main mechanism / pathway
Fu 2025 [75]	China	*In vitro + in vivo*	*Sargassum fusiforme* polysaccharide (marine component of CLS combination)	CLS = cordycepin + lactoferrin + S. fusiforme polysaccharide (combination)	RSV (A2) intranasal infection in BALB/c mice (macrophage depletion); primary alveolar macrophages	*In vivo* oral 100 mg/kg cordycepin + 10 mg/animal lactoferrin + 200 mg/kg polysaccharide; euthanized day 7	Improved lung pathology, reduced BALF albumin/LDH and RSV load; effects abrogated by macrophage depletion; promoted M2 polarization.	M2-like alveolar-macrophage polarization; reduced viral load; macrophage-dependent host defense
Mehrbod 2014 [74]	Malaysia	*In vitro*	HESA-A herbal-marine compound (marine component not taxonomically specified)	HESA-A (mixed herbal-marine preparation; organic/inorganic/aqueous fractions)	Influenza A H1N1 (A/NJ/8/76) in MDCK cells; pre-/co-/post-treatment	0.025 mg/mL (non-cytotoxic); pre- (1/8/24 h), concurrent, or post-infection	Pre- and co-treatment significantly protected viability ($p \leq 0.05$); post-infection did not; HA titer reduced in all combos ($p \leq 0.01$).	Inhibition of viral penetration/adsorption in early infection (prophylactic)
Okimura 2020 [77]	Japan	*In vivo*	Edible brown alga (*Ascophyllum nodosum*)	Ascophyllan HS (sulfated polysaccharide preparation)	Cyclophosphamide-immunosuppressed CBA/J mice with intranasal Streptococcus pneumoniae	167 or 500 mg/kg/day oral, before and after infection, 14 consecutive days	Survival improved to 90% (low) and 100% (high) vs 20% control; near-normal lung histology; reduced bacterial burden; raised serum IL-12.	Host immune activation (macrophage/NK/DC, elevated IL-12); reduced bacterial burden
Park SO 2025 [39]	Republic of Korea	*In vitro + in vivo*	Siberian sturgeon (*Acipenser baerii*, sturgeon oil)	Solubilized sturgeon oil SSO (PUFA-rich DHA/EPA nanoemulsion nasal spray)	SARS-CoV-2 in hamsters; IAV (H1N1) and RSV in C57BL/6 mice; Vero E6 cells	Intranasal 50 μL, 3×/day × 3 days, up to 2–24 h pre-challenge	Dose-dependently inhibited SARS-CoV-2, IAV, RSV replication in nasal/lung tissue; reduced morbidity/mortality and inflammation; protection persisted ≥6 h.	Mucosal barrier / inhibition of viral entry-replication at nasal mucosa

Study (Author Year)	Country	Design	Marine source (species)	Compound (class)	Model system	Dose / route / duration	Key outcomes & main findings	Main mechanism / pathway
Richards 2020 [76]	Australia	*In vivo*	Brown seaweed (*Undaria pinnatifida*, wakame)	*U. pinnatifida* fucoidan UPF (fucose-rich sulfated polysaccharide)	Severe influenza A H1N1 (PR8) intranasal infection in female BALB/c mice (treatment + prophylactic)	Treatment 3.52 mg/day oral post-infection (to day 7); prevention 3.52 or 7.04 mg/day in feed from 3 days pre-infection	Reduced gross lung consolidation (treatment); 7.04 mg/day improved clinical signs and lung pathology; NO significant reduction in lung viral titres.	Anti-inflammatory attenuation of lung pathology (not direct antiviral)

Table S1E - Pollution- and exposure-related lung injury (PM + COPD) (n = 10)

Study (Author Year)	Country	Design	Marine source (species)	Compound (class)	Model system	Dose / route / duration	Key outcomes & main findings	Main mechanism / pathway
Choi 2019 [34]	Republic of Korea	*In vitro* + *in vivo*	Red alga-associated *Streptomyces sp.* MC025 (marine actinomycete)	Collismycin C and Pyrisulfoxin A (2,2'-bipyridine alkaloids)	PM2.5-challenged primary mouse lung microvascular endothelial cells + PM2.5 intratracheal BALB/c mouse model	*In vitro* up to 1000 μM 6 h pre then PM2.5 6 h; *in vivo* 0.2–10 mg/kg p.o. × 10 days + intratracheal PM2.5 1 mg/kg × 10 days	Both compounds dose-dependently inhibited PM2.5-induced endothelial hyperpermeability, reduced BAL leukocytes and IL-6/TNF-α; 6-nitrile analogs inactive.	ROS scavenging → p38 MAPK inhibition + PI3K/Akt1 activation → endothelial barrier protection
Fernando 2019 [26]	Republic of Korea	*In vitro*	Brown alga (*Sargassum binderi*)	Fucosterol (phytosterol / marine sterol)	China fine-dust PM (CPM)-stimulated A549 human lung epithelial cells	12.5–25 μg/mL, 1 h pre-treatment; CPM 100 μg/mL, 24 h	Dose-dependently reversed CPM-induced oxidative stress, inflammation and apoptosis (ROS IC50 21.74 μg/mL); raised SOD/CAT/HO-1/Nrf2, reduced Bax/caspases/COX-2/cytokines.	Nrf2/antioxidant induction; anti-apoptotic and anti-inflammatory (NF-κB/MAPK, COX-2/PGE2)
Herath 2021 [55]	Republic of Korea	*In vitro*	Brown alga (*Sargassum horneri*)	Ethanol extract SHE (polyphenol-rich)	PM (urban aerosol)-stimulated MLE-12 murine type-II alveolar epithelial cells	3.9–250 μg/mL (mechanistic 62.5/125 μg/mL) + PM 125 μg/mL, 3/24/48 h	Attenuated PM-induced TLR2/4/7 and MAPK phosphorylation; reduced proinflammatory/pro-allergic cytokines and chemokines (IL-1β, TNF-α, IL-6, IL-8, MCP-1, CCL5, TSLP, IL-33).	Inhibition of TLR2/4/7-MyD88-TRAF6 → blockade of p38/ERK/JNK MAPK
Jang 2016 (*Undariopsis*) [23]	USA	*In vitro*	Brown alga (*Undariopsis peteseniana*)	apo-9'-fucoxanthinone (Apo9F; apo-carotenoid)	Cigarette smoke extract (CSE)-exposed HBEC2 and BEAS-2B human bronchial epithelial cells	9 compounds 0–50 μM; mechanistic 50 μM Apo9F ± 5% CSE, 24 h	Apo9F gave greatest protection of 9 screened compounds (~30% more viable cells; ~60% less cytotoxicity in BEAS-2B) and reduced CSE-induced apoptosis, DNA damage and mitochondrial ROS.	Reduction of mitochondrial ROS → protection from CSE-induced DNA damage and apoptosis
Keeler 2019 [32]	USA	*In vitro*	Marine dinoflagellate (*Karenia brevis*)	Brevenal (marine polyether ladder compound)	LPS-stimulated A549, MH-S and RAW 264.7 cell lines	0.1 pM–100 nM; then LPS 400 ng/mL, 24 h	Non-toxic up to 100 nM; reduced LPS-induced proinflammatory cytokines while preserving IL-10; did not alter surface TLR4 but reduced M1 (CD86) and M2 (CD206) markers.	Immunomodulation of macrophage M1/M2 phenotype without altering TLR4
Kubo 2019 [22]	Japan	*In vivo*	Marine microalga (Haematococcus pluvialis)	Astaxanthin (xanthophyll carotenoid)	Cigarette-smoke-induced emphysema/COPD model in C57BL/6 mice	Dietary 0.02% (w/w); smoke exposure 60 min/day, 5 days/wk × 12 weeks	Increased Nrf2 mRNA and Nrf2/HO-1 protein, reduced BALF total cells/macrophages/neutrophils, and significantly lowered MLI and destructive index (emphysema suppressed); lymphocytes not attenuated.	Activation of Nrf2-ARE → increased HO-1 → reduced oxidative stress and inflammation
Liyanage 2023 [78]	Republic of Korea	*In vitro*	Brown seaweed (*Sargassum swartzii*)	Sulfated polysaccharides SPSS (fucoidan-type)	PM (urban aerosol)-stimulated MH-S murine alveolar macrophages	Non-toxic range, 1 h pre-treatment; PM 31.3 μg/mL, 24 h	Dose-dependently down-regulated PM-induced NO, PGE2, TNF-α, IL-1β, IL-6, iNOS, COX-2 and suppressed TLR/NF-κB signaling.	Inhibition of TLR2/3/4/7 → NF-κB (and MAPK) inactivation → reduced iNOS/COX-2 and cytokines
Nagahawatta 2022 [42]	Republic of Korea	*In vitro*	Brown seaweed (*Ecklonia maxima*)	Eckmaxol (phlorotannin)	PM-stimulated MH-S murine alveolar macrophages	21.00–84.12 μM + PM, 24 h	Dose-dependently restored viability and down-regulated NO, PGE-2, IL-1β/IL-6/TNF-α, iNOS, COX-2; suppressed TLRs, NF-κB (p50/p65), JNK and p38.	Suppression of TLRs → NF-κB (p50/p65) and MAPK (JNK, p38) → reduced iNOS/COX-2
Park SH 2022 [79]	Republic of Korea	*In vitro* + *in vivo*	*Prasiola japonica* (edible aquatic green alga)	Chloroform fraction of ethanol extract Pj-EE-CF (mixed phytochemical)	UPM 1648a-stimulated BEAS-2B cells + intranasal UPM 1648a ICR mouse lung-injury model	*In vitro* 50/100 μg/mL + UPM 300 μg/mL 24 h; *in vivo* 25/50/100 mg/kg oral BID + intranasal UPM 50 mg/kg once daily × 3 days	Alleviated lung wet/dry ratio, injury score and BALF protein (up to ~99%) and down-regulated cytokines (~95%) and NF-κB activation; reduced ROS *in vitro.*	Suppression of UPM-induced NF-κB (p-IκBα), ROS and iNOS; antioxidant/anti-inflammatory
Sanjeewa 2020 [113]	Republic of Korea	*In vitro*	Brown seaweed (*Sargassum horneri*)	70% ethanol extract SHE (polyphenol-containing)	Urban PM (UPM)-stimulated MH-S murine alveolar macrophages	15.6–250 μg/mL, 1 h pre-treatment; PM 31.3 μg/mL, 24 h	Dose-dependently repressed PM-induced NO, PGE2, TNF-α, IL-1β, IL-6, iNOS, COX-2 and reduced NF-κB/MAPK phosphorylation and TLR2/3/4/7 mRNA.	Blockade of PM-induced TLR-mediated NF-κB and MAPK activation

Note to Table S1. Duplicate records retrieved under different renderings of the first author's name were identified during full-text assessment and resolved so that each study is listed once: "Hoon Lee 2025" / "Lee SH 2025" (*Endarachne binghamiae*; Table S1B, [65]) and "Asanka Sanjeewa 2019" / "Sanjeewa KK 2019" (*Sargassum horneri*; Table S1A, [54]).

Table S2. SYRCLE risk of bias - in vivo animal studies (n = 44)

Domains: D1 sequence generation; D2 baseline characteristics; D3 allocation concealment; D4 random housing; D5 random outcome assessment; D6 blinding (caregivers/investigators); D7 blinding (outcome assessor); D8 incomplete outcome data; D9 selective outcome reporting; D10 other sources of bias. L = low, U = unclear, H = high. Overall result: 0 Low, 35 Unclear, 9 High.

#	Study (Author Year)	Compound	Disease model	D1	D2	D3	D4	D5	D6	D7	D8	D9	D10	Overall	Key justification (1-2 sentences)
1	Abdelmawgood 2023 [27]	Echinochrome	OVA-induced asthma (mouse)	U	U	U	U	U	U	U	L	L	L	Unclear	"Randomly assigned" with no method; baseline weights not matched; all 32 mice analyzed, no CoI, ethics present - incomplete reporting but no high-risk flaw.
2	Abdelmawgood 2024 [28]	Echinochrome	OVA-induced allergic asthma (mouse)	U	U	U	U	U	U	U	L	L	L	Unclear	"Randomly assigned", method NR; histology scoring not blinded but no imbalance/attrition; ethics and CoI clean.
3	Abu Bakar 2015 [50]	*Eucheuma cottonii* extract (SECE)	OVA-induced allergic asthma (rat)	U	U	U	U	U	U	U	L	L	L	Unclear	"Randomly divided", no method; histology/IHC scoring not stated as blinded; 6/group analyzed, ethics and CoI present.
4	Abuohashish 2021 [67]	*Enterococcus faecium* EA9 (marine probiotic)	CLP sepsis-induced acute lung injury (rat)	U	U	U	U	U	U	L	L	L	L	Unclear	"Randomly allocated", no method, but lung-injury histology "scored blindly" (D7 Low); n=6/group fully analyzed; ethics/CoI clean.
5	Alharbi 2020 [68]	*Hydroclathrus clathratus* ethanol extract	CuSO4-induced lung injury (rat)	U	U	U	U	U	U	U	U	U	H	High	Major unresolved internal inconsistency in sample size (24 rats n=6/group vs "Forty-five male Western rats" in Methods 2.7) and ethics with no approval/reference number - D10 serious flaw.
6	Choi 2019 [34]	Collismycin C / Pyrisulfoxin A (*Streptomyces*)	PM2.5-induced lung injury (mouse)	U	U	U	U	U	U	L	U	L	L	Unclear	Randomization NR; histology read by a blinded observer (D7 Low); n=5/group with no attrition reconciliation; ethics/CoI clean.
7	Coulson & O'Donnell 2000 [25]	Contignasterol (IZP-94,005)	Allergen-induced plasma protein exudation (guinea-pig)	U	U	U	U	U	U	L	U	L	H	High	NO animal ethics statement and NO formal CoI despite industry drug donation/initial expenses from Inflazyme (manufacturer) - D10 serious; objective tracer endpoint keeps D7 Low.
8	Dhaouaf 2023 [72]	*Corallina officinalis*aqueous extract (COAE)	Bleomycin-induced lung fibrosis (rat)	U	U	U	U	U	U	U	L	U	U	Unclear	"Randomly divided", no method; histopathology blinding not stated; minor dosing-frequency discrepancy and no explicit CoI statement, but no confirmed high-risk flaw.
9	Fagbohun 2024 [66]	Sea cucumber + wild blueberry extract	LPS-induced acute lung injury (mouse)	U	L	U	U	U	U	U	L	L	U	Unclear	Per-group baseline weights reported (D2 Low); n=10/group analyzed; industry funding (AKSO) + former-employee author disclosed transparently, so D10 only Unclear, not High.
10	Fang 2023 [43]	DHA-ethanolamine derivative J9	LPS-induced acute lung injury (mouse)	U	U	U	U	U	U	U	U	L	L	Unclear	"Randomly divided", no method; treated-group n not fully numbered (mild attrition uncertainty); ethics/CoI clean, validated LPS-ALI model.
11	Fu 2025 [75]	Cordycepin + lactoferrin +*Sargassum polysaccharide*(CLS)	RSV intranasal infection (mouse)	U	U	U	U	U	U	U	U	L	U	Unclear	Very small n=3/group and multi-component intervention (only one part marine) confound attribution; ethics from a commercial company's committee - concerns but no single confirmed high-risk domain.
12	Gao 2024 [33]	Naamidine J (sponge alkaloid)	LPS-induced acute lung injury (mouse)	U	U	U	U	U	U	U	U	L	L	Unclear	Much *in vivo* methodology in Supporting Information; randomization/baseline NR; variable figure n but no clear bias; ethics/CoI clean, validated model.
13	Herath 2020 [112]	*Sargassum horneri* ethanol extract / mojabanchromanol	OVA + PM-induced asthma (mouse)	U	U	U	U	U	U	U	U	L	L	Unclear	"Randomly divided", no method; group sizes n=4-8 (many analyses n=3) with no attrition account; ethics/CoI present, no high-risk flaw.
14	Kim J 2023 [41]	*Agarum cribrosum*extract / Trifuhalol A	OVA-induced allergic asthma (mouse)	U	U	U	U	U	U	U	U	L	U	Unclear	"Divided" (not even "randomly"); N=6 in Methods vs N=5 in figures (possible unexplained attrition, D8 Unclear); one author employed by Haizhibao GmbH (D10 Unclear).
15	Kim SK 2008 [49]	*Ecklonia cava* ethanol extract	OVA-induced asthma (mouse)	U	U	U	U	U	U	L	U	L	U	Unclear	Randomization NR but cell counts/histology by independent blinded investigators (D7 Low); inconsistent figure group sizes (D8 Unclear); CoI not reported (D10 Unclear).
16	Kubo 2019 [22]	Astaxanthin (*Haematococcus pluvialis*)	Cigarette-smoke-induced emphysema/COPD (mouse)	U	U	U	U	U	U	L	L	L	U	Unclear	Only study reporting full attrition (2 excluded + 1 death, n=8/group) and blinded BALF counts (D7 Low); D1 NR ("divided"); astaxanthin supplied by FUJIFILM (D10 Unclear).
17	Lee Dae-Sung 2011 [47]	*Polyopes affinis* polyphenolic extract	OVA-induced asthma (mouse)	U	U	U	U	U	U	L	U	L	U	Unclear	Randomization NR; cell counts/histology by independent blinded investigators (D7 Low); inconsistent group sizes (D8 Unclear); CoI not reported (D10 Unclear).

#	Study (Author Year)	Compound	Disease model	D1	D2	D3	D4	D5	D6	D7	D8	D9	D10	Overall	Key justification (1-2 sentences)
18	Lee 2016 [52]	Sea cucumber (*A. japonicus*) extract	OVA/alum allergic airway inflammation (mouse)	U	U	U	U	U	U	U	U	L	U	Unclear	Randomization NR; group sizes not stated, only ">=3 repeats" (D8 Unclear); co-author at S-J Bio with no CoI statement (D10 Unclear); no confirmed high-risk flaw.
19	Lee 2018 [53]	Sea cucumber n-hexane fatty-acid fraction	OVA/alum allergic airway inflammation (mouse)	U	U	U	U	U	U	U	U	L	U	Unclear	Randomization NR; group sizes not stated (D8 Unclear); SJ Bio co-authors despite "no competing interests" (D10 Unclear), but no single confirmed high-risk domain.
20	Lee SH 2025 (*Endarachne binghamiae*) [65]	*Endarachne binghamiae* hot-water extract (EB-WE)	LPS-induced acute lung injury (mouse)	U	U	U	U	U	U	U	L	L	U	Unclear	"Randomly divided", no method; n=7/group analyzed; author employed by Korea Prime Pharmacy disclosed (D10 Unclear); validated LPS-ALI model. Duplicate of "Hoon Lee 2025" (assessed once).
21	Lee 2025 (*Codium fragile*) [40]	*Codium fragile*hot-water extract (WCF) / oleamide	OVA-induced allergic asthma (mouse)	U	U	U	U	U	U	U	H	L	H	High	Severe unexplained attrition (15/group allocated but only n=3-5 analyzed; D8 High) plus inconsistent IACUC approval number/date (GNU-240405-M0076 vs GNU-180927-M0050; D10 High).
22	Leung 2013 [63]	C-Phycocyanin (*Spirulina*)	LPS-induced acute lung injury (rat)	U	U	U	U	U	U	L	L	L	U	Unclear	"Divided randomly", no method; histology by an investigator "initially blinded" (D7 Low); n=5/group; ethics has no approval number (D10 Unclear).
23	Li X 2021 [19]	Fucoxanthin	LPS-induced acute lung injury (mouse)	U	U	U	U	U	U	U	U	L	H	High	NO animal ethics/IACUC approval statement anywhere in the paper, and strain/sex/age/weight not reported at all - D10 serious flaw (missing ethics + undescribed animals).
24	Li 2023 (Astaxanthin) [21]	Astaxanthin	Radiation-induced lung injury (mouse)	U	L	U	U	U	U	U	H	L	L	High	Unexplained attrition: 60 mice into 20/group allocated but key endpoints report only n=5 with no explanation (D8 High); baseline weights reported (D2 Low); ethics/CoI clean.
25	Liu 2025 (Alternariol) [29]	Alternariol (marine fungus)	OVA-induced allergic airway / lung injury (mouse)	U	U	U	U	U	U	U	U	L	U	Unclear	"Randomly divided", no method; 5/group, no attrition account; related differentiation method patented (D10 Unclear); ethics/CoI present, no confirmed high-risk flaw.
26	Ma SY 2017 [20]	Fucoxanthin	Bleomycin-induced pulmonary fibrosis (mouse)	U	U	U	U	U	U	U	U	L	L	Unclear	Randomization NOT described and baseline weights not reported; thin *in vivo* arm ("n=5 in each experiment") but no confirmed imbalance/attrition; ethics/CoI clean, validated model.
27	Maruyama 2005 [46]	Mekabu fucoidan (*Undaria pinnatifida*)	OVA/alum-induced bronchial asthma (mouse)	U	U	U	U	U	U	U	U	L	U	Unclear	Randomization NR; n=20/group stated without analyzed reconciliation; no funding or CoI statement (2005 paper; D10 Unclear); validated OVA model, ethics present.
28	Nie 2018 [111]	Fucoidan (*Fucus vesiculosus*)	Hyperoxic lung injury (mouse)	U	U	U	U	U	U	L	U	L	U	Unclear	NO randomization statement; histology "assessed in a blinded manner" (D7 Low); no randomization/funding/CoI statements (D10 Unclear) and approving institution differs from authors; no confirmed high-risk domain.
29	Okimura 2020 [77]	Ascophyllan HS (*Ascophyllum nodosum*)	*S. pneumoniae* infection (immunosuppressed mouse)	U	L	U	U	L	U	L	L	L	H	High	First author affiliated with Hayashikane Sangyo Co. (the manufacturer of the tested ascophyllan HS) despite a "no conflicts" statement - D10 serious commercial CoI; objective survival/CFU endpoints (D7 Low), attrition reported.
30	Park SH 2022 [79]	*Prasiola japonica* chloroform fraction (Pj-EE-CF)	UPM-induced lung injury (mouse)	U	U	U	U	U	U	U	U	L	U	Unclear	"Divided", no randomization; subjective lung-injury score with blinding NOT stated (D7 Unclear); funded by Samcheok *Prasiola japonica* Research Center promoting the test alga (D10 Unclear); ethics present.
31	Park SO 2025 [39]	Solubilized sturgeon oil (SSO) nanoemulsion	SARS-CoV-2 / IAV / RSV infection (hamster/mouse)	U	U	U	U	U	U	L	U	L	H	High	Idiosyncratic patented multi-component nanoemulsion AND two authors employed by Sturgeon Bio Ltd. (manufacturer) - D10 serious commercial CoI + non-validated proprietary formulation; objective virologic endpoints keep D7 Low.
32	Park WS 2013 [48]	*Callophyllis japonica* ethanol extract (CJE)	OVA-induced allergic asthma (mouse)	U	U	U	U	U	U	U	L	L	U	Unclear	Randomization NR; n=6/group across 5 experiments; AHR by Penh; no explicit CoI statement (D10 Unclear); ethics present, no confirmed high-risk flaw.
33	Richards 2020 [76]	*Undaria pinnatifida* fucoidan (UPF, Marinova)	Influenza A (H1N1) infection (mouse)	U	U	U	U	U	U	U	L	U	H	High	ALL authors are employees of the manufacturer Marinova (or its contract lab) and the study is Marinova-funded - D10 serious commercial CoI; positive findings rest on subjective clinical/consolidation scoring while the objective viral titre was non-significant (D9 Unclear).
34	Sanjeewa KK 2019 [54]	*Sargassum horneri* 70% ethanol extract (SHE)	OVA + PM-induced lung inflammation (mouse)	U	U	U	U	U	U	U	U	L	L	Unclear	"Randomly divided", no method; small n=4/group (legends imply n>=3, possible drop) but not clearly biased; prednisone positive control, ethics/CoI present.
35	Seo 2025 (*Caulerpa okamurae*) [73]	*Caulerpa okamurae* extract (COE) / fucoxanthin	Bleomycin-induced pulmonary fibrosis (mouse)	U	U	U	U	U	U	U	U	L	U	Unclear	Self-described "pilot" with small unequal groups (n=6/5/4) and wide age range (16-28 wk); randomization stated without method; no confirmed imbalance/high-risk domain; ethics/CoI clean.

#	Study (Author Year)	Compound	Disease model	D1	D2	D3	D4	D5	D6	D7	D8	D9	D10	Overall	Key justification (1-2 sentences)
36	Wood 2010 [51]	Lyprinol (Perna canaliculus lipid extract)	OVA-induced allergic airway disease (mouse)	U	U	U	U	U	U	L	U	L	U	Unclear	Randomization NR; histology "examined in a blinded fashion" (D7 Low); industry funder (Seafood Services Australia) + Blackmores product with no CoI statement (D10 Unclear); no single confirmed high-risk domain.
37	Wu 2021 [18]	Fucoxanthin	OVA-induced asthma (mouse)	U	U	U	U	U	U	U	L	L	L	Unclear	"Randomly divided", no method; n=10/group analyzed, prednisolone positive control; AHR by Penh; ethics/CoI clean - incomplete reporting only.
38	XingXing Zhang 2024 [62]	Algal polysaccharides (APs)	Sepsis-associated acute lung injury (mouse)	U	U	U	U	U	U	U	U	L	H	High	Major internal inconsistency: text says "four groups (n=5/group)" but lists FIVE groups and obtained 24 mice (25 implied), and compound is uncharacterized commercial reagent with ethics lacking an approval number - D10 serious.
39	Yu ZN 2024 [56]	*Undaria pinnatifida* extract (UPE)	OVA-induced allergic rhinitis (mouse)	U	U	U	U	U	U	L	L	L	L	Unclear	"Randomly divided", no method; symptom scores by blinded observers (D7 Low); n=6/group, dexamethasone control, ethics/CoI clean - Unclear only on reporting.
40	Yu ZN 2025 [57]	*Undaria pinnatifida* extract (UPE)	OVA-induced CARAS (rhinitis + asthma) (mouse)	U	U	U	U	U	U	L	L	L	L	Unclear	"Randomly assigned", no method; symptom scoring by observers unaware of purpose (D7 Low); n=6/group, dexamethasone control, ethics/CoI clean.
41	Zeng 2025 [61]	Laminarin (brown-algal beta-glucan)	LPS-induced sepsis / acute lung injury (mouse)	U	U	U	U	U	U	U	U	L	L	Unclear	"Divided randomly", no method; histology over "30 random fields" but not blinded; survival n=12 / endpoints n=5 with no reconciliation; dual ethics approvals, CoI clean.
42	Zhang 2022 [64]	C-Phycocyanin (*Spirulina*)	Seawater + LPS aspiration acute lung injury (mouse)	U	U	U	U	U	U	U	U	L	U	Unclear	"Randomly assigned"; n=36 across 6 groups (~6/group); ethics lacks an approval number (D10 Unclear); no confirmed imbalance/attrition/high-risk flaw.
43	Zhang_*Costaria costata* 2024 [60]	Fucoidan from *Costaria costata* (FCC)	LPS lung inflammation + sepsis survival (mouse)	U	U	U	U	U	U	U	U	L	L	Unclear	NO explicit randomization wording; sex/age/weight not reported; n=10 (sepsis)/n=6 (lung) without reconciliation; well-characterized compound, ethics/CoI present - Unclear only.
44	Zhang_Fucoidan 2020 [69]	Fucoidan (brown algae)	Hyperoxia-induced lung injury / BPD (neonatal rat)	U	U	U	U	U	U	U	U	L	U	Unclear	"Randomly divided", no method; ARRIVE adherence claimed but no pup mortality reported in a hyperoxia model (D8 Unclear) and approving institution differs from author affiliation (D10 Unclear); no single confirmed high-risk domain.

Table S3. OHAT risk of bias - *in vitro* studies (n = 15)

Domains: D1 selection bias; D2 confounding/baseline; D3 exposure characterization; D4 outcome characterization; D5 blinding of outcome assessment; D6 attrition; D7 selective reporting; D8 conflicts of interest; D9 other bias. L = low, U = unclear, H = high. Overall result: 0 Low, 13 Unclear, 2 High.

#	Study (Author Year)	Compound	Cell model/system	D1	D2	D3	D4	D5	D6	D7	D8	D9	Overall	Key justification
1	Cai et al. 2024 [31]	Talaroterphenyl A (oxidized p-terphenyl)	MRC-5 human lung fibroblast + RAW264.7 (Procell, catalog given)	U	L	U	L	U	L	U	L	L	Unclear	Cell sources with catalog but passage NR (D1 U); compound characterized by NMR/HRESIMS/X-ray but no numeric purity % (D3 U); good controls (PFD, rolipram), triplicate, academic/government funding, no competing interest.
2	Fernando et al. 2019 [26]	Fucosterol (marine sterol)	A549 human lung epithelial (KCLB)	U	L	U	L	U	L	U	U	U	Unclear	Passage NR (D1 U); fucosterol structurally characterized (GC-MS/MS, FTIR, NMR) but purity % not stated (D3 U); no CoI statement located (D8 U); no positive reference-drug control (D9 U). Government (Marine Biotech, Korea) funded.
3	Herath et al. 2021 [55]	*S. horneri* ethanol extract (polyphenols)	MLE-12 murine type II alveolar epithelial (ATCC)	U	L	U	L	U	L	U	L	U	Unclear	Passage NR (D1 U); polyphenol extract quantified by Folin/HPLC-DAD but is a multi-component extract (D3 U); no reference-drug positive control (D9 U); government funding, CoI declared none.
4	Jang et al. 2016 (*Undariopsis*) [23]	apo-9'-fucoxanthinone (Apo9F)	HBEC2 + BEAS-2B human bronchial epithelial	U	L	U	L	U	L	U	L	L	Unclear	Source/passage not detailed (D1 U); Apo9F identity by LC-MS/NMR (referenced) but purity % not stated (D3 U); DMSO vehicle + 9-compound comparison; non-profit/government funding (VA, NIH, ALA), CoI declared none.
5	Keeler et al. 2019 [32]	Brevenal (marine polyether ladder)	A549 + MH-S + RAW264.7 (ATCC, catalog)	U	L	L	L	U	L	U	L	L	Unclear	Strong methods: brevenal purity >95% by HPLC explicitly certified + characterized (D3 L), LPS uniform, ethanol vehicle + PbTx-2 positive control, n=3, academic funding, CoI none. Only D1 (passage NR), D5 and D7 prevent Low.
6	Kim & Choi 2020 [35]	Indole-6-carboxaldehyde (I6CA)	V79-4 Chinese hamster lung fibroblast (ATCC)	U	L	U	L	U	L	U	L	L	Unclear	Passage NR (D1 U); I6CA from S. thunbergii but supplier/purity/characterization not reported in-text (referenced only) (D3 U); strong controls (NAC, ZnPP, H2O2 model), >=3 experiments, NRF Korea funding, CoI none.
7	Liu X et al. 2017 [70]	Laminarin (beta-glucan)	MRC-5 human lung fibroblast (ATCC)	U	L	U	L	U	L	U	L	H	Unclear	Commercial laminarin (Sigma) with no purity/characterization (D3 U); internal dose inconsistency (0.020 vs 0.1953125 mg/mL) = D9 High, but D9 High does not trigger an overall High under the rule. Government funding, CoI none.
8	Liu et al. 2022 (Phycocyanin) [71]	Phycocyanin peptide (phycocyanobilin-bound)	RAW264.7 + A549 + HFL-1 (commercial)	U	L	U	L	U	L	U	H	L	High	D8 High: a co-author is affiliated with Yantai Jiahui Biotech Co. Ltd., a company that prepared the tested peptide samples - commercial CoI with a stake in the compound, despite a "no conflict" declaration. Otherwise uniform LPS/TGF-b1, n=3, government R&D funding.
9	Liyanage et al. 2023 [78]	Sulfated polysaccharides from *S. swartzii* (SPSS)	MH-S murine alveolar macrophage (ATCC)	U	L	U	L	U	L	U	L	U	Unclear	Passage NR (D1 U); polysaccharide extract with proximate composition (AOAC/BCA/Folin) but not a defined single compound (D3 U); no positive reference control (D9 U); NRF Korea funding, CoI declared none.
10	Mehrbod et al. 2014 [74]	HESA-A (herbal-marine biological preparation)	MDCK (CCL-34, ATCC) + H1N1 influenza	U	L	H	L	U	U	U	L	U	High	D3 High: HESA-A is an uncharacterized mixed herbal-marine preparation (organic/inorganic/aqueous fractions) with no stated purity, no analytical characterization, and only a single concentration (no real dose-response). No antiviral positive control; replicate n not stated (D6 U). Government (Malaysia) funding.
11	Nagahawatta et al. 2022 [42]	Eckmaxol (phlorotannin)	MH-S murine alveolar macrophage (ATCC)	U	L	L	L	U	L	U	L	U	Unclear	Eckmaxol HPLC purity >90% + ESI-MS/HPLC characterized + dose range (D3 L); only passage NR (D1 U), no reference-drug positive control (D9 U), and D5/D7 Unclear prevent Low. University funding, CoI none.
12	Sanjeewa et al. 2020 [113]	*S. horneri* 70% ethanol extract (SHE)	MH-S murine alveolar macrophage (ATCC)	U	L	U	L	U	L	U	L	U	Unclear	Passage NR (D1 U); commercial-grade seaweed ethanol extract (Seojin Biotech, supplier) not numerically characterized in-text (D3 U); no positive reference control (D9 U); supplier is not an author/funder with stake, so D8 stays Low. Government funding, CoI none.
13	Strangman et al. 2009 [30]	Splenocins A–J (9-membered bis-lactones)	Primary BALB/c mouse splenocytes (ex vivo bioassay)	L	L	U	L	U	U	U	U	L	Unclear	Primary cells with source described (D1 L); full structure by 1D/2D NMR + HRMS but no numeric purity % (D3 U); replicate n per condition not specified (D6 U); no CoI declaration in 2009 manuscript (D8 U). Strong positive control (dexamethasone) + IC50 dose-response. NIH-funded.
14	Yang CH et al. 2015 [45]	Oligo-fucoidan (sulfated polysaccharide)	Primary Brown Norway rat ASMCs, passages 6–8	L	L	U	L	U	U	U	L	L	Unclear	Cell system + source + passage 6–8 stated (D1 L); oligo-fucoidan is a vendor gift with no purity %/characterization (D3 U); replicate exact n not always specified (D6 U); strong controls (budesonide, fenoterol, H2O2). Academic/hospital funding, CoI none.
15	Yeom et al. 2021 [24]	Manoalide (sesterterpenoid)	HMC-1 human mast cell line	U	L	U	L	U	L	U	L	L	Unclear	HMC-1 source/passage not stated (D1 U); manoalide vendor and purity % not given (D3 U); uniform PMACI + vehicle + n=3 + ANOVA/Tukey; NRF Korea funding, CoI declared none. D5/D7 Unclear per single-lab norm.

Table S4. Cochrane RoB 2 - randomised controlled trials (n = 5)

Domains: D1 randomization process; D2 deviations from intended interventions; D3 missing outcome data; D4 measurement of the outcome; D5 selection of the reported result. Overall (RoB 2 algorithm): 1 Low (Mickleborough 2013 [37]), 4 Some concerns.

#	Study (Year)	Compound	Population / design	D1 Randomization	D2 Deviations	D3 Missing data	D4 Outcome measurement	D5 Selective reporting	Overall	Justification
1	Cingi 2008 [58]	*Spirulina* (*Spirulina platensis*)	150 adults with allergic rhinitis; double-blind, placebo-controlled, parallel RCT (6 months)	Some concerns	Low	Some concerns	Some concerns	Some concerns	Some concerns	D1: strong allocation concealment (identical pre-coded boxes; "only the production team knew... not released until the end of the study") but sequence-generation method NOT reported ("we set up the groups randomly") → Some concerns. D2: genuine double-blind (patients + physicians), identical matched placebo → Low. D3: 14% attrition (150→129), uneven completers (85 spirulina vs 44 placebo) and no ITT / no missing-data handling (completer analysis) → Some concerns. D4: outcomes are subjective self-reported symptom diaries/VAS plus physician-rated findings, but blinding maintained → Some concerns. D5: no registration / no pre-specified protocol or SAP (pre-registration practice), outcomes appear complete → Some concerns. No domain reaches High, but multiple Some concerns → Some concerns overall.
2	Emelyanov 2002 [36]	Lipid extract of Perna canaliculus (Lyprinol; EPA/DHA)	46 adults, steroid-naive atopic asthma; single-centre, double-blind, parallel-group, placebo-controlled RCT (8 wk)	Low	Low	Low	Some concerns	Some concerns	Some concerns	D1: computer-generated block randomization ("balanced blocks of two"); matched/identical placebo; baseline differences "not significant" → Low. D2: double-blind, placebo-controlled, identical capsules → Low. D3: no post-randomization dropouts ("all randomised patients completed the treatment and were included"); 14 withdrew before randomization; compliance 93–95% → Low. D4: mixed objective (FEV1, PEF, exhaled H_2O_2) + patient-reported diary; assessor blinding not separately stated → Some concerns. D5: no trial registration (pre-registration era), no pre-specified SAP available; reported outcomes appear complete → Some concerns. No High domain; at least one Some concerns → Some concerns overall.
3	Mao 2005 [59]	*Spirulina*(*Spirulina platensis*/Arthrospira; C-phycocyanin)	36 adults with allergic rhinitis; randomized, double-blind, crossover vs placebo (12 wk)	Some concerns	Some concerns	Some concerns	Low	Some concerns	Some concerns	D1: stated "randomized double-blinded crossover"; sequence generation and allocation concealment NOT reported → Some concerns. D2: "double-blinded" with colour-matched inert placebo, but who was blinded is not described → Some concerns. D3: 2 dropouts in Group U (n→10); no sample-size justification, no missing-data handling, no ITT statement → Some concerns. D4: objective ELISA cytokine measurement (IL-4/IFN-γ/IL-2; defined detection limits) → Low. D5: no registration, no protocol/SAP → Some concerns. No High domain but several Some concerns → Some concerns overall.
4	Mickleborough 2013 [37]	PCSO-524 (*Perna canaliculus*; Lyprinol/Omega XL, EPA/DHA)	20 asthmatics with hyperpnea-induced bronchoconstriction; placebo-controlled, double-blind, randomized crossover (8 wk total)	Low	Low	Low	Low	Low	Low	D1: computerized random-number generator (randomizer.org), fixed random block size of two → adequate sequence generation → Low. D2: double-blind; active and placebo capsules identical in appearance and taste → Low. D3: ITT ("primary analysis was on an intention-to-treat basis... all subjects who were randomly assigned"); CONSORT flow reported → Low. D4: objective outcomes (FEV1 per ATS, FeNO, EBC biomarkers, urinary metabolites) → Low. D5: prospectively registered (ClinicalTrials.gov NCT01504646); allocation concealed (PI

#	Study (Year)	Compound	Population / design	D1 Randomization	D2 Deviations	D3 Missing data	D4 Outcome measurement	D5 Selective reporting	Overall	Justification
										informed of material numbers only after data collection/initial analysis); outcomes reported → Low. All domains Low → Low overall. (Industry funder = product manufacturer is noted but, with registration, concealment and objective endpoints, does not raise an RoB 2 domain.)
5	Mølsæter 2025 [38]	OmeGO unrefined salmon oil (*Salmo salar*; EPA/DHA/DPA)	70 adults with type-2 eosinophilic asthma; prospective, randomized, double-blind, placebo-controlled, multicentre (20 wk)	Low	Low	Some concerns	Some concerns	Some concerns	Some concerns	D1: block randomization (variable blocks 2/4), statistician-generated sequence ensuring allocation blinding; identical placebo → Low. D2: double-blind (participants, coordinators, researchers blinded until data-collection complete); identical MCT placebo → Low. D3: planned n=80 (40/group) not reached (66 completed; one withdrew the day after randomization; some tables N=34; primary analyses 33/33); incomplete diary data and missing composite-score components handled only via "non-missing data" / Poisson assumption → Some concerns. D4: objective spirometry/FeNO/PEF but the primary composite endpoint is partly self-reported (diary-based) → Some concerns. D5: prospectively registered (NCT05137132) with a signed SAP prior to unblinding - favourable - BUT the funder Hofseth Biocare ASA manufactures OmeGO, employs the lead doctoral candidate, includes an author (C. Currie), and had a role in study design; this material conflict, combined with the underpowered/incomplete primary endpoint, is weighed as a residual reporting/selection concern → Some concerns. No High domain; multiple Some concerns → Some concerns overall.

Table S5. ROBINS-I - non-randomised clinical study (Denisenko 2023 [44])

ROBINS-I applied to the uncontrolled, open-label before–after study (corrected from RoB 2). Domains D1–D7; overall judgement = most severe domain. Overall: Serious risk of bias (driven by uncontrolled confounding and open-label deviations).

Domain	Rating	Justification
D1 - Confounding	Serious	Uncontrolled before–after design with no concurrent randomized comparator and no statistical adjustment for confounders. All patients continued background ICS/LABA (budesonide + formoterol), so observed changes cannot be separated from continued standard therapy, regression to the mean, or natural variation. The healthy cohort is not a valid control (younger 45.9±8.5 vs 58.3±7.0 yr; lower BMI; not AG-treated). Authors themselves acknowledge "the absence of a robust placebo control or cross-over design." This is the dominant, structurally unavoidable bias.
D2 - Selection of participants	Moderate	Single defined cohort of GINA-controlled/partially-controlled mild asthma + obesity patients (ACQ-5 0.75–1.5); inclusion at the start of follow-up so no obvious selection on outcome. However, small convenience sample (n=19) with no described sampling frame and a non-comparable reference group introduces moderate selection-related concern.
D3 - Classification of interventions	Low	Intervention is clearly and prospectively defined (0.4 g/day pure AGs, 1 capsule of 0.2 g powder twice daily after meals, 3 months); the compound was characterized by GC-MS (chimyl alcohol 91.07±0.21%, batyl alcohol 7.38±0.57%). Intervention status is unambiguous and not influenced by outcome knowledge.
D4 - Deviations from intended interventions	Serious	Open-label, no blinding of participants or care providers. For the patient-influenced/effort-dependent components (ACQ-5 self-report, salbutamol-reversibility effort) and concomitant-care decisions, the absence of blinding creates genuine performance bias that could co-occur with the intervention; no co-intervention balancing is possible in an uncontrolled design. (Rated at the more severe end of the Moderate–Serious range given the fully open-label structure.)
D5 - Missing data	Low	All 19 asthma patients completed the treatment regimen ("All participants successfully completed the treatment regimen"); 35/35 total completed; no dropouts or exclusions reported. Outcome data essentially complete.
D6 - Measurement of outcomes	Moderate	Most endpoints are objective and assessor-independent (spirometry, GC-MS fatty-acid/plasmalogen markers, ELISA for TXB2/LTB4/adipokines, flow-cytometry cytokines). However, a key clinical endpoint, ACQ-5, is patient-reported and collected unblinded, so the self-reported component is susceptible to detection/expectation bias → Moderate.
D7 - Selection of the reported result	Moderate	No trial registration / no pre-specified protocol or SAP identified, so pre-specification of outcomes cannot be verified despite the reported outcomes appearing internally complete. Industry co-funding from BIONIKA LLC, which manufactures the tested supplement (grant BIO_004/2022, funded compound preparation/standardization), is a further reporting/selection concern, even though authors declare no conflict and state funders had no role in design. → Moderate.
Overall	Serious	Per the ROBINS-I algorithm, the overall judgement equals the most severe domain rating. With D1 Confounding = Serious and D4 Deviations = Serious, the study is at Serious risk of bias overall. The result is suggestive but cannot support causal inference from this uncontrolled, open-label, industry-co-funded before–after design.

Summary of risk-of-bias results

Tool (design)	Studies	Low	Unclear / Some concerns	High / Serious
SYRCLE (*in vivo*)	44	0	35	9
OHAT (*in vitro*)	15	0	13	2
Cochrane RoB 2 (RCTs)	5	1	4	0
ROBINS-I (non-randomised)	1	0	0	1 (Serious)
Total	65	1	52	12

Studies at High / Serious risk of bias (12): SYRCLE - Alharbi 2020 [68], Coulson & O'Donnell 2000 [25], Lee 2025 (*Codium fragile*) [40], Li X 2021 [19], Li 2023 (Astaxanthin) [21], Okimura 2020 [77], Park SO 2025 [39], Richards 2020 [76], XingXing Zhang 2024 [62]; OHAT - Liu 2022 (Phycocyanin) [71], Mehrbod 2014 [74]; ROBINS-I - Denisenko 2023 [44]. The high-risk ratings are driven predominantly by commercial conflicts of interest (manufacturer-affiliated authors/funders), unexplained allocated-vs-analysed attrition, internal inconsistencies, missing ethics approval, and - for the in-vitro studies - uncharacterised test material.

Quantitative synthesis (meta-analysis)

Where at least three studies shared a disease model and the same outcome, results were pooled by random-effects meta-analysis (DerSimonian-Laird) using the standardised mean difference (Hedges' g). A positive g indicates that the marine compound reduced the marker relative to the disease-model control (benefit). Heterogeneity was quantified by I-squared and tau-squared; small-study effects by Egger's test (k>=4). Sixty study-level comparisons from 17 studies were pooled into 11 outcomes across two clusters.

Table S6. Pooled random-effects estimates

Cluster	Outcome	Studies (k)	Hedges g	95% CI	p	I^2	τ^2	Egger p
Acute lung injury (LPS)	IL-1beta	3	3.62	[0.41, 6.84]	0.027	88%	6.68	
Acute lung injury (LPS)	IL-6	4	3.06	[0.98, 5.15]	0.004	82%	3.27	0.004
Acute lung injury (LPS)	Lung wet/dry ratio	5	2.85	[1.87, 3.83]	1.1e-08	44%	0.54	0.020
Acute lung injury (LPS)	MPO	3	2.13	[1.31, 2.96]	4.2e-07	0%	0.00	
Acute lung injury (LPS)	TNF-alpha	4	4.45	[2.25, 6.65]	7.5e-05	73%	3.35	0.010
Asthma (OVA)	BALF eosinophils	9	2.23	[1.29, 3.16]	3.1e-06	79%	1.50	0.035
Asthma (OVA)	IL-13	5	1.63	[0.82, 2.43]	7.4e-05	64%	0.51	0.006
Asthma (OVA)	IL-4	7	1.90	[0.89, 2.90]	2.3e-04	79%	1.32	0.013
Asthma (OVA)	IL-5	7	2.18	[0.99, 3.37]	3.4e-04	84%	1.94	0.008
Asthma (OVA)	OVA-specific IgE	10	1.46	[0.82, 2.11]	9.1e-06	68%	0.69	0.017
Asthma (OVA)	Total IgE	3	1.22	[0.21, 2.23]	0.018	73%	0.58	

Supplementary Figures S1–S11. Forest plots

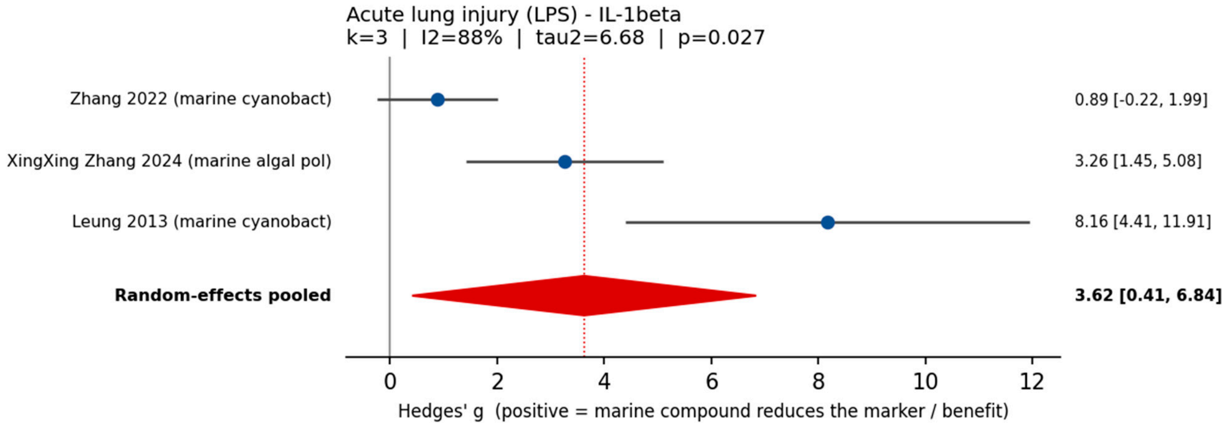

Figure S1. Acute lung injury (LPS) - IL-1beta (k=3).

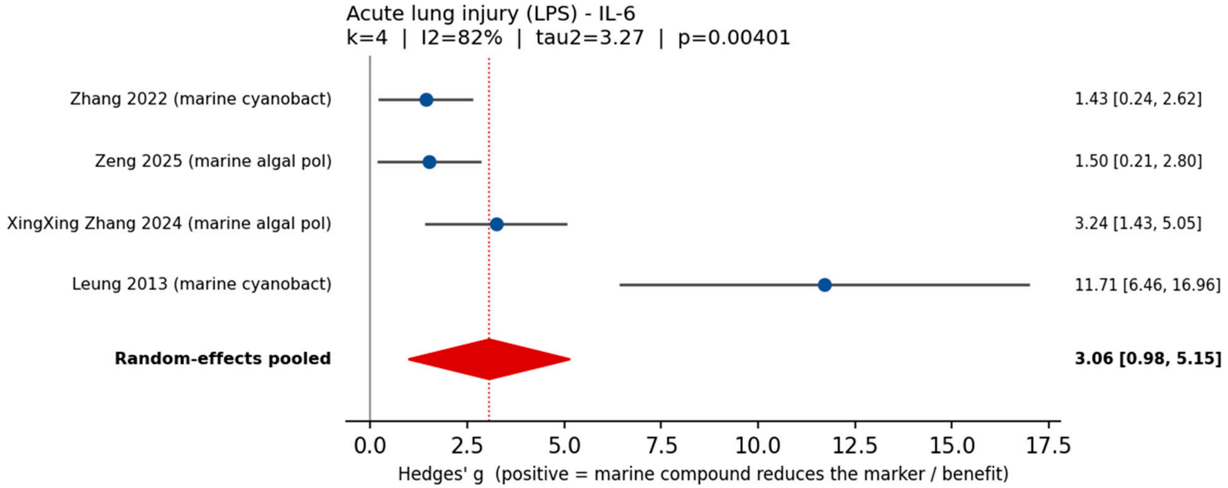

Figure S2. Acute lung injury (LPS) - IL-6 (k=4).

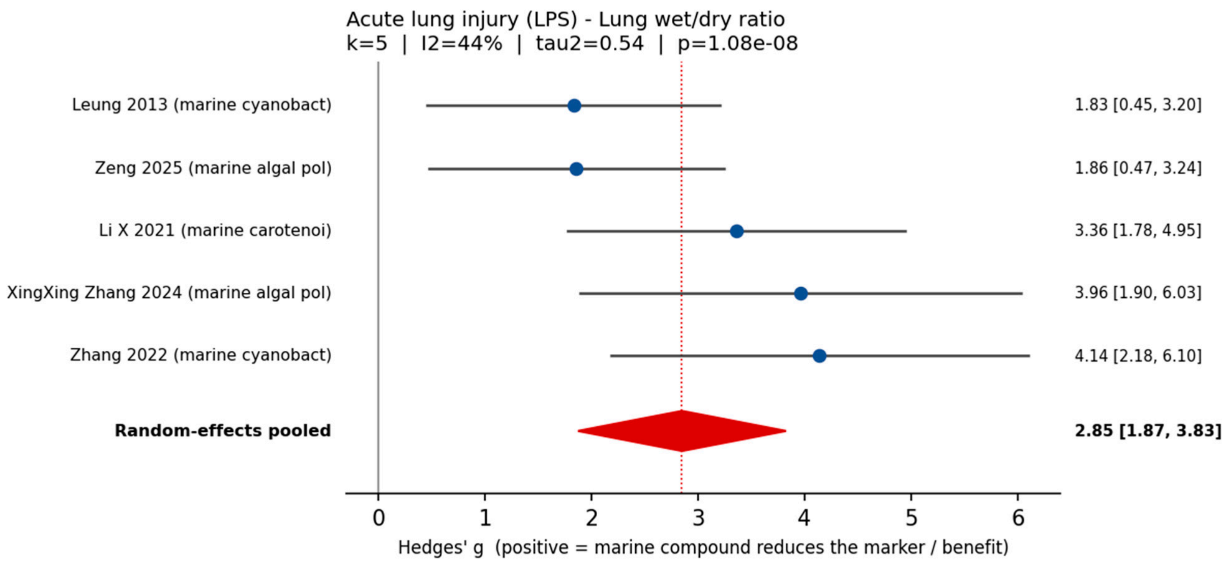

Figure S3. Acute lung injury (LPS) - Lung wet/dry ratio (k=5).

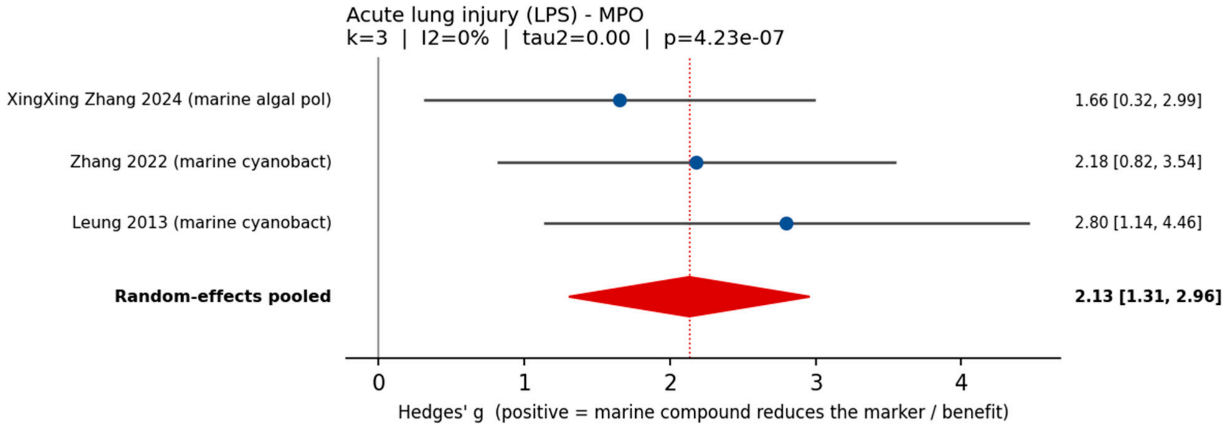

Figure S4. Acute lung injury (LPS) - MPO (k=3).

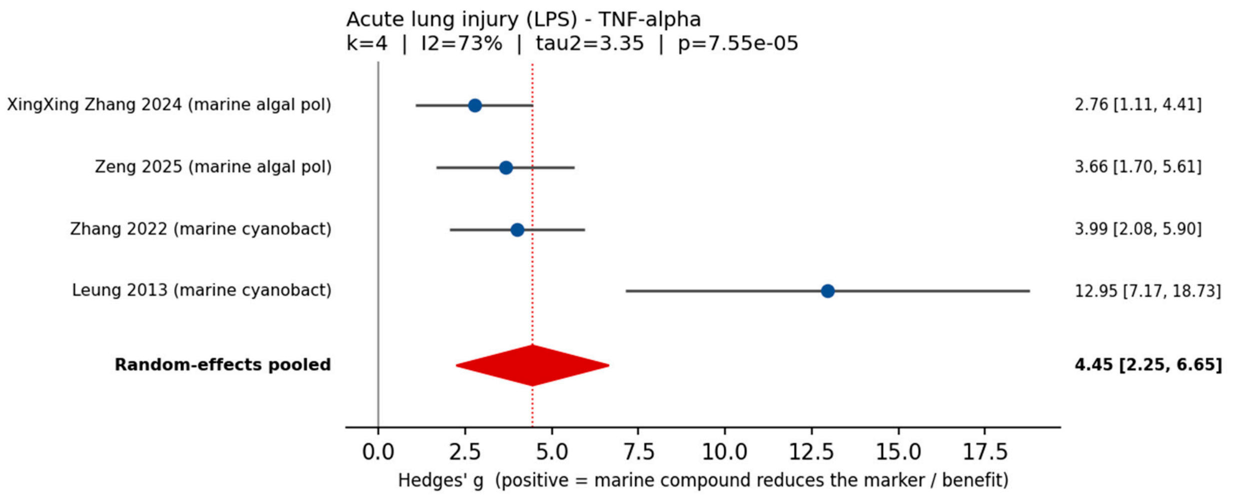

Figure S5. Acute lung injury (LPS) - TNF-alpha (k=4).

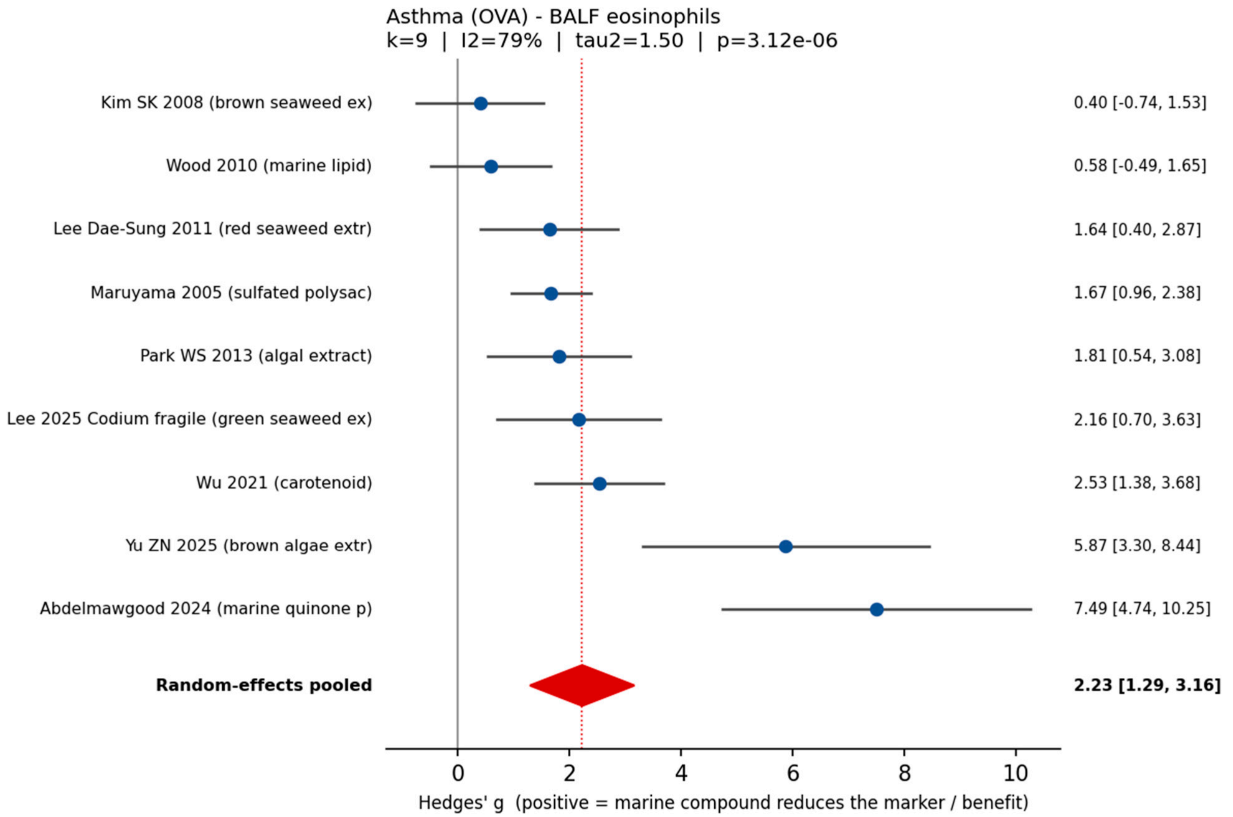

Figure S6. Asthma (OVA) - BALF eosinophils (k=9).

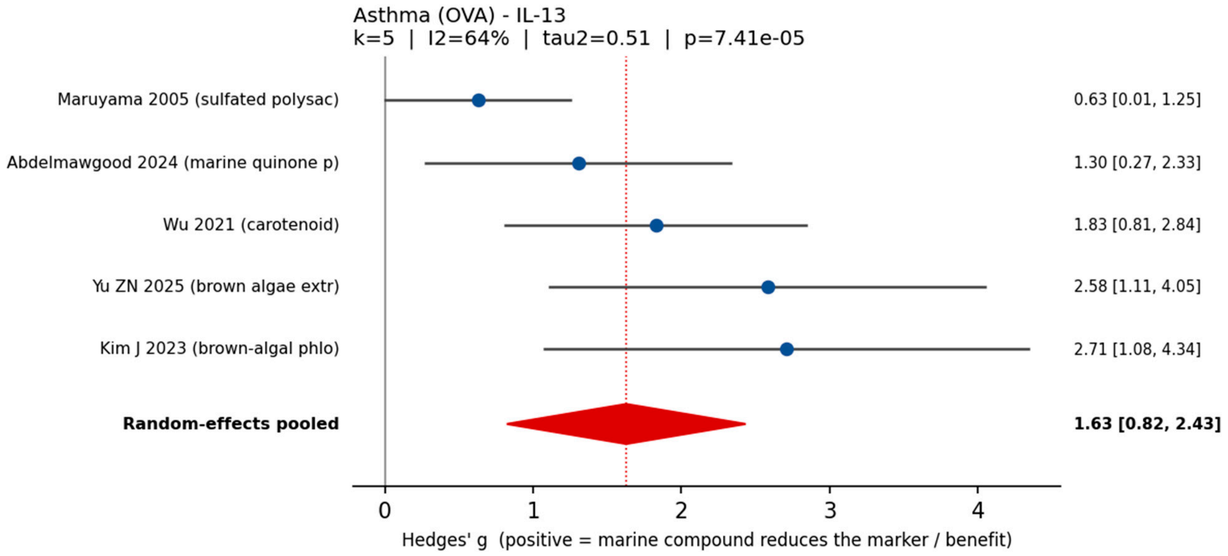

Figure S7. Asthma (OVA) - IL-13 (k=5).

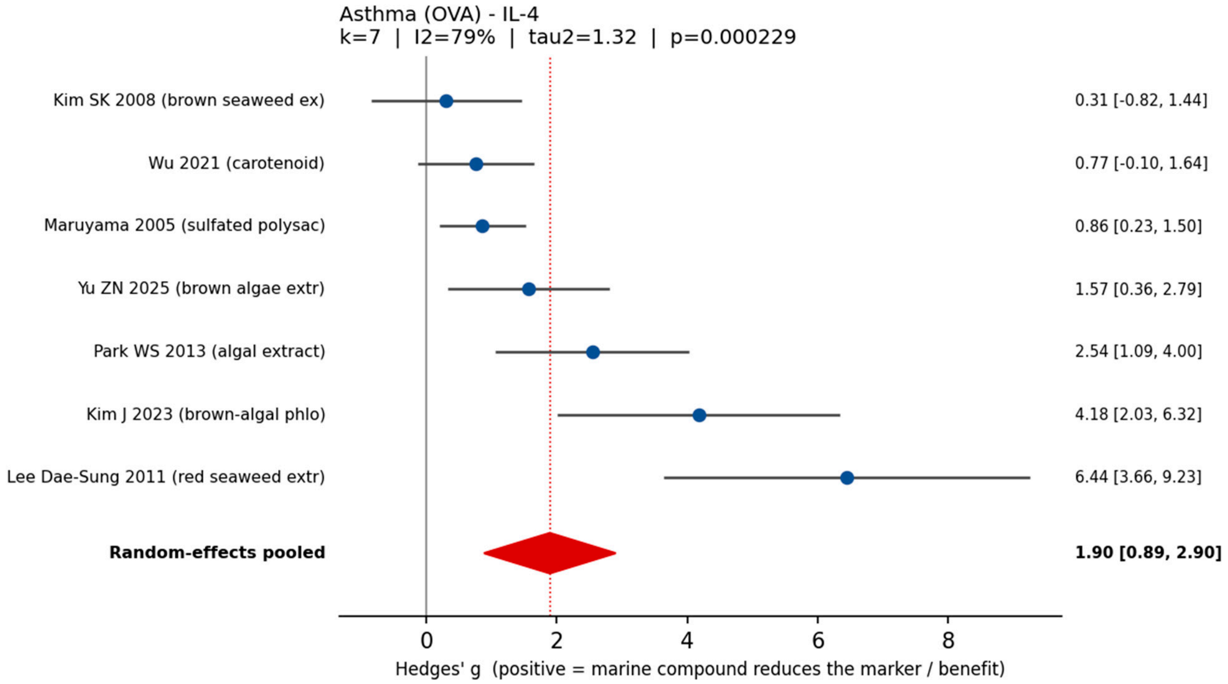

Figure S8. Asthma (OVA) - IL-4 (k=7).

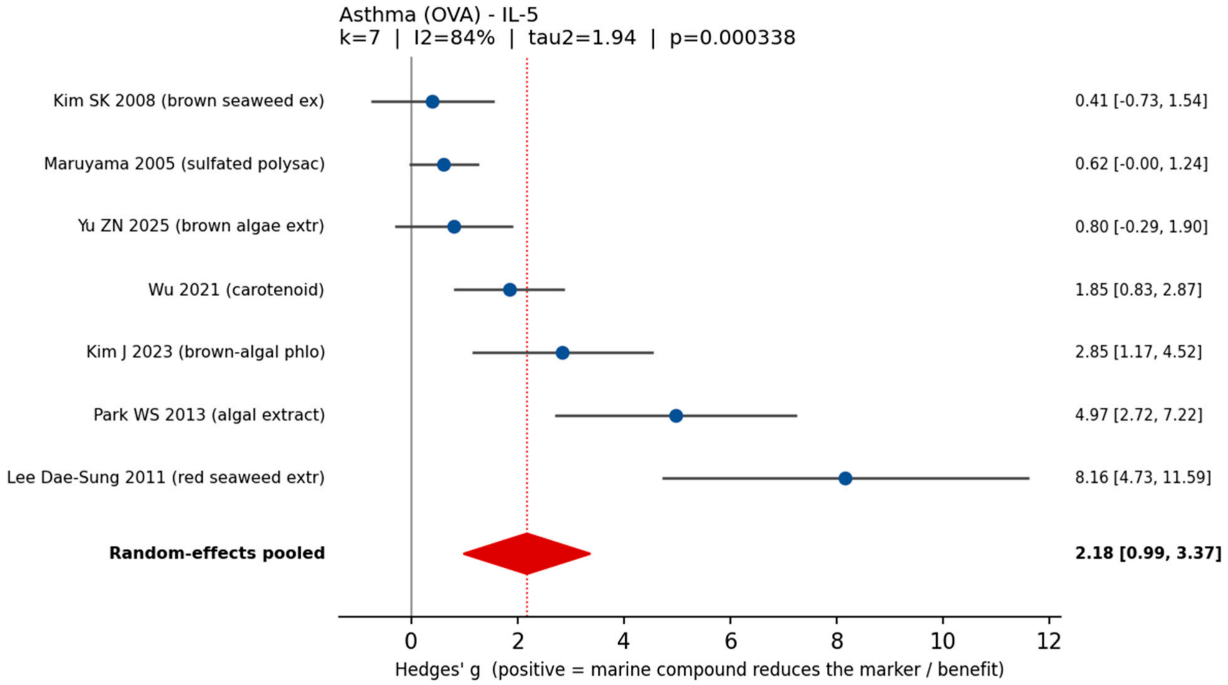

Figure S9. Asthma (OVA) - IL-5 (k=7).

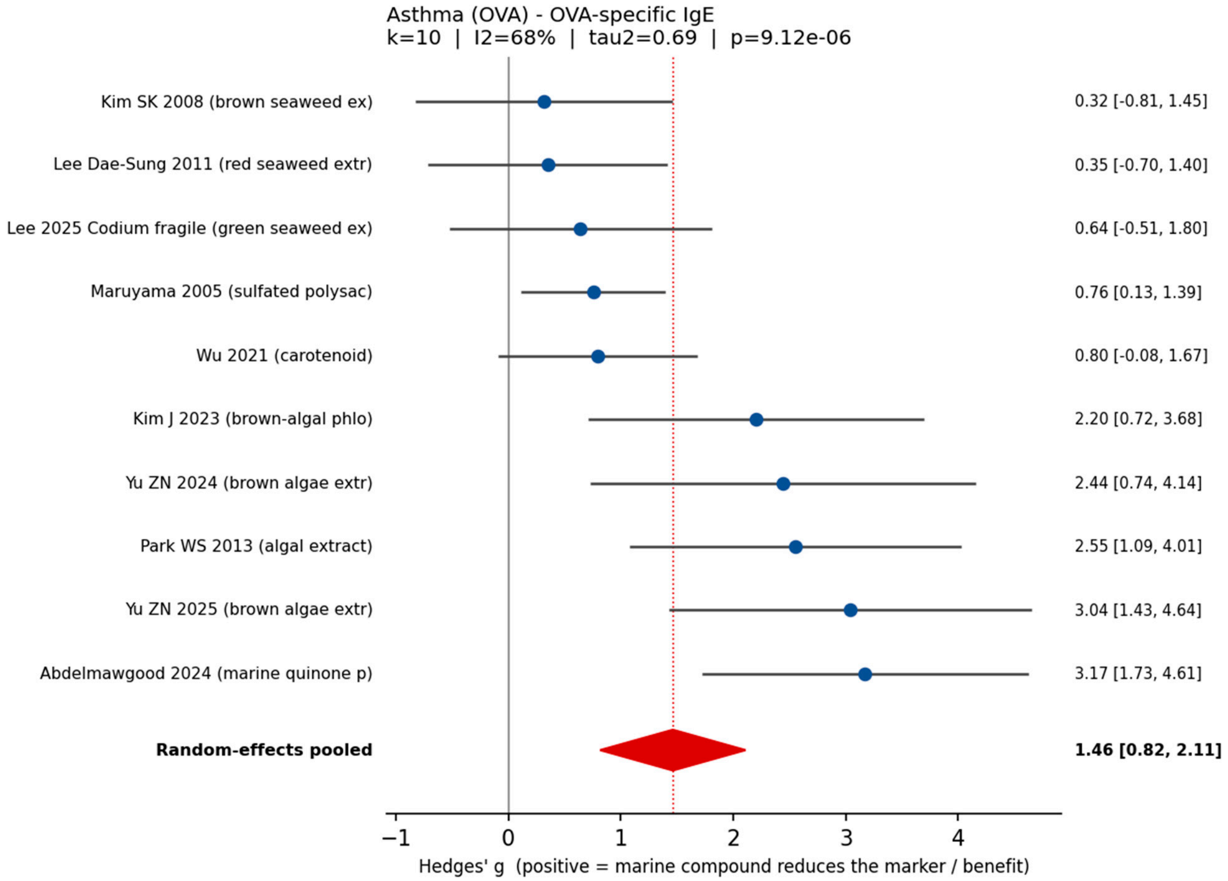

Figure S10. Asthma (OVA) - OVA-specific IgE (k=10).

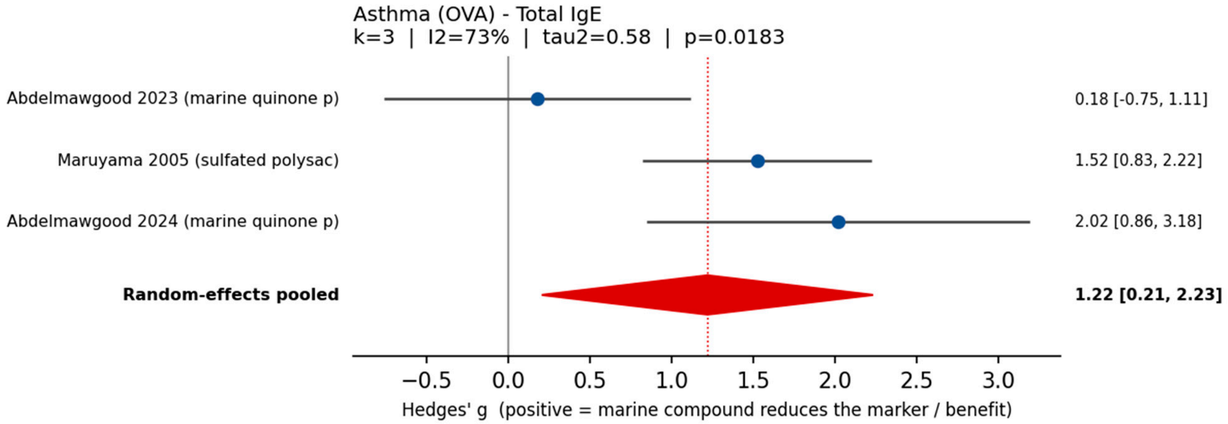

Figure S11. Asthma (OVA) - Total IgE (k=3).

Table S7. GRADE certainty of evidence (Summary of Findings)

Certainty of evidence rated with the GRADE approach adapted for preclinical studies. Starting from high certainty, ratings were downgraded for risk of bias, indirectness (animal models / surrogate biomarker outcomes), inconsistency (I-squared), imprecision (few studies) and suspected publication bias (small-study effects).

Cluster	Outcome	k	Pooled effect (g [95% CI])	GRADE certainty	Reasons for downgrading
Acute lung injury (LPS)	IL-1beta	3	g=3.62 [0.41, 6.84]	Very low	risk of bias (-1: all studies unclear/high RoB); indirectness (-1: animal models, surrogate outcomes); inconsistency (-1: I2=88%); imprecision (-1: only k=3 studies); publication bias (-1: small-study effects suspected)
Acute lung injury (LPS)	IL-6	4	g=3.06 [0.98, 5.15]	Very low	risk of bias (-1: all studies unclear/high RoB); indirectness (-1: animal models, surrogate outcomes); inconsistency (-1: I2=82%); imprecision (-1: only k=4 studies); publication bias (-1: small-study effects suspected)
Acute lung injury (LPS)	Lung wet/dry ratio	5	g=2.85 [1.87, 3.83]	Very low	risk of bias (-1: all studies unclear/high RoB); indirectness (-1: animal models, surrogate outcomes); publication bias (-1: small-study effects suspected)
Acute lung injury (LPS)	MPO	3	g=2.13 [1.31, 2.96]	Very low	risk of bias (-1: all studies unclear/high RoB); indirectness (-1: animal models, surrogate outcomes); imprecision (-1: only k=3 studies); publication bias (-1: small-study effects suspected)
Acute lung injury (LPS)	TNF-alpha	4	g=4.45 [2.25, 6.65]	Very low	risk of bias (-1: all studies unclear/high RoB); indirectness (-1: animal models, surrogate outcomes); imprecision (-1: only k=4 studies); publication bias (-1: small-study effects suspected)
Asthma (OVA)	BALF eosinophils	9	g=2.23 [1.29, 3.16]	Very low	risk of bias (-1: all studies unclear/high RoB); indirectness (-1: animal models, surrogate outcomes); inconsistency (-1: I2=79%); publication bias (-1: small-study effects suspected)
Asthma (OVA)	IL-13	5	g=1.63 [0.82, 2.43]	Very low	risk of bias (-1: all studies unclear/high RoB); indirectness (-1: animal models, surrogate outcomes); publication bias (-1: small-study effects suspected)
Asthma (OVA)	IL-4	7	g=1.90 [0.89, 2.90]	Very low	risk of bias (-1: all studies unclear/high RoB); indirectness (-1: animal models, surrogate outcomes); inconsistency (-1: I2=79%); publication bias (-1: small-study effects suspected)
Asthma (OVA)	IL-5	7	g=2.18 [0.99, 3.37]	Very low	risk of bias (-1: all studies unclear/high RoB); indirectness (-1: animal models, surrogate outcomes); inconsistency (-1: I2=84%); publication bias (-1: small-study effects suspected)
Asthma (OVA)	OVA-specific IgE	10	g=1.46 [0.82, 2.11]	Very low	risk of bias (-1: all studies unclear/high RoB); indirectness (-1: animal models, surrogate outcomes); publication bias (-1: small-study effects suspected)
Asthma (OVA)	Total IgE	3	g=1.22 [0.21, 2.23]	Very low	risk of bias (-1: all studies unclear/high RoB); indirectness (-1: animal models, surrogate outcomes); imprecision (-1: only k=3 studies); publication bias (-1: small-study effects suspected)

Across all 11 meta-analysed outcomes the GRADE certainty of evidence was Very low: the pooled estimates are consistent in direction and statistically significant, but require confirmation in standardised, pre-registered preclinical studies before any clinical inference.

Table S8. Post hoc analyses

Additional analyses, not prespecified in the registered protocol, were performed to examine whether the pooled preclinical estimates were driven by particular types of intervention. Each pooled outcome was stratified (i) by chemical class of the tested intervention and (ii) by preparation type (isolated compound or purified constituent versus crude or multicomponent preparation). DerSimonian–Laird random-effects estimates were computed within each stratum (single-study strata report the individual study estimate) and between-stratum differences were assessed with the Q-test for subgroup differences. Leave-one-out analyses and analyses excluding studies rated at high risk of bias (SYRCLE; Table S2) were also performed. Compound-specific meta-analyses were not feasible because no single compound or preparation had been evaluated in at least three independent studies for the same model–outcome combination; within-compound estimates (C-phycocyanin, echinochrome A) are shown where two studies were available. Positive Hedges' g indicates a reduction of the pathological marker by the marine-derived intervention relative to the disease-model control.

Table S8A. Pooled preclinical outcomes stratified by chemical class and by preparation type.

Cluster	Outcome	Stratum	k	Hedges' g [95% CI]	I² (%)	Contributing studies	p (subgroup difference)
**Acute lung injury (LPS)**	**IL-1β**	**All studies (primary analysis)**	**3**	**3.62 [0.41, 6.84]**	**88**		
	Chemical class:	Phycobiliprotein (C-phycocyanin)	2	4.30 [-2.82, 11.41]	92	Zhang 2022 [64]; Leung 2013 [63]	0.782
		Polysaccharide	1	3.26 [1.45, 5.07]	–	XingXing Zhang 2024 [62]	
	Preparation type:	Isolated compound or purified constituent	2	4.30 [-2.82, 11.41]	92	Zhang 2022 [64]; Leung 2013 [63]	0.782
		Crude or multicomponent preparation	1	3.26 [1.45, 5.07]	–	XingXing Zhang 2024 [62]	
**Acute lung injury (LPS)**	**IL-6**	**All studies (primary analysis)**	**4**	**3.06 [0.98, 5.15]**	**82**		
	Chemical class:	Phycobiliprotein (C-phycocyanin)	2	6.24 [-3.81, 16.29]	93	Zhang 2022 [64]; Leung 2013 [63]	0.443
		Polysaccharide	2	2.25 [0.56, 3.94]	57	Zeng 2025 [61]; XingXing Zhang 2024 [62]	
	Preparation type:	Isolated compound or purified constituent	3	3.25 [0.45, 6.06]	86	Zhang 2022 [64]; Zeng 2025 [61]; Leung 2013 [63]	0.994
		Crude or multicomponent preparation	1	3.24 [1.43, 5.05]	–	XingXing Zhang 2024 [62]	
**Acute lung injury (LPS)**	**Lung W/D ratio**	**All studies (primary analysis)**	**5**	**2.85 [1.87, 3.83]**	**44**		
	Chemical class:	Phycobiliprotein (C-phycocyanin)	2	2.88 [0.62, 5.13]	72	Leung 2013 [63]; Zhang 2022 [64]	0.885
		Polysaccharide	2	2.76 [0.73, 4.80]	64	Zeng 2025 [61]; XingXing Zhang 2024 [62]	
		Carotenoid (fucoxanthin)	1	3.36 [1.77, 4.94]	–	Li X 2021 [19]	
	Preparation type:	Isolated compound or purified constituent	4	2.65 [1.60, 3.70]	46	Leung 2013 [63]; Zeng 2025 [61]; Li X 2021 [19]; Zhang 2022 [64]	0.268
		Crude or multicomponent preparation	1	3.96 [1.89, 6.03]	–	XingXing Zhang 2024 [62]	
**Acute lung injury (LPS)**	**MPO**	**All studies (primary analysis)**	**3**	**2.13 [1.31, 2.96]**	**0**		
	Chemical class:	Polysaccharide	1	1.66 [0.32, 3.00]	–	XingXing Zhang 2024 [62]	0.375
		Phycobiliprotein (C-phycocyanin)	2	2.43 [1.38, 3.48]	0	Zhang 2022 [64]; Leung 2013 [63]	
	Preparation type:	Crude or multicomponent preparation	1	1.66 [0.32, 3.00]	–	XingXing Zhang 2024 [62]	0.375
		Isolated compound or purified constituent	2	2.43 [1.38, 3.48]	0	Zhang 2022 [64]; Leung 2013 [63]	
**Acute lung injury (LPS)**	**TNF-α**	**All studies (primary analysis)**	**4**	**4.45 [2.25, 6.65]**	**73**		
	Chemical class:	Polysaccharide	2	3.13 [1.87, 4.40]	0	XingXing Zhang 2024 [62]; Zeng 2025 [61]	0.276
		Phycobiliprotein (C-phycocyanin)	2	8.04 [-0.70, 16.78]	88	Zhang 2022 [64]; Leung 2013 [63]	

Cluster	Outcome	Stratum	k	Hedges' g [95% CI]	I² (%)	Contributing studies	p (subgroup difference)
	Preparation type:	Crude or multicomponent preparation	1	2.76 [1.11, 4.41]	–	XingXing Zhang 2024 [62]	0.134
		Isolated compound or purified constituent	3	5.57 [2.29, 8.86]	78	Zeng 2025 [61]; Zhang 2022 [64]; Leung 2013 [63]	
**Asthma (OVA)**	**BALF eosinophils**	**All studies (primary analysis)**	**9**	**2.23 [1.29, 3.16]**	**79**		
	Chemical class:	Polyphenol/phlorotannin	3	1.25 [0.35, 2.14]	39	Kim SK 2008 [49]; Lee Dae-Sung 2011 [47]; Park WS 2013 [48]	<0.001
		Marine lipid extract	1	0.58 [-0.49, 1.65]	–	Wood 2010 [51]	
		Polysaccharide	1	1.67 [0.96, 2.38]	–	Maruyama 2005 [46]	
		Crude seaweed extract	2	3.86 [0.24, 7.48]	83	Lee 2025 *Codium*; Yu ZN 2025 [57]	
		Carotenoid (fucoxanthin)	1	2.53 [1.38, 3.68]	–	Wu 2021 [18]	
		Naphthoquinone pigment (echinochrome A)	1	7.49 [4.74, 10.25]	–	Abdelmawgood 2024 [28]	
	Preparation type:	Crude or multicomponent preparation	6	1.74 [0.68, 2.80]	73	Kim SK 2008 [49]; Wood 2010 [51]; Lee Dae-Sung 2011 [47]; Park WS 2013 [48]; Lee 2025*Codium*; Yu ZN 2025 [57]	0.173
		Isolated compound or purified constituent	3	3.40 [1.26, 5.55]	88	Maruyama 2005 [46]; Wu 2021 [18]; Abdelmawgood 2024 [28]	
**Asthma (OVA)**	**IL-13**	**All studies (primary analysis)**	**5**	**1.63 [0.82, 2.43]**	**64**		
	Chemical class:	Polysaccharide	1	0.63 [0.01, 1.25]	–	Maruyama 2005 [46]	0.023
		Naphthoquinone pigment (echinochrome A)	1	1.30 [0.27, 2.33]	–	Abdelmawgood 2024 [28]	
		Carotenoid (fucoxanthin)	1	1.83 [0.82, 2.85]	–	Wu 2021 [18]	
		Crude seaweed extract	1	2.58 [1.11, 4.05]	–	Yu ZN 2025 [57]	
		Polyphenol/phlorotannin	1	2.71 [1.08, 4.34]	–	Kim J 2023 [41]	
	Preparation type:	Isolated compound or purified constituent	4	1.43 [0.61, 2.25]	63	Maruyama 2005 [46]; Abdelmawgood 2024 [28]; Wu 2021 [18]; Kim J 2023 [41]	0.181
		Crude or multicomponent preparation	1	2.58 [1.11, 4.05]	–	Yu ZN 2025 [57]	
**Asthma (OVA)**	**IL-4**	**All studies (primary analysis)**	**7**	**1.90 [0.89, 2.90]**	**79**		
	Chemical class:	Polyphenol/phlorotannin	4	3.14 [0.75, 5.52]	87	Kim SK 2008 [49]; Park WS 2013 [48]; Kim J 2023 [41]; Lee Dae-Sung 2011 [47]	0.224
		Carotenoid (fucoxanthin)	1	0.77 [-0.10, 1.64]	–	Wu 2021 [18]	
		Polysaccharide	1	0.86 [0.23, 1.50]	–	Maruyama 2005 [46]	
		Crude seaweed extract	1	1.57 [0.36, 2.79]	–	Yu ZN 2025 [57]	
	Preparation type:	Crude or multicomponent preparation	4	2.36 [0.53, 4.19]	84	Kim SK 2008 [49]; Yu ZN 2025 [57]; Park WS 2013 [48]; Lee Dae-Sung 2011 [47]	0.442

Cluster	Outcome	Stratum	k	Hedges' g [95% CI]	I² (%)	Contributing studies	p (subgroup difference)
		Isolated compound or purified constituent	3	1.48 [0.23, 2.74]	78	Wu 2021 [18]; Maruyama 2005 [46]; Kim J 2023 [41]	
**Asthma (OVA)**	**IL-5**	**All studies (primary analysis)**	**7**	**2.18 [0.99, 3.37]**	**84**		
	Chemical class:	Polyphenol/phlorotannin	4	3.79 [0.91, 6.67]	89	Kim SK 2008 [49]; Kim J 2023 [41]; Park WS 2013 [48]; Lee Dae-Sung 2011 [47]	0.049
		Polysaccharide	1	0.62 [0.00, 1.24]	–	Maruyama 2005 [46]	
		Crude seaweed extract	1	0.80 [-0.29, 1.90]	–	Yu ZN 2025 [57]	
		Carotenoid (fucoxanthin)	1	1.85 [0.83, 2.87]	–	Wu 2021 [18]	
	Preparation type:	Crude or multicomponent preparation	4	3.12 [0.58, 5.67]	90	Kim SK 2008 [49]; Yu ZN 2025 [57]; Park WS 2013 [48]; Lee Dae-Sung 2011 [47]	0.291
		Isolated compound or purified constituent	3	1.60 [0.36, 2.84]	77	Maruyama 2005 [46]; Wu 2021 [18]; Kim J 2023 [41]	
**Asthma (OVA)**	**OVA-specific IgE**	**All studies (primary analysis)**	**10**	**1.46 [0.82, 2.11]**	**68**		
	Chemical class:	Polyphenol/phlorotannin	4	1.26 [0.13, 2.39]	69	Kim SK 2008 [49]; Lee Dae-Sung 2011 [47]; Kim J 2023 [41]; Park WS 2013 [48]	0.030
		Crude seaweed extract	3	1.95 [0.40, 3.50]	70	Lee 2025*Codium*; Yu ZN 2024 [56]; Yu ZN 2025 [57]	
		Polysaccharide	1	0.76 [0.13, 1.39]	–	Maruyama 2005 [46]	
		Carotenoid (fucoxanthin)	1	0.80 [-0.07, 1.68]	–	Wu 2021 [18]	
		Naphthoquinone pigment (echinochrome A)	1	3.17 [1.73, 4.61]	–	Abdelmawgood 2024 [28]	
	Preparation type:	Crude or multicomponent preparation	6	1.43 [0.47, 2.39]	69	Kim SK 2008 [49]; Lee Dae-Sung 2011 [47]; Lee 2025*Codium*; Yu ZN 2024 [56]; Park WS 2013 [48]; Yu ZN 2025 [57]	0.855
		Isolated compound or purified constituent	4	1.56 [0.55, 2.57]	74	Maruyama 2005 [46]; Wu 2021 [18]; Kim J 2023 [41]; Abdelmawgood 2024 [28]	
**Asthma (OVA)**	**Total IgE**	**All studies (primary analysis)**	**3**	**1.22 [0.21, 2.23]**	**73**		
	Chemical class:	Naphthoquinone pigment (echinochrome A)	2	1.07 [-0.74, 2.87]	83	Abdelmawgood 2023 [27]; Abdelmawgood 2024 [28]	0.645
		Polysaccharide	1	1.52 [0.82, 2.21]	–	Maruyama 2005 [46]	
	Preparation type:	Isolated compound or purified constituent	3	1.22 [0.21, 2.23]	73	Abdelmawgood 2023 [27]; Maruyama 2005 [46]; Abdelmawgood 2024 [28]	n.a.

n.a., not applicable (single stratum). Chemical classes: Polysaccharide (sulfated fucoidan, laminarin, unidentified algal polysaccharides); Polyphenol/phlorotannin (phlorotannin- or polyphenol-rich algal extracts and trifuhalol A); Crude seaweed extract (*Codium fragile* hot-water extract; *Undaria pinnatifida* ethanolic extract); Phycobiliprotein (C-phycocyanin); Carotenoid (fucoxanthin); Naphthoquinone pigment (echinochrome A); Marine lipid extract (Lyprinol).

Table S8B. Leave-one-out and risk-of-bias sensitivity analyses of the pooled preclinical outcomes.

Cluster	Outcome	Leave-one-out range of pooled Hedges' g	Excluding studies at high risk of bias
Acute lung injury (LPS)	IL-1β	1.96 (omitting Leung 2013 [63]) to 5.42 (omitting Zhang 2022 [64]); non-significant when omitting XingXing Zhang 2024 [62] or Leung 2013 [63]	k = 2; 4.30 [-2.82, 11.41]; I^2 = 92%; p = 0.236
Acute lung injury (LPS)	IL-6	1.87 (omitting Leung 2013 [63]) to 4.36 (omitting Zhang 2022 [64]); significance retained in all iterations	k = 3; 3.25 [0.45, 6.06]; I^2 = 86%; p = 0.023
Acute lung injury (LPS)	Lung W/D ratio	2.58 (omitting Zhang 2022 [64]) to 3.17 (omitting Leung 2013 [63]); significance retained in all iterations	k = 3; 2.44 [1.14, 3.74]; I^2 = 53%; p < 0.001
Acute lung injury (LPS)	MPO	1.92 (omitting Leung 2013 [63]) to 2.43 (omitting XingXing Zhang 2024 [62]); significance retained in all iterations	k = 2; 2.43 [1.38, 3.48]; I^2 = 0%; p < 0.001
Acute lung injury (LPS)	TNF-α	3.39 (omitting Leung 2013 [63]) to 5.57 (omitting XingXing Zhang 2024 [62]); significance retained in all iterations	k = 3; 5.57 [2.29, 8.86]; I^2 = 78%; p < 0.001
Asthma (OVA)	BALF eosinophils	1.78 (omitting Abdelmawgood 2024 [28]) to 2.48 (omitting Kim SK 2008 [49]); significance retained in all iterations	k = 8; 2.27 [1.23, 3.31]; I^2 = 82%; p < 0.001
Asthma (OVA)	IL-13	1.43 (omitting Yu ZN 2025 [57]) to 1.90 (omitting Maruyama 2005 [46]); significance retained in all iterations	No high-risk study contributed
Asthma (OVA)	IL-4	1.40 (omitting Lee Dae-Sung 2011 [47]) to 2.25 (omitting Maruyama 2005 [46]); significance retained in all iterations	No high-risk study contributed
Asthma (OVA)	IL-5	1.60 (omitting Lee Dae-Sung 2011 [47]) to 2.64 (omitting Maruyama 2005 [46]); significance retained in all iterations	No high-risk study contributed
Asthma (OVA)	OVA-specific IgE	1.26 (omitting Abdelmawgood 2024 [28]) to 1.61 (omitting Lee Dae-Sung 2011 [47]); significance retained in all iterations	k = 9; 1.57 [0.86, 2.28]; I^2 = 70%; p < 0.001
Asthma (OVA)	Total IgE	0.89 (omitting Abdelmawgood 2024 [28]) to 1.65 (omitting Abdelmawgood 2023 [27]); non-significant when omitting Maruyama 2005 [46] or Abdelmawgood 2024 [28]	No high-risk study contributed

High risk of bias (SYRCLE) among contributing studies: XingXing Zhang 2024 [62], Li X 2021 [19], Lee 2025 (*Codium fragile*) [40].
